# Nutritional Assessment of Ready-to-Eat Salads in German Supermarkets: Comparison of the nutriRECIPE-Index and the Nutri-Score

**DOI:** 10.3390/foods11244011

**Published:** 2022-12-11

**Authors:** Laura Schlarbaum, Frank Forner, Kristin Bohn, Michael Amberg, Patrick Mäder, Stefan Lorkowski, Toni Meier

**Affiliations:** 1 Institute of Nutritional Sciences, Friedrich Schiller University Jena, Dornburger Straße 25, 07743 Jena, Germany; 2 Competence Cluster for Nutrition and Cardiovascular Health (nutriCARD) Halle-Jena-Leipzig, 07743 Jena, Germany; 3 Institute for Agricultural and Nutritional Sciences, Martin Luther University Halle-Wittenberg, Von-Danckelmann-Platz 2, 06120 Halle (Saale), Germany; 4 Data-Intensive Systems and Visualization, Technische Universität Ilmenau, 98684 Ilmenau, Germany; 5 Institute for Sustainable Agriculture and Food Economics (INL) e.V., 06120 Halle (Saale), Germany

**Keywords:** nutriRECIPE-Index, Nutri-Score, nutrition labels, ready-to-eat salads, front-of-pack nutrition labeling

## Abstract

Globally, an unbalanced diet causes more deaths than any other factor. Due to a lack of knowledge, it is difficult for consumers to select healthy foods at the point of sale. Although different front-of-pack labeling schemes exist, their informative value is limited due to small sets of considered parameters and lacking information on ingredient composition. We developed and evalauated a manufacture-independent approach to quantify ingredient composition of 294 ready-to eat salads (distinguished into 73 subgroups) as test set. Nutritional quality was assessed by the nutriRECIPE-Index and compared to the Nutri-Score. The nutriRECIPE-Index comprises the calculation of energy-adjusted nutrient density of 16 desirable and three undesirable nutrients, which are weighted according to their degree of supply in the population. We show that the nutriRECIPE-Index has stronger discriminatory power compared to the Nutri-Score and discriminates as well or even better in 63 out of the 73 subgroups. This was evident in groups where seemingly similar products were compared, e.g., potato salads (Nutri-Score: C only, nutriRECIPE-Index: B, C and D). Moreover, the nutriRECIPE-Index is adjustable to any target population’s specific needs and supply situation, such as seniors, and children. Hence, a more sophisticated distinction between single food products is possible using the nutriRECIPE-Index.

## 1. Introduction

Worldwide, a suboptimal diet is responsible for more deaths than any other cause [1]. With an increasing trend, one in five deaths is attributable to dietary risk factors and could potentially be prevented. Thus, improving the nutritional status has an enormous preventive potential [2]. Reductions in sugar, saturated fat, and sodium intake have often been proposed to improve nutritional conditions [3]. However, from a current perspective, measures to increase the intake of specific nutrients and food groups, such as fiber, polyunsaturated fatty acids (PUFA), or vegetables, seem to have tremendous potential [2]. As the complex organization of everyday life and the pressure it applies on food selection is increasing, so is the consumption of ready-to-eat products [4]. Therefore, the consumption of ready-to-eat salads containing desirable food components could be a suitable solution for improving one’s nutritional status. Although many people claim to look out for healthy choices when eating out [5], it has been shown that high out-of-home consumption is associated with increased body weight [6,7]. This appears to be mainly due to a lack of relevant information at the point of sale [8,9]. Moreover, the definition of salads described in the guiding principles of the Commission of the German Food Code shows that they can vary widely in food retailing [10]. Thus, improved food information can nudge consumers towards more healthy purchasing decisions [11,12]. For this purpose, several labeling schemes were developed (e.g., nutriRECIPE-Index [13], Nutri-Score [14], NuVal [15], Keyhole [16], and Health Star Rating [17]), while in Europe the Nutri-Score is most widely used [18]. However, within food categories the potential of the Nutri-Score to discriminate between products is limited [19].

It is therefore necessary to evaluate whether front-of-pack food labels such as the nutriRECIPE-Index and the Nutri-Score are suitable for appropriately reflecting the physiological nutritional value of a food product. Further, pre-packaged foods such as ready-to-eat salads rely on information provided by the manufacturers. The mandatory European regulation (EU) No. 1169/2011 applies in all EU countries [20]. However, companies are only obliged to state the content of monounsaturated fatty acids (MUFA), PUFA, starch, fiber, vitamins, and minerals in food. Only the Big7 information, which includes energy in kJ and kcal and total fat, saturated fatty acids (SFA), carbohydrates, sugar, protein, and salt in g per 100 g or 100 mL, are mandatory [20]. It is up to the manufacturers to determine whether the nutritional information is based on food analyses or calculations. The calculation may be based on generally proven and accepted data or utilizing known or actual average values of the ingredients used [20]. Deviations between the stated values and those determined by official inspections are permitted within a tolerance range [21]. In addition to the nutritional information, the European regulation (EU) No. 1169/2011 requires printing an ingredients list. The ingredients are named in descending order according to their proportion in the product. The obligation to indicate the specific percentage quantity of an ingredient only applies to cases where the ingredient is part of the product name, is particularly emphasized by pictures and words on the front of the packaging, or is of essential importance for the characterization of a food. The quantities of all other ingredients remain optional [20]. In this context, we compared the nutriRECIPE-Index developed by our group with the Nutri-Score, reflecting different mathematical approaches in the calculation framework using these regulatory requirements. Thus, the present study has the following aims:

1. To compare the results of the nutriRECIPE-Index with the results of the Nutri-Score based on 294 ready-to-eat salads in German supermarkets (on the single product level and the product group level),
2. To test—regarding the nutriRECIPE-Index—two different approaches to match the components of the ingredient list (disaggregated approach or aggregated approach),
3. To underline the strengths and to discuss the limitations of the nutriRECIPE-Index and the Nutri-Score for helping consumers towards more healthy purchasing decisions.

## 2. Materials and Methods

### 2.1. Data Collection and Sample Preparation

According to the guiding principles of the Commission of the German Food Code of the German Federal Ministry for Food and Agriculture, the here considered salads are defined as ready-to-eat salads made from ingredients of animal and/or vegetable origin in a sauce that is flavored to complement them. The character of the salads is determined by the recipe, the type and the nature of the ingredients and the method of production. Requirements for minimum or maximum quantities of ingredients must be followed during production [10]. The food items considered in this study comprise 294 ready-to-eat salads documented in German supermarkets from 12 February 2019 to 4 June 2020. The data was recorded supermarkets in the German cities Erfurt, Frankfurt am Main, Jena, Kiel, Reichensachsen, Weimar and Worms. Products from the supermarkets Aldi Nord, Aldi Süd, Alnatura, Denns BioMarkt (dennree), dm-drogerie markt, Edeka, Famila Nordost, Globus, Herkules E-Center, Kaufland, Lidl, Müller, Nahkauf, Netto Marken-Discount, Norma, Penny, Rewe and Tegut were considered. In addition, the study sample was extended by salads from the online shops of Alnatura [22], Bringmeister [23], dm-drogerie markt [24], Edeka Schubert [25], Lieferello [26], natsu Foods [27], Rewe [28] and Vekoop [29]. Product prices from natsu Foods were inquired by telephone. The salads were photographed in the supermarkets or documented by screenshots in the case of online shops. The goal of the data collection was to acquire a large test set of ready-to-eat salads as possible to increase the validity of the results. Thus, all ready-to-eat salads available in the mentioned period and supermarkets were included in the data collection. The characteristics listed on the products were then recorded in a table (Supplementary Table S1). To ensure the digital readability of the ingredient list by the algorithm (Section 2.2), it had to be recorded following predefined formatting rules [30]. Two versions of the ingredient lists were created for each of the salads recorded. While the registration variant disaggregated ingredients (henceforth disaggregated approach) were formatted exclusively according to the scheme mentioned above, a modification was used for the variant aggregated ingredients (henceforth aggregated approach). Here, aggregated ingredients consisting of sub-ingredients were registered without listing these sub-ingredients. The reduction was performed exclusively on aggregated ingredients for which an equivalent counterpart was available in the German Nutrient Database (Bundeslebensmittelschlüssel; GND, Max Rubner-Institut). Percentage quantities of the original ingredient list were adjusted following reduction to aggregated ingredients. Following the calculations, manufacturers were contacted via mail and telephone to assess whether they determined the Big7 data by analyses or literature data (Supplementary Table S2).

### 2.2. Calculation of the Ingredient Quantity

**Figure 1:**
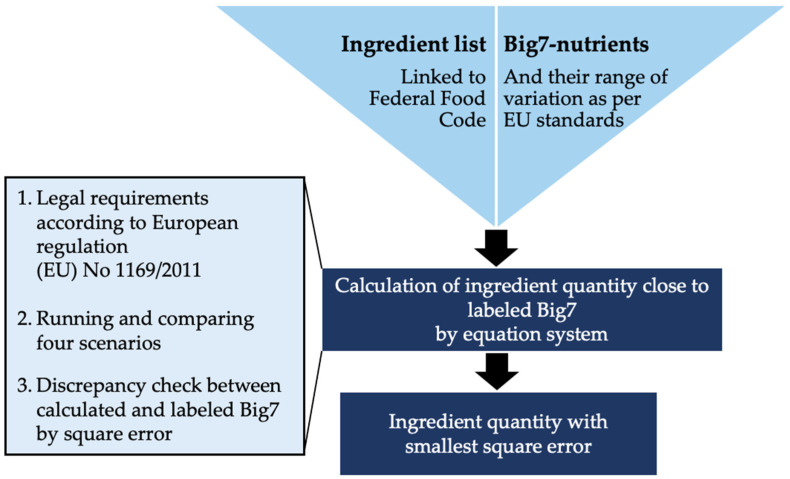
Calculation of ingredient quantity. Schematic presentation of the determination of ingredient proportions using the algorithm developed by Bohn et al. [31].

The quantity of each ingredient in the product was calculated according to Bohn et al. [31] to enable health assessment using the nutriRECIPE-Index (Figure 1). The algorithm calculates the proportion of the respective sub-ingredients in the overall product with the disaggregated approach. In the case of the aggregated approach, it calculates the ingredient proportions solely based on the aggregated ingredients. The algorithm used the digitally readable ingredient list and the Big7 information provided by the manufacturers, including the range of variation of the Big7 allowed in the EU [21], to calculate the ingredient compositions. The individual elements of the ingredients list were matched with the corresponding data of the GND Version 3.02 via their GND code [31]. Furthermore, the GND data are supplemented with data from the US Department of Agriculture (USDA) [32]. The product’s composition was calculated using a system of linear equations constrained by additional conditions [31]. The calculations’ basis is the discrepancy between the calculated and the labeled Big7, expressed as an absolute difference. These differences are squared and summed up to give the square error. The algorithm decides on the combination of ingredients and the scenario with the lowest square error. Based on the ingredient proportions calculations, the product’s respective nutrient quantity can now be determined due to the link with the GND [31]. The nutrient quantities were calculated twice for each salad recorded, once using the disaggregated approach and the aggregated approach, alternatively.

### 2.3. Classification of the Recorded Salads

Following the recording of all salads, the entire sample was divided into main groups and subgroups. The main groups were clustered according to their base composition. For statistical evaluation, criteria for each main group were defined, according to which subgroups were formed. A minimum of seven salads per subgroup was set to ensure a reasonable statistical comparison.

### 2.4. Calculation of the nutriRECIPE-Index

The calculation of the nutriRECIPE-Index was based upon the framework developed by Forner et al. for the evaluation of canteen dishes [13]. Whereas in Forner et al. the functional unit is one serving of dish, we slightly adjusted the formulas used to refer here to 100 g of product to be consistent with the Big7 reference unit. The nutriRECIPE-Index value is calculated based on nutrient scores and respective weighting factors for 19 nutrients of which 16 are potentially qualifying (protein, fiber, mono- and poly-unsaturated fatty acids, vitamins B_1_, B_2_, B_6_, B_12_, C, D, E, folate, calcium, magnesium, iron, zinc, iodine) and three potentially disqualifying nutrients (sodium/salt, free sugars, saturated fatty acids). The score refers to nutrient density and represents how much of a nutrient’s daily requirement is covered by a 100 g of food. The weighting factor mirrors the ratio of actual and target intake of a nutrient for an average adult in Germany. Of the 19 variables selected, 16 are categorized as qualifying nutrients, while three are considered disqualifying.. The equations for the nutriRECIPE-Index-scores for nutrients with favorable or unfavorable effects are as follows:

$$y=\left(\ln\left(\frac{N_{\mathrm{product}}}{N_{\mathrm{rec}}}\;\times \;\frac{E_{\mathrm{rec}}}{E_{\mathrm{product}}}\right)+1\right)\times \;\frac{N_{\mathrm{act}}}{N_{\mathrm{rec}}}$$

$$y=\left(-\ln\left(\frac{N_{\mathrm{product}}}{N_{\mathrm{rec}}}\;\times \;\frac{E_{\mathrm{rec}}}{E_{\mathrm{product}}}\right)\right)\times \;\frac{N_{\mathrm{act}}}{N_{\mathrm{rec}}}$$

N_product_ = Nutrient content in 100 g product

N_rec_ = Recommended nutrient intake per day

E_rec_ = Recommended energy intake per day

E_product_ = Energy content of 100 g product

N_act_ = Average nutrient intake per day

The results are standardized as a percentage to present them clearly to consumers. If the nutrient density of all nutrients is at the daily intake recommendation, this corresponds to 100%. Due to bonus points given for nutrient densities above recommendation, the value can exceed 100% [13]. For the purpose of comparability with the Nutri-Score, the nutriRECIPE-Index values ≥80% are considered an A, 60% to 79.9% a B, 40% to 59.9% a C, 20% to 39.9% a D, and <20% an E.

### 2.5. Calculation of the Nutri-Score

A template provided by the Belgian Federal Public Service was used to calculate the Nutri-Score based on seven food characteristics (Supplementary Table S3) [33]. The values per 100 g for energy, total fat, SFA, sugar, protein, and salt were taken from the packaging information. The fiber content is based on the calculations of the aggregated approach, as described in Section 2.2. To calculate the amounts of fruits, vegetables, pulses, nuts, canola oil, walnut oil, and olive oil (henceforth described as fruit content), the percentages of the ingredients list were taken. The total amount in the product was assigned to the ranges ≤40%, >40%, >60% or >80%. If quantity information was missing, the algorithm’s output using the disaggregated approach was used (Section 2.2). Depending on the point totals achieved for all product characteristics, the products were assigned to one of the five color-highlighted categories—the more advantageous the composition, the lower the total points. Total points of −15 to −1 were assigned an A, 0 to 2 a B, 3 to 10 a C, 11 to 18 a D, and 19 to 40 points an E [14].

### 2.6. Statistical Methods of Data Evaluation

Nutrient amounts in each salad group were presented using the median, given that the data were not normally distributed. The individual nutrient amounts within different main groups and subgroups of the salads were calculated and tested for differences. Since there is no presumption of effect, two-sided significance was used. A *p* < 0.05 was considered statistically significant, while *p* < 0.01 was assessed as highly significant [34]. Appropriate statistical tests were conducted depending on the data characteristics according to Field [34] and Rosenthal [35] (Supplementary Figure S1). Given that all data collected were unpaired and independent, each nutrient in each main group was initially tested for normal distribution using the Shapiro-Wilk test. When the respective nutrient was not normally distributed, the Mann-Whitney U test was used to test for significant differences between the two groups. When the sample size was less than 30 salads (n < 30), exact significance was chosen to indicate the significance level. For n ≥ 30, asymptotic significance was used. To compare more than two groups, the Kruskal-Wallis test was applied. Here, the choice of significance level was also made depending on the sample size. Since the Kruskal-Wallis test can only determine whether there are significant differences between groups, but not exactly between which groups, pairwise comparisons with Bonferroni correction were performed afterward [34]. If the nutrient considered was normally distributed, Levene’s test had to be additionally performed to test the distribution of values for variance homogeneity in the groups that were being tested. If homoscedasticity was found, a t-test was used when comparing two groups (n = 2). For n > 2, a single one-way ANOVA was used. Bonferroni’s post-hoc procedure was then performed to calculate which groups had statistical differences. On the other hand, if Levene’s test indicated heteroscedasticity in the data, Welch’s test was performed for mean comparison when n = 2 [34], and Welch’s ANOVA was performed when n > 2 [35]. Welch’s ANOVA was followed by Games-Howell’s post-hoc procedure [34]. For comparison of the two rating systems, the percentage of salads from each of the main groups and subgroups assigned in each category of the Nutri-Score and nutriRECIPE-Index were determined and presented using a plot. In addition, the mean nutriRECIPE-Index was calculated for the respective main groups and subgroups. Finally, to compare the two variants disaggregated approach and aggregated approach, the mean and median for each main group and subgroup were calculated using the two approaches. The differences between the approaches were presented in a plot.

### 2.7. Materials

Data preparation, evaluation, and presentation were carried out using Microsoft Excel for Mac version 16.40 (20081000), Microsoft Corporation (Redmond, Washington, DC, USA). Advanced statistical analysis was performed using SPSS Statistics version 26.0.0.0 from IBM (Armonk, New York, NY, USA).

## 3. Results

### 3.1. Classification of the Recorded Salads

The salads recorded (n = 294) were divided into four main groups: leaf salads, raw food salads, starch-based salads, and protein-based salads (Table 1). Leaf salads (n = 107) include all salads whose dominant ingredient is green leaf lettuce. The salads can consist exclusively of vegetables or contain a starch-based side dish, such as pasta. The main group of raw food salads (n = 53) includes all salads whose base consists of raw vegetables, such as cabbage, carrots, and cucumber. For starch-based salads (n = 89), all salads are aggregated whose main ingredient is a food with high starch content. Since this creates a heterogeneous group, the main group was divided into four subsets to ensure reasonable comparability. Semolina (n = 31) contains salads made from durum wheat semolina, such as couscous or bulgur. Furthermore, the subsets potato (n = 31), pasta (n = 18), and quinoa (n = 7) were formed. Salads based on barley or rice were included when considering the entire main group but excluded when examining the subgroups, as they could not be assigned to any of the four subsets. The main group of protein-based salads (n = 45) included all salads whose basis consisted of protein sources such as eggs, meat, or legumes.

Afterward, the individual main groups and subsets were subdivided into subgroups based on the characteristic features of the salads. The features could represent external properties such as brand, price, and composition specifics. Salads that did not meet these criteria were excluded from the assessment. The criteria for the subdivision were dietary type, presence of a dressing, base of the dressing, presence and type of a starch-based side dish, brand, and price category. Since leaf salads are the largest group, this classification could not be implemented for other main groups and subsets while maintaining a minimum number of six salads per subgroup. Therefore, only selected criteria were adopted for classification or additional criteria were defined. In the case of raw food, starch-based, and protein-based salads, a distinction was made additionally according to salad base and, in semolina, between organic and conventional salads. Since the selection of quinoa salads in Germany was limited, a classification into subgroups was not possible due to the small sample size. Accordingly, the quinoa salads are only included in their entirety for comparisons with the other subsets (semolina, potato, and pasta) or were integrated in the analysis of the main groups. Meat-based salads include only those salads whose basis is red meat or poultry. Since meat differs significantly in its nutritional composition from eggs and legumes, the group of protein-based was analyzed separately. As with the quinoa salads, the sample size of egg- and legumes-based salads were too small; thus, further differentiation was only made for meat-based salads. However, it was not possible to subdivide the main group protein-based salads in its entirety, as all egg-based salads had a mayonnaise dressing, while none of the legume salads had so that the result would be misleading. In addition, all legume-based salads were in the upper price range, whereas the meat-based salads were significantly less expensive so that there would be a bias here as well (Table 1).

### 3.2. Comparison of the nutriRECIPE-Index and Nutri-Score

#### 3.2.1. Leaf Salads

**Figure 2:**
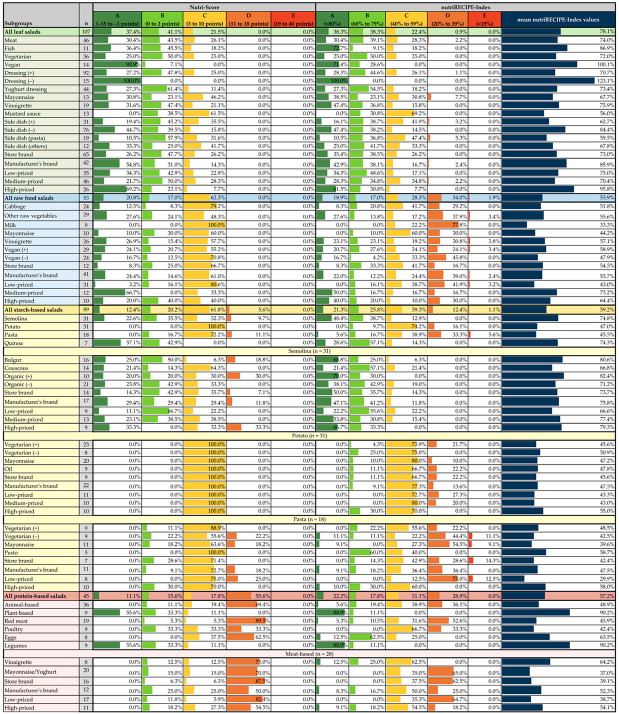

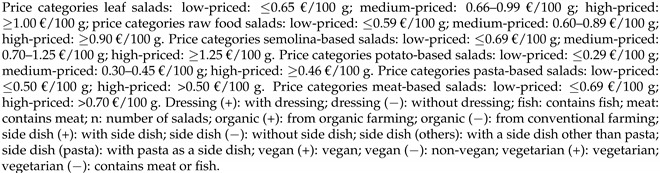
Comparison of the mean nutriRECIPE-Index values with the respective Nutri-Score ratings.

The mean nutriRECIPE-Index of leaf salads is 78.1%, with each 38.3% classified as A and B, 22.4.1% as C, 0.9% as D, and none as E (Figure 2). In the Nutri-Score, 37.4% of leaf salads were assigned to A, 41.1% to B, 21.5% to C, and none to D or E. Subgroup-specifically, all leaf salads without dressing (dressing (−)) reach an A, whereas 92.9% of vegan (+), and 69.2% of high-priced leaf salads reach an A. For the nutriRECIPE-Index, these three subgroups also achieve the best ratings with arithmetic means of 123.1%, 100.1%, and 95.8%, respectively. Unlike the Nutri-Score, most salads (72.7%) with fish receive an A in the nutriRECIPE-Index, which is thus a larger share than in vegan (+). Given that most salads with fish achieve nutriRECIPE-Index values just above 80%, the mean for fish is 86.9%. The high nutriRECIPE-Index values are mainly due to the favorable scores for iron, iodine, folate, and vitamin D, which are not considered for the calculation of the Nutri-Score. With the nutriRECIPE-Index, vegan (+) has 21.5% fewer salads in A than Nutri-Score. However, with the Nutri-Score, salads in vegan (+) mostly have points between −4 and 2, around the border of A and B (Supplementary Table S3), so the rating by both systems looks different at first glance, but very similar on closer inspection. No salad with mustard sauce was assigned to A with both systems. With the Nutri-Score, 38.5% are in B and 61.5% in C. This is echoed in the nutriRECIPE-Index with 30.8% in B and 69.2% in C and a mean of 56.0%, the lowest value within leaf salads. Both rating systems show a minor scatter of the individual salads within the mustard sauce group. With the Nutri-Score, the salads assigned to B mainly had 1 or 2, while they mainly achieved 3 or 4 points within C.

#### 3.2.2. Raw Food Salads

As with leaf salads, no raw food salad recorded was assigned to Nutri-Score D or E. In contrast to leaf salads, 62.3% were rated C. The remaining raw food salads were distributed almost evenly between A and B. These differences are even more revealed in the nutriRECIPE-Index (A: 18.9%, B: 17.0%, C: 28.3%, D: 34.0%, E: 1.9%; mean value: 53.3%). The contents of energy, SFA, sugar, protein, and salt were assessed by both labels and have a negative impact on the rating. Since the Nutri-Score evaluates the fruit content overall, high scores are achieved here. In the nutriRECIPE-Index, the individual nutrient amounts are evaluated so that positive values are achieved for vitamin C, vitamin B_6_, folate, and vitamin E, but these cannot compensate for the unfavorable rating of the other nutrients due to the weighting factors. The nutriRECIPE-Index ratings of the raw food salads indicate much higher nutritional differences among the salads than the Nutri-Score. There are differences in salads that are not rated A or B. While no salad is rated worse than C with the Nutri-Score, 35.9% of salads are rated D or E with the nutriRECIPE-Index. The nutriRECIPE-Index mean values of the subgroups range from 33.3% (milk dressing) to 73.2% (medium-priced). In medium-priced, 66.7% are in A, none in B, and 33.3% in C. Most of the salads in C are close to the B-border. Exceptions are some salads, which reach between −5 and −8 Nutri-Score-points. With the nutriRECIPE-Index, the medium-priced salads are classified into four different categories (A–D). The ratings range from 27.1% to 113.8%. Since most of the salads in A achieve excellent ratings, the nutriRECIPE-Index mean value is 73.2%, which is the best rating for a subgroup within the raw food salads.

#### 3.2.3. Starch-Based Salads

12.4% of the starch-based salads were rated with a Nutri-Score A, 20.2% B, 61.8% C, and 5.6% D. Although the Nutri-Score evaluation is less favorable for starch-based salads than for raw food salads, the nutriRECIPE-Index mean value is slightly better at 59.2% (A: 21.3%, B: 25.8%, C: 39.3%, D: 12.4%, E: 1.1%). Differentiating starch-based salads according to subsets reveals that quinoa (A: 57.1%, B: 42.9%), achieves a much better Nutri-Score rating than semolina (A: 22.6%, B: 35.5%, C: 32.3%, D: 9.7%). The nutriRECIPE-Index ratings for quinoa- and semolina-based salads hardly differ with 74.3% and 74.8%, respectively. The raw food salads show particularly high fruit contents, whereas starch-based salads receive the worst ratings. All semolina-based salads are below the 40.0% threshold for fruit content and therefore achieve zero, while most quinoa-based salads achieve one to two points better in this regard. Many semolina-based salads are at the B threshold, partly explaining the difference between these two subgroups. With 9.7% in B, 74.2% in C, and 16.1% in D, small differences within the potato-based salads are apparent in the nutriRECIPE-Index assessment. Although the Nutri-Score points range from 3 to 10, all salads within the potato group were assigned a Nutri-Score C. This is similar within the pasta-based salads. While Nutri-Score ratings between B and D are achieved, all five categories are coverd by the nutriRECIPE-Index.

#### 3.2.4. Protein-Based Salads

With the Nutri-Score, 11.1% of protein-based salads were rated A, while 15.6% received B, 17.8% C, and 55.6% D. The A-ratings were exclusive to legume-based salads. The protein-based salads have the most unfavorable Nutri-Score rating of all main salad groups. The corresponding nutriRECIPE-Index is more favorable with a mean value of 57.2% (A: 22.2%, B: 17.8%, C: 31.1%, D: 28.9%). This is particularly apparent with red meat- and egg-based salads. With the Nutri-Score, red meat-based salads were rated with 5.3% in B and C and 89.5% in D, whereas with the nutriRECIPE-Index, they performed much better (A: 5.3%, B: 10.5%, C: 31.6%, D: 52.6%). Red meat contains essential nutrients such as iron, vitamin B_12,_ and zinc, which are only considered in the nutriRECIPE-Index (Supplementary Table S1). The nutriRECIPE-Index allows a more comprehensive evaluation, given that 19 nutrients are included with additional weighting. Consequently, egg-based salads, with a mean nutriRECIPE-Index of 63.5% (A: 12.5%, B: 62.5%, C: 25.0%), perform much better than in the Nutri-Score (C: 37.5%, D: 62.5%). This is due to the vitamin D content, which has the highest weighting factor at 3.00, as well as the high iodine, vitamin B_12,_ and vitamin E content. Poultry-based salads, with 33.3% each in B, C, and D, appear to be significantly better with the Nutri-Score than red meat-based salads with 5.3% each in B and C and 89.5% in D. In contrast, the mean nutriRECIPE-Index of poultry-based salads is slightly lower at 42.4%. Poultry-based salads achieve more favorable scores for vitamin E, iron, and iodine, whereas red meat-based salads score better for thiamine, riboflavin, vitamin B_6_, folate, and vitamin B_12_, as well as calcium, magnesium, and zinc (Supplementary Table S1). Here, too, the fact comes into play that the content of micronutrients is only indirectly included in the Nutri-Score if they are contained in the product in the form of fruits, vegetables, legumes, oil (walnut, canola, olive), or nuts. However, a distinction between specific species—reflecting different micronutrient contents—is not made.

#### 3.3. Comparison of the Main Groups by Big7 and nutriRECIPE-Index

**Figure 3:**
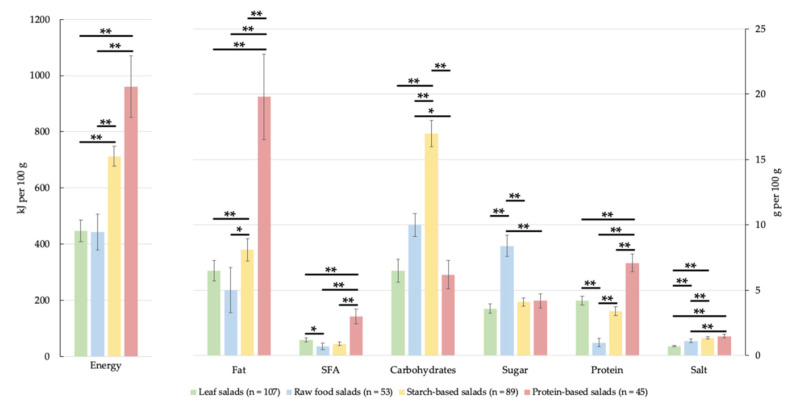
Comparison of the median Big7 values of the main groups. Plot of all Big7 medians in kJ/g per 100 g for each main group. Shows the significant differences in energy [kJ per 100 g] and total fat, SFA, total carbohydrates, sugar, protein, and salt content [g per 100 g] between the main groups according to Kruskal-Wallis test plus pairwise comparisons with Bonferroni correction. Error bar shows 95%-CI; *: *p* < 0.05; **: *p* < 0.01; n, number of salads; SFA, saturated fatty acids.

107 leaf salads, 53 raw food salads, 89 starch-based salads, and 45 protein-based salads were compared in the Big7-nutrients (packaging information) and the nutriRECIPE-Index (aggregated approach). The data are neither normally distributed nor homogeneous in variance. Therefore, they were presented using the median (Figure 3 and Table 2). All following numbers refer to 100 g salad. While the medians of energy contents for leaf salads and raw food salads are similar at 447 kJ and 443 kJ, highly significant greater median energy contents of 713 kJ and 961 kJ were calculated for starch-based salads and protein-based salads, respectively. At 19.8 g, the protein-based salads contain a highly significant greater fat content than the other main groups, with 5.0 g for raw food salads, 6.7 g for leaf salads, and 8.1 g for starch-based salads. The 95%-CI ranges from 17.0 g to 23.0 g, so there is a high scatter within the protein-based salads. The SFA of the protein-based salads is also highly significantly greater than the other main groups at 3.0 g. The medians of leaf salads, raw food salads, and starch-based salads range between 0.7 g and 1.2 g. Therefore, the fat contained within the salads consists mainly of unsaturated fatty acids. When comparing carbohydrates, protein-based salads with 6.2 g are just below leaf salads with 6.5 g and thus have the lowest content of all main groups. The starch-based salads have 17.0 g carbohydrates, far above protein-based salads and leaf salads, but also above raw food salads with 10.0 g. Raw food salads have an 8.4 g sugar content, twice as much as the other main groups. However, when comparing protein, raw food salads are with 1.0 g highly significant below the values of all other main groups. In leaf salads and starch-based salads, the protein contents are 4.2 g and 3.4 g, and the values of protein-based salads are the highest at 7.1 g. Except for starch-based salads to protein-based salads, the salt contents of the main groups show highly significant differences. While leaf salads with 0.7 g are below all other main groups, raw food salads and starch-based salads reach 1.1 g and 1.4 g, respectively. Protein-based salads have a salt content of 1.5 g, twice as much as leaf salads. Leaf salads achieve the highest nutriRECIPE-Index with 75.1%. Starch-based and protein-based salads follow them with 55.1% and 54.2%, respectively. With a nutriRECIPE-Index of 48.1%, raw food salads range lowest. When looking at nutriRECIPE-Index scores (Table 2), it becomes evident that each main group has strengths and weaknesses. The nutriRECIPE-Index scores, apart from iodine, vitamin B_12_, D, and E, are very heterogeneous between the main groups, although the median total initially reflects this only slightly. The protein-based salads have comparatively high fat contents in the weight-based analysis. Although the SFA content is twice as high as in leaf salads, they perform only 0.2 worse in the energy-related consideration. However, the nutriRECIPE-Index-score of 0.1 in protein-based salads is much lower than the other main groups, with 0.1 for leaf salads and 0.6 and 0.7 for raw food salads and starch-based salads, respectively. Nevertheless, the unsaturated fatty acids content within protein-based salads proves to be the most advantageous, with a nutriRECIPE-Index-score of 1.8, while the other main groups range between 1.4 and 1.5. The nutriRECIPE model differentiates carbohydrates by sugar and fiber. For both nutrients, there is a heterogeneous distribution between the main groups. The nutriRECIPE-Index scores for sugar are 0.4 for protein-based salads, 0.0 for starch-based salads, −0.4 for leaf salads, and −1.3 for raw food salads. Contrary to leaf salads and raw food salads, which reach nutriRECIPE-Index scores of 1.0 and 1.3, respectively, starch-based salads with 0.9 and protein-based salads with 0.0 are below the intake recommendation for fiber. Although protein-based salads have the highest protein content per 100 g, they only achieve the second-highest score with 0.7 in the energy-related analysis. The highest score was achieved by leaf salads, which at 0.8 is 0.1 higher than protein-based salads. Starch-based salads and raw food salads are far below with 0.2 and 0.0. Analogous to the protein content, the ratio of the salads in terms of salt concentration shifts between the main groups in the energy-related observation. The values are −0.7 for leaf and protein-based salads, −0.9 for starch-based salads, and −1.0 for raw food salads. As the nutriRECIPE-Index aims for a nutrient ratio that is as balanced as possible [13], leaf salads achieve the highest nutriRECIPE-Index values due to moderately high scores for many nutrients. However, in addition to sugar and salt, which have a negative effect on the value at −0.4 and −0.7, iodine and vitamin D with 0.1 and 0.0, respectively, are also worth mentioning. The highest weighting factors are assigned to iodine and vitamin D [13]. Here, the other main groups also achieve only a score of 0.0. The score for iron and calcium, each with 1.1, and folate with 1.7 are particularly outstanding. There is a less balanced nutrient distribution in raw food salads than within leaf salads. Most of the scores receive the worst or second-worst value. Although raw food salads achieve higher scores in vitamin C (1.9) and vitamin E (1.8), strongly negative scores are also achieved with 1.0 for salt and 1.3 for sugar. The last score represents a clear difference from the other main groups. Finally, raw food salads achieve the lowest nutriRECIPE-Index after including the weighting factors. In starch-based salads, there is a heterogeneous nutrient distribution overall. While some of the scores are up to 0.2, the other and rather larger part reaches scores of 0.8 to 1.5. The highest score is reached for unsaturated fatty acids. The score of 1.1 for iron is also comparatively high. Only the score for salt with −1.1 leads to a clear deduction. Although the nutriRECIPE-Index for starch-based and protein-based salads is similar, there are clear differences within the individual nutrients. While starch-based salads achieve a 0.7 for SFA, the score for protein-based salads is −0.1. Higher scores of 1.0 are also found for starch-based salads for fiber and vitamin C, whereas protein-based salads achieve values of 0.0 and 0.1, respectively. In contrast, starch-based salads score only 0.2 for protein and 0.1 for sugar, while protein-based salads score 0.8 and 0.7, respectively. Another difference is vitamin B_12,_ with 0.0 for starch-based salads and 0.5 for protein-based salads. Finally, both main groups were given similar nutriRECIPE-Index values after adding the weighting factors.

#### 3.4. Comparison of nutriRECIPE-Index Values Depending on Approaches

**Figure 4:**
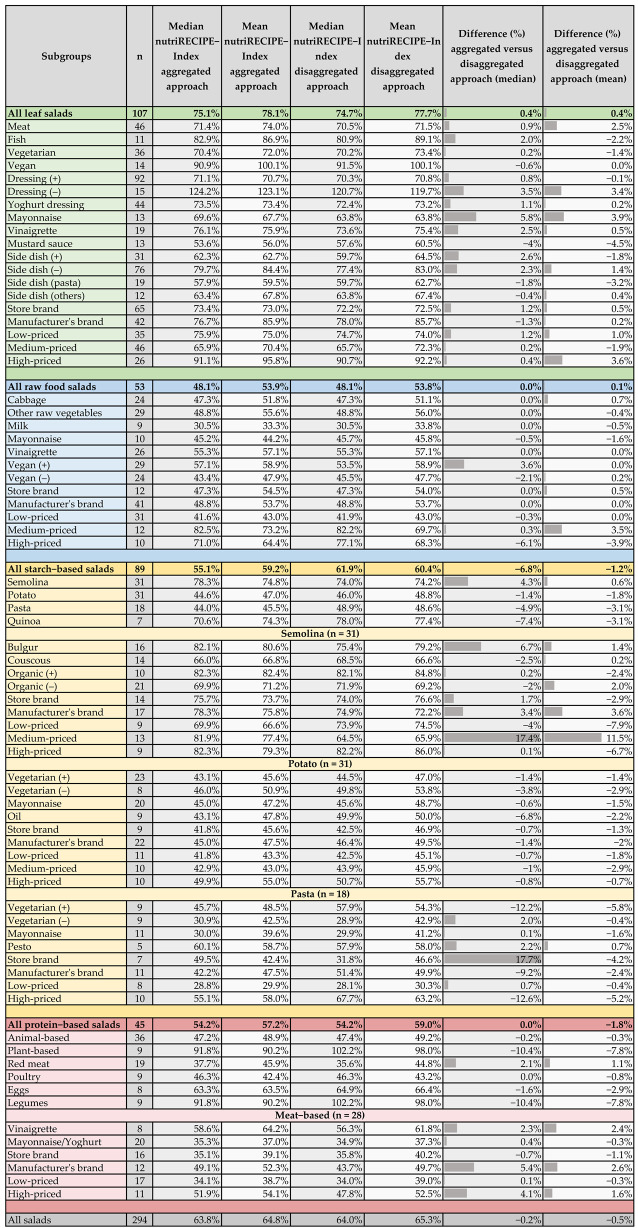

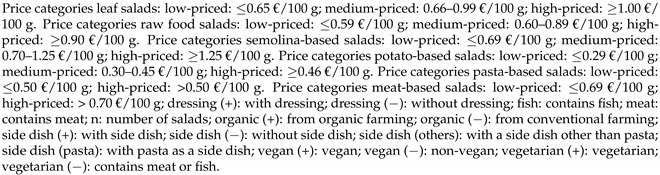
Comparison of median and mean nutriRECIPE-Index values depending on the disaggregated or aggregated approach.

Overall, when comparing the different ingredient recording approaches, the aggregated approach leads to slightly lower nutriRECIPE-Index values than the disaggregated approach (median: −0.2%, mean: −0.5%) (Figure 4). However, the differences do not follow a uniform pattern within single salad groups, leading to varying maximal differences of −12.6% (high-priced pasta-based salads) and +17.7% (private label pasta-based salads). Within the leaf salads, the differences are minor and less than 5.8%. Within the raw food salads, the medians and means of the main group differ even less between the approaches, except for the high-priced salads (median: −6.1%, mean: −3.9%). Minor differences can be seen only when looking at the individual subgroups, although no clear trend can be identified here. Generally, starch-based and protein-based salads show higher variance than the other main groups. Here, the amount of processed ingredients is much higher than in leaf and raw food salads. In leaf and raw food salads, raw vegetables are the base, complemented by dressing and pieces of meat/eggs/cheese. While a quarter of leaf salads contain a starch-based side dish that has undergone a cooking process, no raw food salads contain a side dish. Except for a few ingredients (e.g., cheese, mayonnaise, or iodized salt), the raw food salads contain few aggregated ingredients, which is why there are minor differences between the approaches. Given that starch-based and protein-based salads are based on highly processed foods, supplemented by dressing, meat/eggs/cheese, the impact of the different approaches is larger.

## 4. Discussion

In this study, 294 ready-to-eat salads in German supermarkets were surveyed and assessed for their nutritional value. Overall, leaf salads achieved the best ratings. Furthermore, individual subgroups were identified within all main groups that stood out due to their beneficial nutritional composition. Although salads are often considered the epitome of a healthy lifestyle, a large proportion of them achieved nutriRECIPE-Index values below 60.0%. Even within leaf salads, only occasional values above 100.0% were achieved. Compared with traditional cafeteria meals in a previous study [36], ready-to-eat salads performed in the same range. Finally, due to the heterogeneity shown, whether salads are to be assessed as generally healthy cannot be conclusively stated.

### 4.1. Non-Continuous (Level System) Versus Continuous Scale

Studies show that the non-continuous Nutri-Score has sufficient ability to discriminate the nutritional quality of products within main food groups and subgroups, as assessed by the number of colors available in each group. The discrimination performance was considered satisfactory if at least three classes of Nutri-Score were present in the food group [37,38]. However, our data shows that the nutriRECIPE-Index discriminates as well or even better in 63 out of 73 subgroups. This was particularly evident in groups where very similar products were compared, such as potato salads (Nutri-Score: C only, nutriRECIPE-Index: B, C and D). In the Nutri-Score, a better nutrient distribution is expressed with lower total points, subsequently assigned to one of the five categories [14]. The level sizes of categories A to E differ. While B comprises only values 0 to 2 and is thus a narrow transition between an excellent rating by A to the mediocre rating by C, C comprises values from 3 to 10 [14]. Consumers could assume an even-level classification, possibly leading to confusion or misinformation. Due to the level system used for presentation and scoring, even minor differences in the composition of the salads can be decisive for the classification. Nutritionally, these changes have insignificant effects; despite this, a differentiated evaluation occurs. In contrast, a major change may not be represented in the rating. Thus, there may be an overestimation or underestimation of the difference between foods. Studies have shown that 15% of the products in the Slovenian food supply are on the borderline of the next better Nutri-Score rating [38]. Similar distributions were observed in other European countries [39,40,41]. Within the nutriRECIPE-Index, the presentation and rating calculation are based on a continuous scale, so differences between salads can be represented in the corresponding proportion without categorical distortion. Due to the broad scale of the nutriRECIPE-Index value from 0.0% to 300.0% [13], both very favorable and very unfavorable formulations can be shown. However, with the Nutri-Score, no differentiation is possible above B and below D [14], so particularly healthy salads cannot be highlighted. In a Dutch study, scenario analyses showed that reducing the sodium, saturated fat or sugar content by one Nutri-Score point resulted in certain products achieving a more favorable Nutri-Score (between 0 and 30%) [42]. Due to the continuous scale of the nutriRECIPE-Index and the corresponding mapping of a changed nutritional value, the incentive for reformulation by manufacturers could be even stronger.

### 4.2. Selection of Nutrients

In the nutriRECIPE-Index, the evaluation considers 19 nutrients selected depending on public health concerns and according to official recommendations. Here, the micronutrient and macronutrient amounts are included in the assessment but not phytochemicals [13]. In the Nutri-Score, on the other hand, food is evaluated based on seven parameters, whereby micronutrients and phytochemicals are not directly assessed. Instead, the total health effects of fruits, vegetables, legumes, nuts, and oils (olive, walnut, canola), are summarized into a single parameter to be included in the assessment [14]. For example, due to their typical formulation [10], meat-based salads contain high levels of salt, fat, and therefore energy and hardly any fiber. However, these are mainly found in vegetables and whole grains [32], which are only permitted in limited quantities due to legal regulations [10]. Thus, they principally achieve an unfavorable rating in four out of seven parameters of the Nutri-Score. The remaining attributes cannot effectively compensate for this due to the maximum number of points per parameter. Meat salads contain essential nutrients such as iron, vitamin B_12,_ and zinc, but this is only considered in the nutriRECIPE-Index. This illustrates that micronutrients are only included in the Nutri-Score if they are contained in the product in the form of fruits, vegetables, legumes, oil (canola, walnut, olive), or nuts. Raw food salads have a high content of these components, whereas starch-based salads receive the lowest rating. This can make a difference of up to five Nutri-Score points in the calculation. Furthermore, the fruit content only positively affects the rating once the 40.0% mark has been exceeded [14]. Given that many of the starch-based salads rated with a C only narrowly missed allocation to B, this could have had a decisive effect. The Nutri-Score does not distinguish between fruit and vegetables or their ratio to each other. Only their combined total is considered [14]. In contrast, the nutriRECIPE-Index evaluates a balanced ratio of numerous nutrients more favorably [13]. For example, salad No. 67, a carrot salad, receives −6, one of the best Nutri-Score ratings. However, due to the low diversity of micronutrients of the fruit content, it only achieves a mediocre nutriRECIPE-Index rating of 64.0% (Supplementary Table S3).

The Nutri-Score was designed to differentiate goods within a product group [14]. However, because of the small number of variables, these differences can only be reflected to a limited extent, especially when the food consists of one or a few ingredients, such as oils, spreadable fats, or juices. For this reason, the calculations for oils and cheese, and beverages have been adjusted [14]. As a result, not all product groups are comparable anymore. The calculation proves to be inflexible given that new limits and criteria for scoring must be defined. These changes must ultimately be implemented by the companies, for which they must be given appropriate time. In contrast, the nutriRECIPE-Index is applicable to all product groups due to its conceptualization and does not require any further adjustments.

The Nutri-Score only assesses the amount of SFA when considering the fat content [14], whereas the nutriRECIPE-Index evaluates the fatty acid composition regarding SFA, MUFA, and PUFA [13]. While leaf salads with mustard sauce receive an unfavorable rating in the Nutri-Score due to their high amount of SFA, the high proportion of unsaturated fatty acids ensures that the overall fat content is rated as positive within the nutriRECIPE-Index. Nevertheless, high fat content negatively impacts the rating in both scoring systems because of the high energy content.

### 4.3. Weight-Adjusted Approach (Nutri-Score) Versus Energy-Adjusted Nutrient Density Approach (nutriRECIPE-Index)

A fundamental difference between the two scoring systems is that the Nutri-Score assesses nutrient concentrations based on the amount per 100 g or 100 mL [14], whereas the nutriRECIPE-Index uses the energy-adjusted nutrient density to evaluate foods [13]. As highlighted in the underlying formulas (Section 2.4) the multiplied terms N_product_/N_rec_ and E_rec_/E_product_, on the one side, set the ratio of nutrients served in one kJ in proportion and thus represent the measure of energy-adjusted nutrient loading. On the other side, these two conjoint terms level out the relevance of the reference unit (here 100 g), as both the nutrient and the energy term refer to the same unit. In summary, a high (or low) energy content has a greater effect on the final nutriRECIPE-Index (compared to the Nutri-Score) because it leads to lower nutrient density and, thus, a lower rating for all nutrients and, ultimately, for the entire food [13] Within the evaluation framework of the Nutri-Score, the energy content constitutes only one of the seven criteria. Thus, a high energy content also impacts the score negatively, but only by rating the high energy content itself [14]. Moreover, the energy-related term E_rec_/E_product_ leads in conjunction with the following term N_act_/N_rec_ to an objectifiable and public health based-weighting of the single nutrients considered in the nutriRECIPE-Index (for details see next section). In case of the Nutri-Score all ingoing parameters are weighted equally.

### 4.4. Adjustment of the nutriRECIPE-Index Based on Physiological Requirements

In the nutriRECIPE-Index, the values refer to the nutrient requirements of an adult aged 19 to 65 years with an energy requirement of 8370 kJ daily. The degree of fulfillment of the nutrients is calculated utilizing a logarithmic function to reflect the actual effect better. Given that each nutrient can have beneficial and adverse effects, the one-dimensional classification of a nutrient as having a beneficial or adverse function is considered insufficient [13]. Therefore, a distinction between moderation and adequacy components is included in calculating the nutriRECIPE-Index. With additional consideration of current recommendations and dietary patterns, minimum and maximum target values are calculated for all nutrients included. Moreover, the supply situation is included in the calculation [13]. Furthermore, by adjusting the weighting factors, the nutriRECIPE-Index can be adapted to changes in supply. The weighting is currently based on the survey data of the German National Nutrition Survey II of the period 2005 to 2007, in which almost 20,000 people aged 14 to 80 years were interviewed throughout Germany about their food consumption and dietary habits [43]. However, it is possible that nutrient supply has changed in recent years and that the data are therefore outdated. Moreover, the calculations currently only refer to one group of people and represent the consumption of an average person living in Germany [13].

### 4.5. Effectiveness of Front-of-Pack Nutrition Labeling

Studies suggest that the perception and understanding are key elements in the effectiveness of front-of-pack labels. Labels with color highlighting are perceived much better [44]. Interpretive labels are easier to understand and more noticeable than reductive labels [45]. Compared with three other models against which the Nutri-Score prevailed, the majority of consumers surveyed rated the logo as intuitive and easy to understand [46]. This may be related to the color coding of the Nutri-Score from green to dark orange, which increases consumer attention [47,48] and helps individuals to process information by using universal symbolic colors [49]. Therefore, labeling that combines both summary and color-coded characteristics, such as the Nutri-Score and nutriRECIPE-Index, would be associated with better comprehension by consumers [50,51,52]. Thus, the nutriRECIPE-Index could be also an easily understandable alternative.

### 4.6. Limitations

Despite the advantages of the nutriRECIPE-Index, which arise from the automated and universally applicable data processing framework, there are the following potential sources of errors. Within the recipe calculation of the nutriRECIPE-Index, there are many theoretical solutions for the equation system. Even if the square error is 0.0, it cannot be guaranteed that the calculated ingredient proportions correspond to the actual amounts in the product. The calculation is made even more difficult if synonyms of ingredients are given, or the Big7 information does not accurately reflect the average nutritional value. Thus, even minor discrepancies can lead to distorted solutions of the equation system. Despite a successful improvement of the ingredient recording variant, the error susceptibility of the calculation could not be eliminated due to measurement inaccuracies and natural fluctuations. Since the Nutri-Score is usually calculated by the manufacturers themselves, they have access to all relevant ingredient proportions and nutrient amounts [14]. Nevertheless, as outlined in Section 3.4, even between the different approaches, the nutriRECIPE-Index values differ only slightly, and the variances were in all salad groups lower than 20%. In the context of the specifications of the mandatory European regulation (EU) No. 1169/2011, which also allows a tolerance of up to 20% for individual nutrients [21], these deviations seem acceptable. These inaccuracies of the Big7 can be caused by variations in the product, the disregard of the degree of processing of individual ingredients, or the nonexistence of these in databases. Most of the salads recorded contain processed foods such as cheese. The degree of processing of the ingredients has a decisive influence on the food’s nutrient composition. For instance, lactose is eliminated during cheese production [53], but the algorithm cannot consider this if only the sub-ingredients are included. This indicates that the aggregated approach is preferable because it adequately reflects the actual food composition. Therefore, it must be ensured that both the manufacturer’s Big7 calculation and the calculation of ingredient proportions by the algorithm are based on the nutritional composition of the final product, not on the unprocessed ingredients it contains. Another potential source of error could be if the manufacturer’s calculation is based on literature data unequal to the GND. Therefore, all manufacturers of the recorded salads were contacted to find out which data they used to determine the Big7. The evaluation revealed that the manufacturers of 92 out of 294 salads calculate the nutritional values using literature data, which were verified by random analyses. The manufacturers of 46 salads indicated that the nutritional values were determined using a mixed form of analytical and literature data. In the case of 20 salads, only literature data were used, while only analytical data were used in the case of 51 salads. No information was provided by the manufacturers of the remaining 85 salads. In total, only two manufacturers (30 salads) reported using literature data unequal to the GND (Supplementary Table S2). The problem with the discrepant database could therefore be negligible. Regarding the ingredient quantity calculation, the inclusion of the companies could be beneficial. For example, communicating how the Big7 were recorded or which database was used could result in more precise solutions in the equation system. Preferably, the specific ingredient proportions in the product would be communicated. A program could be created to overcome the justified objection of trade secrecy that enables encrypted transmission of calculated nutritional information after entering the ingredient proportions. Manufacturers could select the applicable ingredients from a list and describe them in percentages in this program. This would ensure uniform ingredient naming and enable automatic linking with the GND.

## 5. Conclusions

Due to the heterogeneity shown, it cannot be conclusively assessed whether salads could be rated as generally healthy. A case-by-case approach is therefore required. The Nutri-Score and nutriRECIPE-Index proved appropriate tools to reflect the nutritional differences adequately. While the Nutri-Score is calculated by the manufactures, the nutriRECIPE-Index is calculated independently. One of the core differences between both approaches lies in the non-continuous level system of the Nutri-Score, in contrast to the continuous scale of the nutriRECIPE-Index. Additionally, differences are found in the selection and the influence of specific nutrients included in the calculation. Whereas the Nutri-Score takes seven parameters into account, the nutriRECIPE-Index considers 19 nutritionally relevant variables. Further, the Nutri-Score evaluates the nutrient concentration in relation to weight, whereas the nutriRECIPE-Index relates it to the energy content of the food. Besides this, the supply and nutritional demand of the target group are included in the nutriRECIPE-Index. Hence, due to the more comprehensive and differentiated assessment, the nutriRECIPE-Index is suitable for representing even marginal differences accordingly, whereas the Nutri-Score is more suitable for a general orientation.

## Figures and Tables

**Table 1:** Classification of the recorded salads (n = 294) into main groups and subgroups.

Main Group	Classification Criteria		Subgroup
Leaf salads n = 107	Diet, n = 107		Meat, n = 46
			Fish, n = 11
			Vegetarian (+), n = 36
			Vegan (+), n = 14
	Presence of dressing, n = 107		Dressing (+), n = 92
			Dressing (−), n = 15
	Dressing base, n = 89		Yogurt dressing, n = 44
			Mayonnaise, n = 13
			Vinaigrette, n = 19
			Mustard sauce, n = 13
	Presence of side dish, n = 107		Side dish (+), n = 31
			Side dish (−), n = 76
	Type of side dish, n = 31		Side dish (pasta), n = 19
			Side dish (others), n = 12
	Brand, n = 107		Private label, n = 65
			Branded label, n = 42
	Price range, n = 107		Low-priced (≤0.65 €/100 g), n = 35
			Medium-priced (0.66–0.99 €/100 g), n = 46
			High-priced (≥1.00 €/100 g), n = 26
Raw food salads n = 53	Salad base, n = 53		Cabbage, n = 24
			Other raw vegetables, n = 29
	Dressing base, n = 45		Milk dressing, n = 9
			Mayonnaise, n = 10
			Vinaigrette, n = 26
	Diet, n = 53		Vegan (+), n = 29
			Vegan (−), n = 24
	Brand, n = 53		Private label, n = 12
			Branded label, n = 41
	Price range, n = 53		Low-priced (≤0.59 €/100 g), n = 31
			Medium-priced (0.60–0.89 €/100 g), n = 12
			High-priced (≥0.90 €/100 g), n = 10
Starch-based salads n = 89	Semolina n = 31	Salad base, n = 30	Bulgur, n = 16
			Couscous, n = 14
		Cultivation, n = 31	Organic (+), n = 10
			Organic (−), n = 21
		Brand, n = 31	Private label, n = 14
			Branded label, n = 17
		Price range, n = 31	Low-priced (≤0.69 €/100 g), n = 9
			Medium-priced (0.70–1.25 €/100 g), n = 13
			High-priced (≥1.26 €/100 g,) n = 9
	Potato n = 31	Diet, n = 31	Vegetarian (+), n = 23
			Vegetarian (−), n = 8
		Dressing base, n = 29	Mayonnaise, n = 20
			Oil, n = 9
		Brand, n = 31	Private label, n = 9
			Branded label, n = 22
		Price range, n = 31	Low-priced (≤0.29 €/100 g), n = 11
			Medium-priced (0.30–0.45 €/100 g), n = 10
			High-priced (≥0.46 €/100 g), n = 10
	Pasta n = 18	Diet, n = 18	Vegetarian (+), n = 9
			Vegetarian (−), n = 9
		Dressing base, n = 16	Mayonnaise, n = 11
			Pesto, n = 5
		Brand, n = 18	Private label, n = 7
			Branded label, n = 11
		Price range, n = 18	Low-priced (≤0.50 €/100 g), n = 8
			High-priced (>0.50 €/100 g), n = 10
	Quinoa, n = 7		
Protein-based salads n = 45	Source of protein, n = 45		Animal-based, n = 36
			Plant-based, n = 9
	Salad base, n = 45		Red meat, n = 19
			Poultry, n = 9
			Eggs, n = 8
			Legumes, n = 9
	Meat- based n = 28	Dressing base, n = 28	Vinaigrette, n = 8
			Mayonnaise/Yogurt, n = 20
		Brand, n = 28	Private label, n = 16
			Branded label, n = 12
		Price range, n = 28	Low-priced (≤0.69 €/100 g) n = 17
			High-priced (>0.70 €/100 g) n = 11

Dressing (+): with dressing; dressing (−): without dressing; fish: contains fish; meat: contains meat; n: number of salads; organic (+): from organic farming; organic (−): from conventional farming; side dish (+): with side dish; side dish (−): without side dish; side dish (others): with a side dish other than pasta; side dish (pasta): with pasta as a side dish; vegan (+): vegan; vegan (−): non-vegan; vegetarian (+): vegetarian; vegetarian (−): contains meat or fish. Subgroup comparisons were performed only when the sample size was large enough.

**Table 2:** Comparison of the median nutriRECIPE-Index-scores and total nutriRECIPE-Index values of the main groups.

Weighting Factors		1.00	1.00	1.84	1.17	1.07	1.24	1.95	0.85	1.09	0.92	0.93	0.77	0.72	0.65	0.96	0.74	0.72	3.00	0.95	
	Nutrients	SFA	MUFA + PUFA	Sugar	Fiber	Protein	Salt	Iodine	Magnesium	Iron	Zinc	Calcium	Thiamine	Riboflavin	Vitamin B_6_	Folate	Vitamin B_12_	Vitamin C	Vitamin D	Vitamin E	nutriRECIPE-Index
Main Groups																					
Leaf salads		0.1	1.5	−0.4	1.0	0.8	−0.7	0.1	1.0	1.1	1.0	1.1	0.9	0.8	1.1	1.7	0.3	1.4	0.0	1.6	75.1%
Raw food salads		0.6	1.4	−1.3	1.3	0.0	−1.0	0.0	0.8	0.6	0.2	0.5	0.4	0.3	1.4	1.3	0.0	1.9	0.0	1.8	48.1%
Starch- based salads		0.7	1.5	0.0	0.9	0.2	−0.9	0.0	1.0	1.0	0.7	0.0	0.8	0.1	1.1	0.7	0.0	1.4	0.0	1.4	55.1%
Protein- based salads		−0.1	1.8	0.4	0.0	0.7	−0.7	0.0	0.2	0.5	0.6	0.0	0.9	0.8	0.8	0.1	0.5	0.1	0.0	1.6	54.2%

MUFA: monounsaturated fatty acids; PUFA: polyunsaturated fatty acids; SFA: saturated fatty acids.

## Data Availability

All relevant background data is availbale in the Supplementary Material.

## Supplementary Materials

The following supporting information can be downloaded at: https://www.mdpi.com/article/10.3390/foods11244011/s1, Figure S1: Decision tree for determining the appropriate test; Table S1: Recorded salads with ingredient lists, Big7 and calculated nutrient contents; Table S2: Manufacturer’s responses regarding the determination methods; Table S3: Filled template of the Belgian Federal Public Service with calculated Nutri Scores and corresponding nutriRECIPE-Index values with both approaches; Tables S4–S34: Comparison of nutriRECIPE-Index and nutrition content per 100 g among the groups.
