# Supplementary material for: Nutritional Assessment of Ready-to-Eat Salads in German Supermarkets: Comparison of the nutriRECIPE-Index and the Nutri-Score
Source: Foods. 2022 Dec 11;11(24):4011. doi: 10.3390/foods11244011 (PMC9777885; doi:10.3390/foods11244011)
Supplement: Supplementary file 1 [file foods-11-04011-s001.zip › foods-1995016-supplementary.pdf]

Supplemental Tables

Table S1. Recorded salads with ingredient lists, Big7 and calculated nutrient contents.
Table S2. Manufacturer's responses regarding the determination methods.
Table S3. Filled template of the Belgian Federal Public Service with calculated Nutri-Scores and corresponding nutriRECIPE-Index values with both approaches.
Table S4. Comparison of nutriRECIPE-Index and nutrition content per 100 g among main groups (n = 294).
Table S5. Comparison of nutriRECIPE-Index and nutrition content per 100 g according to diets within leaf salads (n = 107).
Table S6. Comparison of nutriRECIPE-Index and nutrition content per 100 g according to presence of a dressing within leaf salads (n = 107).
Table S7. Comparison of nutriRECIPE-Index and nutrition content per 100 g according to dressing base within leaf salads (n = 107).
Table S8. Comparison of nutriRECIPE-Index and nutrition content per 100 g according to presence of a side dish within leaf salads (n = 107).
Table S9. Comparison of nutriRECIPE-Index and nutrition content per 100 g according to the type of side dish within leaf salads (n = 107).
Table S10. Comparison of nutriRECIPE-Index and nutrition content per 100 g according to the brand within leaf salads (n = 107).
Table S11. Comparison of nutriRECIPE-Index and nutrition content per 100 g according to the price range within leaf salads (n = 107).
Table S12. Comparison of nutriRECIPE-Index and nutrition content per 100 g according to the salad base within raw food salads (n = 53).
Table S13. Comparison of nutriRECIPE-Index and nutrition content per 100 g according to the dressing base within raw food salads (n = 53).
Table S14. Comparison of nutriRECIPE-Index and nutrition content per 100 g according to the diet within raw food salads (n = 53).
Table S15. Comparison of nutriRECIPE-Index and nutrition content per 100 g according to the brand within raw food salads (n = 53).
Table S16. Comparison of nutriRECIPE-Index and nutrition content per 100 g according to the price range within raw food salads (n = 53).
Table S17. Comparison of nutriRECIPE-Index and nutrition content per 100 g according to the salad base within starch-based salads (n = 89).
Table S18. Comparison of nutriRECIPE-Index and nutrition content per 100 g according to the salad base within semolina salads (n = 31).
Table S19. Comparison of nutriRECIPE-Index and nutrition content per 100 g according to the cultivation method within semolina salads (n = 31).
Table S20. Comparison of nutriRECIPE-Index and nutrition content per 100 g according to the brand within semolina salads (n = 31).
Table S21. Comparison of nutriRECIPE-Index and nutrition content per 100 g according to the price range within semolina salads (n = 31).
Table S22. Comparison of nutriRECIPE-Index and nutrition content per 100 g according to the diet within potato salads (n = 31).
Table S23. Comparison of nutriRECIPE-Index and nutrition content per 100 g according to the dressing base within potato salads (n = 31).
Table S24. Comparison of nutriRECIPE-Index and nutrition content per 100 g according to the brand within potato salads (n = 31).
Table S25. Comparison of nutriRECIPE-Index and nutrition content per 100 g according to the price range within potato salads (n = 31).
Table S26. Comparison of nutriRECIPE-Index and nutrition content per 100 g according to the diet within pasta salads (n = 18).

Table S27. Comparison of nutriRECIPE-Index and nutrition content per 100 g according to the dressing base within pasta salads (n = 18).
Table S28. Comparison of nutriRECIPE-Index and nutrition content per 100 g according to the brand within pasta salads (n = 18).
Table S29. Comparison of nutriRECIPE-Index and nutrition content per 100 g according to the price range within pasta salads (n = 18).
Table S30. Comparison of nutriRECIPE-Index and nutrition content per 100 g according to the source of protein within protein-based salads (n = 45).
Table S31. Comparison of nutriRECIPE-Index and nutrition content per 100 g according to the salad base within protein-based salads (n = 45).
Table S32. Comparison of nutriRECIPE-Index and nutrition content per 100 g according to the dressing base within meat-based salads (n = 28).
Table S33. Comparison of nutriRECIPE-Index and nutrition content per 100 g according to the brand within meat-based salads (n = 28).
Table S34. Comparison of nutriRECIPE-Index and nutrition content per 100 g according to the price range within meat-based salads (n = 28).

Supplemental Figures

Figure S1. Decision tree for determining the appropriate test.

Table S2. Manufacturers' responses regarding the determination methods.

Salad number	Main group	Salad name	Manufacturer name	Method of determination	nutriRECIPE-Index aggregated approach	nutriRECIPE-Index disaggregated approach
1	Leaf salads	SALAT Nizza	Thurländer Salate und Feinkost GmbH	Analyses	115.1 %	110.3 %
2	Leaf salads	SALAT Caprese	Thurländer Salate und Feinkost GmbH	Analyses	105.9 %	109.3 %
3	Leaf salads	SALAT Caesar	Thurländer Salate und Feinkost GmbH	Analyses	83.5 %	54.7 %
4	Leaf salads	SALAT Italiana	Thurländer Salate und Feinkost GmbH	Analyses	89.9 %	69.2 %
5	Leaf salads	SALAT Kunterbunt	Thurländer Salate und Feinkost GmbH	Analyses	82.8 %	82.8 %
6	Leaf salads	SALAT Classico	Thurländer Salate und Feinkost GmbH	Analyses	73.5 %	77.7 %
7	Leaf salads	SALAT Bulgur	Thurländer Salate und Feinkost GmbH	Analyses	77.3 %	69.4 %
8	Leaf salads	SALAT Hellas	Thurländer Salate und Feinkost GmbH	Analyses	82.4 %	72.9 %
9	Leaf salads	SALAT Mexicana	Thurländer Salate und Feinkost GmbH	Analyses	77.5 %	75.0 %
10	Leaf salads	SALAT & QUINOA Schinken-Ziegenkäse	Thurländer Salate und Feinkost GmbH	Analyses	64.6 %	71.2 %
11	Leaf salads	SALAT & PASTA Thunfisch-Mais	Thurländer Salate und Feinkost GmbH	Analyses	72.0 %	57.4 %
12	Leaf salads	SALAT & PASTA Tomate-Mozzarella	Thurländer Salate und Feinkost GmbH	Analyses	68.1 %	76.8 %

Salad number	Main group	Salad name	Manufacturer name	Method of determination	nutriRECIPE-Index aggregated approach	nutriRECIPE-Index disaggregated approach
13	Leaf salads	SNACK Salat mit Paprika & Mais	Thurländer Salate und Feinkost GmbH	Analyses	89.6 %	89.6 %
14	Leaf salads	SNACK Salat mit Weißkraut & Tomate	Thurländer Salate und Feinkost GmbH	Analyses	88.1 %	88.1 %
15	Leaf salads	CAESAR+PASTA	GARTENFRISCH Jung GmbH	Verified	69.6 %	63.8 %
16	Leaf salads	Käse & Ei	GARTENFRISCH Jung GmbH	Verified	86.5 %	83.6 %
17	Leaf salads	CAPRESE+PASTA	GARTENFRISCH Jung GmbH	Verified	44.2 %	58.8 %
18	Leaf salads	ZIEGE+PASTA	GARTENFRISCH Jung GmbH	Verified	41.0 %	47.5 %
19	Leaf salads	Snackbox Vegetarisch	GARTENFRISCH Jung GmbH	Verified	124.2 %	124.2 %
20	Leaf salads	Snackbox Pute & Mais	GARTENFRISCH Jung GmbH	Verified	107.8 %	104.9 %
21	Leaf salads	SALAT MIT THUNFISCH UND SYLTER ART DRESSING	GARTENFRISCH Jung GmbH	Verified	102.0 %	96.4 %
22	Leaf salads	SALAT GARTENSALAT	GARTENFRISCH Jung GmbH	Verified	78.3 %	74.7 %
23	Leaf salads	SALAT KÄSE-SCHINKEN	GARTENFRISCH Jung GmbH	Verified	90.2 %	74.9 %
24	Leaf salads	Saladbowl Walnuss	H.Funken GmbH & Co. KG	Verified	94.2 %	90.8 %
25	Raw food salads	Tomaten-Mozzarella Snack	H.Funken GmbH & Co. KG	Verified	91.7 %	86.9 %
26	Leaf salads	Saladbowl Kentucky	H.Funken GmbH & Co. KG	Verified	118.4 %	124.1 %
27	Leaf salads	HACKBÄLLCHEN & NUDELN	GARTENFRISCH Jung GmbH	Verified	39.3 %	40.3 %
28	Leaf salads	Kleiner Mahlzeitsalat Gegrilltes Gemüse mit Couscous und Harissa-Dressing	Tacken GmbH	Verified	70.0 %	70.1 %
29	Leaf salads	Kleiner Mahlzeitsalat Guacamole mit Chili Mais-Chips und Avocado Creme	Tacken GmbH	Verified	79.7 %	77.1 %

Salad number	Main group	Salad name	Manufacturer name	Method of determination	nutriRECIPE-Index aggregated approach	nutriRECIPE-Index disaggregated approach
30	Leaf salads	Kleiner Mahlzeitsalat Gyros & Krautsalat mit Oliven und Knoblauch-Joghurt-Dressing	Tacken GmbH	Verified	83.3 %	68.9 %
31	Leaf salads	Bauern Snack Salat	Tacken GmbH	Verified	58.0 %	68.3 %
32	Leaf salads	Bunter Snack Salat	Tacken GmbH	Verified	75.7 %	75.2 %
33	Leaf salads	Garten Snack Salat	Tacken GmbH	Verified	63.1 %	79.3 %
34	Leaf salads	Veggie Picknick VEGETARISCHE MÜHLEN FRIKADELLEN - MAIS	Bonduelle Deutschland GmbH	No information	66.6 %	62.8 %
35	Leaf salads	Composé	Gartenfrisch Jung GmbH	Verified	71.1 %	84.1 %
36	Leaf salads	Deftige Auszeit SCHINKEN-KÄSE	Bonduelle Deutschland GmbH	No information	77.7 %	65.4 %
37	Leaf salads	DELICATESSA Gourmetsalat Käse & Ei mit Joghurt-Dressing	Gartenfrisch Jung GmbH	Verified	75.0 %	75.3 %
38	Leaf salads	Snackbox Käse & Ei	Gartenfrisch Jung GmbH	Verified	140.6 %	143.0 %
39	Leaf salads	Salat Menü mit Fusilli, Tomate & Mozzarella	Fresh Care Convenience GmbH	Literature	70.3 %	70.3 %
40	Leaf salads	Salat Menü mit Quinoa	Fresh Care Convenience GmbH	Literature	55.1 %	57.6 %
41	Leaf salads	Salat Menü mit Thunfisch	Fresh Care Convenience GmbH	Literature	93.3 %	120.5 %
42	Leaf salads	Salat Menü mit Penne, Hähnchen & Grana Padano	Fresh Care Convenience GmbH	Literature	54.8 %	67.0 %
43	Leaf salads	Salat Menü mit Garnelen	Fresh Care Convenience GmbH	Literature	47.3 %	97.6 %
44	Leaf salads	Frische Salate nach Sylter Art	Fresh Care Convenience GmbH	Literature	50.8 %	51.1 %
45	Leaf salads	Frische Salate Florence Art	Fresh Care Convenience GmbH	Literature	84.8 %	90.7 %
46	Leaf salads	Frische Salate nach Griechischer Art	Fresh Care Convenience GmbH	Literature	84.7 %	55.1 %

Salad number	Main group	Salad name	Manufacturer name	Method of determination	nutriRECIPE-Index aggregated approach	nutriRECIPE-Index disaggregated approach
47	Leaf salads	Frische Salate Käse & Ei	Fresh Care Convenience GmbH	Literature	96.8 %	108.4 %
48	Leaf salads	Frische Salate Käse & Schinken	Fresh Care Convenience GmbH	Literature	75.1 %	80.3 %
49	Protein-based salads	Bunter Bohnensalat mit Käse	Bon Fraîche Feinkost GmbH	No information	68.5 %	68.6 %
50	Raw food salads	Rote Betesalat	Dr. Schrödter	Combination	57.1 %	57.1 %
51	Raw food salads	Gurkensalat	Dr. Schrödter	Combination	9.0 %	9.0 %
52	Raw food salads	Möhrensalat	Dr. Schrödter	Combination	26.4 %	26.4 %
53	Raw food salads	Rettichsalat	Dr. Schrödter	Combination	48.8 %	48.8 %
54	Raw food salads	Zazikisalat	Dr. Schrödter	Combination	48.9 %	48.9 %
55	Raw food salads	Broccolisalat	Dr. Schrödter	Combination	45.8 %	45.8 %
56	Leaf salads	NUDEL HACKBÄLLCHEN SALAT	Kaufland Warenhandel GmbH & Co. KG	No information	41.9 %	40.3 %
57	Leaf salads	KARTOFFEL HÄHNCHEN SALAT	Kaufland Warenhandel GmbH & Co. KG	No information	62.0 %	55.8 %
58	Leaf salads	KARTOFFEL RINDFLEISCH SALAT	Kaufland Warenhandel GmbH & Co. KG	No information	92.6 %	90.1 %
59	Leaf salads	BULGUR ZIEGENKÄSE CRANBERRY SALAT	Kaufland Warenhandel GmbH & Co. KG	No information	42.4 %	58.3 %
60	Leaf salads	GOUDA SCHINKEN SALAT	Kaufland Warenhandel GmbH & Co. KG	No information	87.2 %	95.2 %
61	Leaf salads	TOMATE MAIS SALAT	Kaufland Warenhandel GmbH & Co. KG	No information	60.0 %	80.1 %

Salad number	Main group	Salad name	Manufacturer name	Method of determination	nutriRECIPE-Index aggregated approach	nutriRECIPE-Index disaggregated approach
62	Leaf salads	HÄHNCHEN GOUDA SALAT	Kaufland Warenhandel GmbH & Co. KG	No information	51.6 %	42.7 %
63	Leaf salads	KÄSE CROUTONS SALAT	Kaufland Warenhandel GmbH & Co. KG	No information	73.4 %	73.6 %
64	Leaf salads	QUINOA ZIEGENKÄSE SALAT	Kaufland Warenhandel GmbH & Co. KG	No information	62.3 %	57.2 %
65	Leaf salads	BIFTEKI TOMATEN SALAT	Kaufland Warenhandel GmbH & Co. KG	No information	60.0 %	63.8 %
66	Protein-based salads	EDAMAME SALAT	Natsu Foods GmbH & Co. KG	Analyses	93.3 %	105.0 %
67	Raw food salads	Möhrensalat	Havita Berlin Frischgemüse GmbH	Verified	64.0 %	39.3 %
68	Raw food salads	Gurkensalat	Havita Berlin Frischgemüse GmbH	Verified	97.4 %	99.2 %
69	Protein-based salads	Bohnensalat	Havita Berlin Frischgemüse GmbH	Verified	89.5 %	81.2 %
70	Leaf salads	frischer Salatmix GALLO	Havita Berlin Frischgemüse GmbH	Verified	57.6 %	54.1 %
71	Leaf salads	frischer Salatmix VEGETARIANO	Havita Berlin Frischgemüse GmbH	Verified	61.5 %	64.7 %
72	Leaf salads	Bunter Snack Salat	Havita Berlin Frischgemüse GmbH	Verified	58.6 %	62.8 %
73	Leaf salads	Garten Snack Salat	Havita Berlin Frischgemüse GmbH	Verified	65.8 %	69.1 %
74	Leaf salads	Bauern Snack Salat	Havita Berlin Frischgemüse GmbH	Verified	65.9 %	70.3 %

Salad number	Main group	Salad name	Manufacturer name	Method of determination	nutriRECIPE-Index aggregated approach	nutriRECIPE-Index disaggregated approach
75	Leaf salads	Winter-Mix Salat	Havita Berlin Frischgemüse GmbH	Verified	134.2 %	134.2 %
76	Raw food salads	Quattro Insalata	Havita Berlin Frischgemüse GmbH	Verified	97.6 %	91.5 %
77	Leaf salads	frischer Salatmix FRANCESCO	Havita Berlin Frischgemüse GmbH	Verified	57.8 %	71.9 %
78	Leaf salads	NIZZA SALAT	Havita Berlin Frischgemüse GmbH	Verified	88.0 %	79.9 %
79	Leaf salads	GUACAMOLE SALAT	Havita Berlin Frischgemüse GmbH	Verified	78.3 %	98.1 %
80	Leaf salads	ORZO SALAT	Havita Berlin Frischgemüse GmbH	Verified	44.3 %	44.1 %
81	Leaf salads	GRECO SALAT	Havita Berlin Frischgemüse GmbH	Verified	56.0 %	65.3 %
82	Leaf salads	CEASAR SALAT	Havita Berlin Frischgemüse GmbH	Verified	84.7 %	74.7 %
83	Raw food salads	Rohkostsalat	Havita Berlin Frischgemüse GmbH	Verified	85.7 %	85.7 %
84	Raw food salads	Rote Bete Salat	Havita Berlin Frischgemüse GmbH	Verified	27.1 %	27.1 %
85	Raw food salads	Mediterraner Tomaten-Gurken-Salat	Havita Berlin Frischgemüse GmbH	Verified	91.1 %	86.0 %
86	Raw food salads	Schopska Salat	Havita Berlin Frischgemüse GmbH	Verified	96.7 %	94.2 %
87	Raw food salads	Brokkoli-Salat	Havita Berlin Frischgemüse GmbH	Verified	79.2 %	78.8 %

Salad number	Main group	Salad name	Manufacturer name	Method of determination	nutriRECIPE-Index aggregated approach	nutriRECIPE-Index disaggregated approach
88	Leaf salads	PASTA SALAT	Fresh Care Convenience GmbH	Literature	80.0 %	80.9 %
89	Leaf salads	QUINOA SALAT	Fresh Care Convenience GmbH	Literature	44.3 %	49.8 %
90	Leaf salads	Käse-Schinken Salat	Fresh Care Convenience GmbH	Literature	71.1 %	76.1 %
91	Leaf salads	Garten Salat	Fresh Care Convenience GmbH	Literature	75.1 %	75.6 %
92	Leaf salads	Grana Padano Salat	Fresh Care Convenience GmbH	Literature	76.1 %	61.8 %
93	Leaf salads	Hähnchen Salat	Fresh Care Convenience GmbH	Literature	66.0 %	82.0 %
94	Leaf salads	Caesar Salat	Fresh Care Convenience GmbH	Literature	82.9 %	72.2 %
95	Raw food salads	Weißkrautsalat	Heinrich Kühlmann GmbH & Co. KG	No information	50.2 %	50.2 %
96	Raw food salads	Gurkensalat	Heinrich Kühlmann GmbH & Co. KG	No information	24.1 %	24.1 %
97	Leaf salads	SALAT BOX Vegetarisch	Havita Berlin Frischgemüse GmbH	Verified	66.4 %	62.5 %
98	Leaf salads	SALAT BOX Griechisch	Havita Berlin Frischgemüse GmbH	Verified	70.6 %	70.1 %
99	Leaf salads	SALAT BOX Käse-Schinken	Havita Berlin Frischgemüse GmbH	Verified	57.1 %	71.6 %
100	Leaf salads	SALAT BOX Hähnchenbrust	Havita Berlin Frischgemüse GmbH	Verified	53.6 %	50.0 %
101	Leaf salads	PREMIUM SALAT CHEF	NICO Frischprodukte GmbH	No information	124.8 %	115.1 %
102	Leaf salads	PREMIUM SALAT HIRTENKÄSE	NICO Frischprodukte GmbH	No information	106.0 %	96.8 %
103	Leaf salads	PREMIUM SALAT CAESAR	NICO Frischprodukte GmbH	No information	116.4 %	90.7 %
104	Leaf salads	MAHLZEIT SALAT MAIS	NICO Frischprodukte GmbH	No information	122.2 %	112.0 %

Salad number	Main group	Salad name	Manufacturer name	Method of determination	nutriRECIPE-Index aggregated approach	nutriRECIPE-Index disaggregated approach
105	Leaf salads	MAHLZEIT SALAT HÄHNCHEN	NICO Frischprodukte GmbH	No information	128.1 %	120.7 %
106	Leaf salads	MAHLZEIT SALAT THUNFISCH	NICO Frischprodukte GmbH	No information	143.2 %	154.7 %
107	Raw food salads	COLE SLAW SALAT CUP	NICO Frischprodukte GmbH	No information	113.8 %	113.8 %
108	Leaf salads	Junior Salat Box	NICO Frischprodukte GmbH	No information	139.8 %	139.8 %
109	Leaf salads	Single Salat Box	NICO Frischprodukte GmbH	No information	139.3 %	139.8 %
110	Leaf salads	SALATCUP HÄHNCHEN MIT SENF-DRESSING	Gartenfrisch Jung GmbH	Verified	75.9 %	74.8 %
111	Leaf salads	QUINOA SALAT MIT SCHINKEN, ZIEGENFRISCHKÄSE, CROUTONS UND SENF-DRESSING	Fresh-Care Convenience BV	Literature	51.7 %	57.7 %
112	Leaf salads	PASTA SALAT MIT FUSILLI, HALBGETROCKNETEN TOMATEN, MOZZARELLA UND KRÄUTER-DRESSING	Fresh-Care Convenience BV	Literature	68.6 %	60.7 %
113	Leaf salads	SALATCUP KÄSE & SCHINKEN MIT JOGHURT-DRESSING	Gartenfrisch Jung GmbH	Verified	93.7 %	84.1 %
114	Leaf salads	PASTA SALAT MIT PENNE, HÄHNCHEN, HARTKÄSE UND JOGHURT-DRESSING	Fresh-Care Convenience BV	Literature	56.4 %	59.7 %
115	Raw food salads	WEISSKRAUT SALAT	Popp Feinkost GmbH	Verified	71.5 %	71.5 %
116	Leaf salads	FEIERABEND Laune CAESAR ART	Bonduelle Deutschland GmbH	No information	79.8 %	78.9 %
117	Raw food salads	ALGENSALAT WAKAME	Deutsche See GmbH	No information	69.6 %	83.7 %
118	Raw food salads	BIO PUR KAROTTENSALAT	Heinrich Kühlmann GmbH & Co. KG	No information	53.5 %	53.5 %

Salad number	Main group	Salad name	Manufacturer name	Method of determination	nutriRECIPE-Index aggregated approach	nutriRECIPE-Index disaggregated approach
119	Raw food salads	Krautsalat	GLOBUS SB-Warenhaus Holding GmbH & Co. KG	No information	41.3 %	41.3 %
120	Raw food salads	Krautsalat	Nadler Feinkost GmbH	No information	37.7 %	37.7 %
121	Raw food salads	Meistersalat	Popp Feinkost GmbH	Verified	50.8 %	50.8 %
122	Raw food salads	COLESLAW-SALAT	Popp Feinkost GmbH	Verified	29.1 %	29.1 %
123	Raw food salads	Frischer Krautsalat "GRIECHISCHE ART"	Popp Feinkost GmbH	Verified	50.8 %	50.8 %
124	Raw food salads	Feiner Gurkensalat in Joghurtdressing	Popp Feinkost GmbH	Verified	26.3 %	26.3 %
125	Raw food salads	SPREEWÄLDER GURKENSALAT	Golßener Fleisch- und Wurstwaren GmbH & Co. Produktions KG	Analyses	20.2 %	20.2 %
126	Raw food salads	Bunter Rohkostsalat	Dr. Schrödter Feinkost GmbH	Combination	69.9 %	69.9 %
127	Raw food salads	Karottensalat	Dr. Schrödter Feinkost GmbH	Combination	30.1 %	30.1 %
128	Raw food salads	Krautsalat nach griechischer Art	Dr. Schrödter Feinkost GmbH	Combination	63.6 %	63.6 %
129	Leaf salads	SALAT Hähnchen & Mais	Thurländer Salate und Feinkost GmbH	Analyses	68.7 %	69.9 %
130	Leaf salads	SALAT Schinken & Käse	Thurländer Salate und Feinkost GmbH	Analyses	79.9 %	85.1 %
131	Leaf salads	GARTENSALAT	Thurländer Salate und Feinkost GmbH	Analyses	92.3 %	92.3 %
132	Leaf salads	GEFLÜGEL-FRIKADELLEN-MAIS SALAT	Thurländer Salate und Feinkost GmbH	Analyses	66.4 %	49.2 %

Salad number	Main group	Salad name	Manufacturer name	Method of determination	nutriRECIPE-Index aggregated approach	nutriRECIPE-Index disaggregated approach
133	Leaf salads	THUNFISCH SALAT	Thurländer Salate und Feinkost GmbH	Analyses	50.3 %	30.7 %
134	Leaf salads	Hähnchen-Hartkäse Salat	Thurländer Salate und Feinkost GmbH	Analyses	71.7 %	52.1 %
135	Raw food salads	Rahmgurkensalat mit Dill und Joghurtsoße	Tacken GmbH	Verified	33.5 %	54.6 %
136	Raw food salads	Karotten-Apfelsalat in feinen Streifen	Tacken GmbH	Verified	29.5 %	24.2 %
137	Raw food salads	BIO PUR KRAUTSALAT	Heinrich Kühlmann GmbH & Co. KG	No information	62.8 %	62.8 %
138	Leaf salads	DELICATESSA	Gartenfrisch Jung GmbH	Verified	73.4 %	63.3 %
139	Protein-based salads	Ungarischer Wurstsalat	Hans Adler oHG	Analyses	55.4 %	54.9 %
140	Protein-based salads	Schweizer Wurstsalat	Hans Adler oHG	Analyses	59.0 %	57.0 %
141	Starch-based salads	Bulgursalat Klassisch	Heinrich Kühlmann GmbH & Co. KG	No information	64.0 %	86.9 %
142	Starch-based salads	Couscoussalat Paprika& Rosine	Heinrich Kühlmann GmbH & Co. KG	No information	41.2 %	36.5 %
143	Starch-based salads	Bulgursalat Bohne und Olive	Heinrich Kühlmann GmbH & Co. KG	No information	55.1 %	71.9 %
144	Raw food salads	BBQ SALAT COLESLAW	Heinrich Kühlmann GmbH & Co. KG	No information	46.5 %	46.5 %
145	Starch-based salads	BBQ SALAT SOUTH CAROLINA STYLE	Heinrich Kühlmann GmbH & Co. KG	No information	30.0 %	29.9 %
146	Raw food salads	Feiner Weißkrautsalat	Homann Feinkost GmbH	Combination	65.1 %	65.1 %

Salad number	Main group	Salad name	Manufacturer name	Method of determination	nutriRECIPE-Index aggregated approach	nutriRECIPE-Index disaggregated approach
147	Starch-based salads	Feiner Nudelsalat	Homann Feinkost GmbH	Combination	52.6 %	28.9 %
148	Starch-based salads	Klassischer Kartoffelsalat	Homann Feinkost GmbH	Combination	41.8 %	41.8 %
149	Raw food salads	Bunter Partysalat	Homann Feinkost GmbH	Combination	48.1 %	48.1 %
150	Starch-based salads	Bunter Kartoffelsalat	Thüringer Fischfeinkost Gebrüder Hopf GmbH	Combination	43.3 %	43.3 %
151	Starch-based salads	Kartoffelsalat Joghurtcreme	Homann Feinkost GmbH	Combination	41.3 %	39.9 %
152	Starch-based salads	Kartoffelsalat Crème FraÎche	Homann Feinkost GmbH	Combination	39.1 %	47.1 %
153	Protein-based salads	Delikatess Fleischsalat	Füngers Feinkost GmbH & Co. KG	Verified	26.8 %	28.3 %
154	Protein-based salads	Budapester Salat	Homann Feinkost GmbH	Combination	27.0 %	34.0 %
155	Protein-based salads	Hähnchenbrustsalat	Homann Feinkost GmbH	Combination	46.5 %	46.5 %
156	Starch-based salads	Nudelsalat	Heinrich Kühlmann GmbH & Co. KG	No information	19.6 %	20.6 %
157	Protein-based salads	Delikatess Fleischsalat	Füngers Feinkost GmbH & Co. KG	Verified	33.7 %	33.8 %
158	Protein-based salads	Cremiger Fleischsalat	Homann Feinkost GmbH	Combination	36.5 %	34.0 %
159	Protein-based salads	Herzhafter Fleischsalat	Homann Feinkost GmbH	Combination	37.7 %	37.7 %
160	Protein-based salads	Geflügel Fleischsalat	Homann Feinkost GmbH	Combination	54.2 %	54.2 %
161	Raw food salads	Coleslaw	Heinrich Kühlmann GmbH & Co. KG	No information	45.2 %	45.5 %

Salad number	Main group	Salad name	Manufacturer name	Method of determination	nutriRECIPE-Index aggregated approach	nutriRECIPE-Index disaggregated approach
162	Starch-based salads	Kartoffelsalat	Heinrich Kühlmann GmbH & Co. KG	No information	50.8 %	50.8 %
163	Starch-based salads	COUSCOUS SALAT	Natsu Foods GmbH & Co. KG	Analyses	81.8 %	88.6 %
164	Starch-based salads	Kartoffelsalat	Nadler Feinkost GmbH	No information	44.9 %	44.9 %
165	Starch-based salads	Kartoffelsalat Westfälischer Art	Homann Feinkost GmbH	Combination	37.2 %	44.5 %
166	Starch-based salads	Feiner Nudelsalat	Homann Feinkost GmbH	Combination	24.0 %	26.2 %
167	Starch-based salads	Feiner Pellkartoffelsalat	Homann Feinkost GmbH	Combination	41.3 %	41.3 %
168	Starch-based salads	Pikanter Nudelsalat	Homann Feinkost GmbH	Combination	27.6 %	51.4 %
169	Leaf salads	Schinken Gouda Salat	GARTENFRISCH Jung GmbH	Verified	95.8 %	95.2 %
170	Leaf salads	Hähnchen Gouda Salat	GARTENFRISCH Jung GmbH	Verified	49.7 %	44.3 %
171	Starch-based salads	Deftiger Kartoffelsalat "Sächsische Art"	Popp Feinkost GmbH	Verified	44.6 %	48.1 %
172	Starch-based salads	Pellkartoffelsalat Ei & Gurke	Popp Feinkost GmbH	Verified	39.8 %	39.8 %
173	Starch-based salads	Feiner Nudelsalat Schinkenwurst & Gemüse	Popp Feinkost GmbH	Verified	30.9 %	27.4 %
174	Starch-based salads	Herzhafter Kartoffelsalat Speck & Zwiebel	Popp Feinkost GmbH	Verified	42.5 %	43.0 %
175	Protein-based salads	Delikatess Fleischsalat	Hamker Vertriebsgesellschaft mbH	Combination	30.6 %	30.5 %
176	Starch-based salads	Klassischer Kartoffelsalat	Hamker Vertriebsgesellschaft mbH	Combination	42.5 %	42.5 %

Salad number	Main group	Salad name	Manufacturer name	Method of determination	nutriRECIPE-Index aggregated approach	nutriRECIPE-Index disaggregated approach
177	Starch-based salads	TABOULÈ	Heinrich Kühlmann GmbH & Co. KG	No information	51.8 %	48.3 %
178	Starch-based salads	BULGURSALAT	Heinrich Kühlmann GmbH & Co. KG	No information	74.8 %	105.7 %
179	Starch-based salads	BULGURSALAT	Heinrich Kühlmann GmbH & Co. KG	No information	69.9 %	84.7 %
180	Starch-based salads	SNACK BOWL	Heinrich Kühlmann GmbH & Co. KG	No information	64.6 %	62.2 %
181	Starch-based salads	SNACK BOWL	Heinrich Kühlmann GmbH & Co. KG	No information	65.5 %	63.9 %
182	Starch-based salads	SNACK BOWL	Heinrich Kühlmann GmbH & Co. KG	No information	86.4 %	85.1 %
183	Starch-based salads	Kartoffel Salat	Kaufland Warenhandel GmbH & Co. KG	No information	64.0 %	69.7 %
184	Protein-based salads	Ungarischer Wurstsalat mit Paprika	Hans Adler OHG	Analyses	56.2 %	52.1 %
185	Starch-based salads	Couscous-Hummus Paprika Salat mit Paprika und Oliven	Perla Deutschland GmbH	No information	61.7 %	72.9 %
186	Starch-based salads	THAI-STYLE Nudelsalat	Heinrich Kühlmann GmbH & Co. KG	No information	49.5 %	64.1 %
187	Raw food salads	Coleslaw Snacksalat	Heinrich Kühlmann GmbH & Co. KG	No information	41.6 %	25.4 %
188	Starch-based salads	Thunfisch Pasta Salat	HFC GmbH	Verified	55.2 %	68.9 %
189	Raw food salads	Rotkrautsalat	Thüringer Fischfeinkost Gebrüder Hopf GmbH	Combination	39.7 %	39.7 %

Salad number	Main group	Salad name	Manufacturer name	Method of determination	nutriRECIPE-Index aggregated approach	nutriRECIPE-Index disaggregated approach
190	Protein-based salads	Geflügelsalat	Thüringer Fischfeinkost Gebrüder Hopf GmbH	Combination	36.9 %	36.9 %
191	Starch-based salads	Bulgursalat	EDEKA ZENTRALE AG & Co.KG	Analyses	81.5 %	74.0 %
192	Starch-based salads	Bulgursalat	EDEKA ZENTRALE AG & Co.KG	Analyses	70.3 %	73.9 %
193	Protein-based salads	Geflügelsalat	EDEKA ZENTRALE AG & Co.KG	Analyses	23.9 %	30.3 %
194	Starch-based salads	Kartoffelsalat	EDEKA ZENTRALE AG & Co.KG	Analyses	40.4 %	39.3 %
195	Raw food salads	Farmer Salat	EDEKA ZENTRALE AG & Co.KG	Analyses	31.7 %	41.9 %
196	Starch-based salads	Taboulé Salat	ISANA Naturfeinkost GmbH & Co. KG	Analyses	82.3 %	82.2 %
197	Protein-based salads	Bio-Eiersalat	Popp Feinkost GmbH	Verified	83.7 %	83.7 %
198	Raw food salads	Bio-Farmersalat	Popp Feinkost GmbH	Verified	35.6 %	35.6 %
199	Starch-based salads	Bio-Kartoffelsalat	Popp Feinkost GmbH	Verified	77.3 %	77.3 %
200	Starch-based salads	Bio-Kartoffelsalat	Popp Feinkost GmbH	Verified	57.5 %	57.5 %
201	Starch-based salads	Bio-Nudelsalat	Popp Feinkost GmbH	Verified	55.1 %	67.9 %
202	Protein-based salads	Bio-Curry-Geflügelsalat	Popp Feinkost GmbH	Verified	40.6 %	41.0 %
203	Starch-based salads	Kartoffel-Salat "TEGERNSEE"	Dahlhoff Feinkost GmbH	Combination	40.5 %	58.6 %

Salad number	Main group	Salad name	Manufacturer name	Method of determination	nutriRECIPE-Index aggregated approach	nutriRECIPE-Index disaggregated approach
204	Starch-based salads	Omas Kartoffelsalat	Heinrich Kühlmann GmbH & Co. KG	No information	50.7 %	50.7 %
205	Starch-based salads	BBQ SALAD	Heinrich Kühlmann GmbH & Co. KG	No information	45.7 %	34.9 %
206	Starch-based salads	Unser Klarer Speck-Kartoffelsalat	Rüma Feinkost GmbH & Co. KG	Verified	50.7 %	51.5 %
207	Starch-based salads	Kartoffel Salat	Alnatura GmbH	Combination	49.2 %	49.2 %
208	Leaf salads	Pute & Mais Salat	GARTENFRISCH Jung GmbH	Verified	107.8 %	104.9 %
209	Starch-based salads	Couscous-Salat	ISANA Naturfeinkost GmbH & Co. KG	Analyses	67.7 %	87.6 %
210	Raw food salads	Algen-Salat	ISANA Naturfeinkost GmbH & Co. KG	Analyses	87.8 %	90.3 %
211	Protein-based salads	Salat von dreierlei Linsen	GROSSMANN Feinkost GmbH	Verified	91.8 %	102.2 %
212	Starch-based salads	Bulgur-Salat "Kisir"	GROSSMANN Feinkost GmbH	Verified	107.6 %	76.0 %
213	Starch-based salads	Couscoussalat Oriental	GROSSMANN Feinkost GmbH	Verified	54.2 %	50.2 %
214	Starch-based salads	Nudelsalat	Heinrich Kühlmann GmbH & Co. KG	No information	30.0 %	31.8 %
215	Raw food salads	Unser Bauern-Salat	Rüma Feinkost GmbH & Co. KG	Verified	38.1 %	38.4 %
216	Raw food salads	Coleslaw Krautsalat	Homann Feinkost GmbH	Combination	30.5 %	30.5 %
217	Protein-based salads	Milder Eiersalat	Homann Feinkost GmbH	Combination	68.9 %	68.9 %
218	Starch-based salads	Taboulé Hühnchen Joghurt	HFC GmbH	Verified	66.7 %	48.7 %

Salad number	Main group	Salad name	Manufacturer name	Method of determination	nutriRECIPE-Index aggregated approach	nutriRECIPE-Index disaggregated approach
219	Protein-based salads	Asia-Glasnudel mit Kurkuma-Blumenkohl	HFC GmbH	Verified	101.2 %	118.1 %
220	Starch-based salads	Insalata Pasta Mediterranea	HFC GmbH	Verified	66.8 %	68.8 %
221	Starch-based salads	Insalata Pasta Pesto Pinienkerne	HFC GmbH	Verified	78.5 %	57.9 %
222	Starch-based salads	Insalata Pasta Tonno	HFC GmbH	Verified	86.4 %	73.9 %
223	Raw food salads	Thai-Carrots mit Roter Bete & Meerrettich	HFC GmbH	Verified	72.3 %	70.5 %
224	Leaf salads	Nudel Hackbällchen Salat	GARTENFRISCH Jung GmbH	Verified	42.7 %	40.3 %
225	Leaf salads	Pasta Mozzarella Salat	GARTENFRISCH Jung GmbH	Verified	57.9 %	64.4 %
226	Leaf salads	Tomate Mais Salat	GARTENFRISCH Jung GmbH	Verified	82.2 %	78.8 %
227	Protein-based salads	Eiersalat	Homann Feinkost GmbH	Combination	43.9 %	55.1 %
228	Protein-based salads	Eiersalat	EDEKA ZENTRALE AG & Co.KG	Analyses	52.2 %	53.1 %
229	Starch-based salads	Curry-Quinoa	Heinrich Kühlmann GmbH & Co. KG	No information	54.3 %	63.1 %
230	Starch-based salads	Kartoffelsalat	Heinrich Kühlmann GmbH & Co. KG	No information	68.1 %	67.2 %
231	Starch-based salads	Thai Nudelsalat	Heinrich Kühlmann GmbH & Co. KG	No information	40.9 %	67.6 %
232	Protein-based salads	Fleischsalat	Thüringer Fischfeinkost Gebrüder Hopf GmbH	Combination	34.1 %	34.1 %
233	Protein-based salads	Fleischsalat	Thüringer Fischfeinkost Gebrüder Hopf GmbH	Combination	33.6 %	33.6 %

Salad number	Main group	Salad name	Manufacturer name	Method of determination	nutriRECIPE-Index aggregated approach	nutriRECIPE-Index disaggregated approach
234	Starch-based salads	Kartoffelsalat	Thüringer Fischfeinkost Gebrüder Hopf GmbH	Combination	46.7 %	46.7 %
235	Starch-based salads	Bunter Kartoffelsalat	Thüringer Fischfeinkost Gebrüder Hopf GmbH	Combination	43.3 %	43.3 %
236	Starch-based salads	Kartoffelsalat Hamburger Art	Homann Feinkost GmbH	Combination	46.0 %	46.0 %
237	Protein-based salads	Herzhafter Fleischsalat	Homann Feinkost GmbH	Combination	42.0 %	35.6 %
238	Raw food salads	Fruchtiger Waldorfsalat	Homann Feinkost GmbH	Combination	47.8 %	47.8 %
239	Raw food salads	Weißkrautsalat	Homann Feinkost GmbH	Combination	37.4 %	37.4 %
240	Raw food salads	Feiner Coleslaw-Salat	Popp Feinkost GmbH	Verified	29.1 %	29.1 %
241	Protein-based salads	Feinster Fleischsalat	Popp Feinkost GmbH	Verified	25.5 %	25.5 %
242	Protein-based salads	Kräuter Fleischsalat	Popp Feinkost GmbH	Verified	30.7 %	29.8 %
243	Raw food salads	Farmersalat	Heinrich Kühlmann GmbH & Co. KG	No information	33.6 %	37.9 %
244	Starch-based salads	Omas Nudelsalat	Heinrich Kühlmann GmbH & Co. KG	No information	24.2 %	26.3 %
245	Starch-based salads	Bulgursalat	Heinrich Kühlmann GmbH & Co. KG	No information	84.6 %	65.8 %
246	Starch-based salads	Bulgur-Linsensalat	Heinrich Kühlmann GmbH & Co. KG	No information	81.9 %	69.8 %
247	Raw food salads	Coleslaw	Heinrich Kühlmann GmbH & Co. KG	No information	45.2 %	45.5 %

Salad number	Main group	Salad name	Manufacturer name	Method of determination	nutriRECIPE-Index aggregated approach	nutriRECIPE-Index disaggregated approach
248	Starch-based salads	Nudelsalat	Heinrich Kühlmann GmbH & Co. KG	No information	42.2 %	46.4 %
249	Protein-based salads	Alpenruf Wurstsalat Schweizer Art	Heinrich Kühlmann GmbH & Co. KG	No information	58.2 %	64.8 %
250	Protein-based salads	Alpenruf Wurstsalat Ungarischer Art	Heinrich Kühlmann GmbH & Co. KG	No information	51.9 %	55.6 %
251	Starch-based salads	Couscous-Salat	Bon Fraîche Feinkost GmbH	No information	62.7 %	61.9 %
252	Starch-based salads	Nudel-Salat	Bon Fraîche Feinkost GmbH	No information	60.1 %	81.9 %
253	Protein-based salads	Eiersalat	Voss Feinkost und Lebensmittel GmbH	No information	72.6 %	72.6 %
254	Protein-based salads	Fruchtiger Geflügelsalat	Voss Feinkost und Lebensmittel GmbH	No information	46.3 %	46.3 %
255	Protein-based salads	Eiersalat	Heinrich Kühlmann GmbH & Co. KG	No information	60.3 %	60.9 %
256	Protein-based salads	Brotaufstrich Ei-Bacon	Dr. Schrödter	Combination	60.8 %	60.8 %
257	Protein-based salads	Feinster Eiersalat	EDEKA ZENTRALE AG & Co. KG	Analyses	65.8 %	76.3 %
258	Protein-based salads	Feinster Geflügelsalat	EDEKA ZENTRALE AG & Co. KG	Analyses	33.7 %	47.1 %
259	Starch-based salads	Herzhafter Kartoffelsalat	EDEKA ZENTRALE AG & Co. KG	Analyses	36.0 %	39.3 %
260	Protein-based salads	Geflügelsalat	Heinrich Kühlmann GmbH & Co. KG	No information	51.9 %	38.5 %

Salad number	Main group	Salad name	Manufacturer name	Method of determination	nutriRECIPE-Index aggregated approach	nutriRECIPE-Index disaggregated approach
261	Starch-based salads	Couscous-Salat	ISANA Naturfeinkost GmbH & Co. KG	Analyses	66.4 %	72.4 %
262	Starch-based salads	Taboulé-Salat	ISANA Naturfeinkost GmbH & Co. KG	Analyses	82.3 %	82.1 %
263	Protein-based salads	EDAMAME und Quinoa	Natsu Foods GmbH & Co. KG	Analyses	93.3 %	105.3 %
264	Protein-based salads	Rindfleischsalat	Popp Feinkost GmbH	Verified	75.6 %	73.7 %
265	Protein-based salads	Herrensalat	Golßener und mago Vertriebs oHG	Analyses	63.3 %	48.0 %
266	Protein-based salads	Rindfleischsalat	Golßener und mago Vertriebs oHG	Analyses	93.7 %	88.0 %
267	Raw food salads	Salatbowl Quinoa-Kichererbse	dm-drogerie markt GmbH + Co. KG	No information	99.8 %	99.5 %
268	Protein-based salads	Salatbowl Tex Mex	dm-drogerie markt GmbH + Co. KG	No information	102.4 %	114.9 %
269	Starch-based salads	Kartoffelsalat	Heinrich Kühlmann GmbH & Co. KG	No information	54.4 %	51.5 %
270	Starch-based salads	Kartoffelsalat	Nadler Feinkost GmbH	No information	48.8 %	49.9 %
271	Starch-based salads	Speckkartoffelsalat	Heinrich Kühlmann GmbH & Co. KG	No information	45.0 %	45.1 %
272	Starch-based salads	Würziger Kartoffelsalat	HOMANN Feinkost GmbH	Combination	43.1 %	43.1 %
273	Starch-based salads	Couscous-Salat	Alnatura GmbH	Combination	88.5 %	74.8 %
274	Starch-based salads	Bulgur Salat	Alnatura GmbH	Combination	83.2 %	123.1 %

Salad number	Main group	Salad name	Manufacturer name	Method of determination	nutriRECIPE-Index aggregated approach	nutriRECIPE-Index disaggregated approach
275	Protein-based salads	Geflügelsalat	Alnatura GmbH	Combination	47.8 %	47.8 %
276	Starch-based salads	Getreidesalat	Stengel GmbH & Co. KG	No information	95.6 %	100.7 %
277	Starch-based salads	Bulgursalat	Stengel GmbH & Co. KG	No information	98.8 %	79.4 %
278	Starch-based salads	BOWL Mexiko	Heinrich Kühlmann GmbH & Co. KG	No information	75.7 %	78.0 %
279	Starch-based salads	BOWL Couscous	Heinrich Kühlmann GmbH & Co. KG	No information	65.7 %	64.5 %
280	Starch-based salads	QUINOA Karotten Salat	Heinrich Kühlmann GmbH & Co. KG	No information	70.6 %	79.1 %
281	Protein-based salads	Linsen Weisskäse Salat	Heinrich Kühlmann GmbH & Co. KG	No information	83.1 %	92.6 %
282	Starch-based salads	Kartoffelsalat	Golßener Fleisch- und Wurstwaren GmbH & Co. Produktions KG	Analyses	39.3 %	39.3 %
283	Starch-based salads	Rustikaler Kartoffelsalat	Bon Fraîche Feinkost GmbH	No information	45.2 %	59.4 %
284	Starch-based salads	Bulgur Taboulé Salat	dm-drogerie markt GmbH + Co. KG	No information	81.1 %	63.5 %
285	Starch-based salads	Salat Quinoa mit Feta	Natsu Foods GmbH & Co. KG	Analyses	82.3 %	80.1 %
286	Starch-based salads	Salat Bulgur mit Kichererbsen	Natsu Foods GmbH & Co. KG	Analyses	90.2 %	74.9 %
287	Protein-based salads	Salat Linsen mit Ziegenkäse	Natsu Foods GmbH & Co. KG	Analyses	89.2 %	93.9 %
288	Starch-based salads	Salat Dinkel-Quinoa mit Edamame	Natsu Foods GmbH & Co. KG	Analyses	99.4 %	98.1 %

Salad number	Main group	Salad name	Manufacturer name	Method of determination	nutriRECIPE-Index aggregated approach	nutriRECIPE-Index disaggregated approach
289	Raw food salads	Salat Kichererbsen mit Buntem Quinoa	Natsu Foods GmbH & Co. KG	Analyses	85.9 %	94.2 %
290	Starch-based salads	Couscoussalat	PUR Bio Feinkost Manufaktur GmbH & Co. KG	No information	78.3 %	82.2 %
291	Starch-based salads	Quinoasalat	Heinrich Kühlmann GmbH & Co. KG	No information	67.4 %	68.3 %
292	Starch-based salads	Bulgursalat	MATISS Feinkost GmbH	No information	82.4 %	53.8 %
293	Starch-based salads	Couscous	Natsu Foods GmbH & Co. KG	Analyses	83.3 %	81.4 %
294	Starch-based salads	Süßkartoffel	Natsu Foods GmbH & Co. KG	Analyses	70.2 %	74.9 %
Total number of analysis-based determination				51		
Total number of literature data-based determination				20		
Total number of combination of both				91		
Total number of calculation and random analysis				47		
Total number of no information				85		
Total number of salads				294		

Table S3. Filled template of the Belgian Federal Public Service with calculated Nutri-Scores and corresponding nutriRECIPE-Index values with both approaches.

Product	Energy *(KJ/100 g or 100 ml)*	Total fat *(g/100 g or 100 ml)*	Saturated fatty acids *(g/100 g or 100 ml)*	Sugars *(g/100 g or 100 ml)*	Proteins *(g/100 g or 100 ml)*	Salt *(g/100 g or 100 ml)*	Fiber *(g/100 g or 100 ml)*	Fruits, vegetables, pulses, nuts, and rapeseed, walnut and olive oils *(%/100 g or 100 ml)*	Sodium *(mg/100 g or 100 ml)*	Points for energy	Points for sugar	Points for saturated fatty acids	Point for sodium	Points for protein	Points for fiber	Points for fruits etc.	Total score	Nutri-Score	nutriRECIPE-Index aggregated approach	nutriRECIPE-Index disaggregated approach
1	430.0	8.1	1.4	2.0	4.6	0.8	1.6	41.0	332.0	1	0	1	3	2	1	1	1	B	115.1 %	110.3 %
2	232.0	2.1	1.1	5.1	2.3	0.7	1.2	61.0	280.0	0	1	1	3	1	1	2	1	B	105.9 %	109.3 %
3	598.0	11.5	1.8	1.9	4.8	1.1	1.5	41.0	452.0	1	0	1	5	2	1	1	3	C	83.5 %	54.7 %
4	363.0	3.1	1.4	7.2	3.2	0.8	1.8	41.0	332.0	1	1	1	3	1	1	1	3	C	89.9 %	69.2 %
5	216.0	1.7	0.2	5.5	1.0	0.6	1.8	81.0	236.0	0	1	0	2	0	1	5	-3	A	82.8 %	82.8 %
6	554.0	9.3	2.6	2.8	4.6	0.8	1.7	61.0	332.0	1	0	2	3	2	1	2	1	B	73.5 %	77.7 %
7	402.0	6.5	1.0	3.4	2.2	0.7	2.5	41.0	268.0	1	0	0	2	1	2	1	-1	A	77.3 %	69.4 %
8	499.0	10.0	3.0	2.0	3.7	1.4	2.4	41.0	556.0	1	0	2	6	2	2	1	4	C	82.4 %	72.9 %
9	408.0	6.1	0.9	2.3	4.0	1.0	1.6	61.0	392.0	1	0	0	4	2	1	2	0	B	77.5 %	75.0 %
10	504.0	4.2	1.2	6.5	4.5	0.9	1.9	39.0	360.0	1	1	1	3	2	2	0	2	B	64.6 %	71.2 %
11	473.0	5.2	0.7	3.5	4.8	1.0	1.0	39.0	408.0	1	0	0	4	2	1	0	2	B	72.0 %	57.4 %
12	445.0	5.3	1.6	4.6	3.9	0.9	1.0	39.0	376.0	1	1	1	4	2	1	0	4	C	68.1 %	76.8 %
13	226.0	2.6	0.2	3.1	1.1	1.0	2.0	81.0	400.0	0	0	0	4	0	2	5	-3	A	89.6 %	89.6 %
14	129.0	0.1	0.0	5.2	1.1	0.8	1.7	81.0	324.0	0	1	0	3	0	1	5	-2	A	88.1 %	88.1 %
15	615.0	7.2	1.7	2.3	6.7	0.8	1.7	39.0	304.0	1	0	1	3	4	1	0	0	B	69.6 %	63.8 %
16	450.0	7.5	2.4	3.2	5.3	0.7	1.7	41.0	292.0	1	0	2	3	3	1	1	1	B	86.5 %	83.6 %
17	690.0	6.3	1.7	7.1	5.9	0.7	2.0	39.0	292.0	2	1	1	3	3	2	0	2	B	44.2 %	58.8 %
18	822.0	13.0	2.5	3.9	4.4	0.3	1.3	39.0	128.0	2	0	2	1	2	1	0	2	B	41.0 %	47.5 %

Product	Energy *(KJ/100 g or 100 ml)*	Total fat *(g/100 g or 100 ml)*	Saturated fatty acids *(g/100 g or 100 ml)*	Sugars *(g/100 g or 100 ml)*	Proteins *(g/100 g or 100 ml)*	Salt *(g/100 g or 100 ml)*	Fiber *(g/100 g or 100 ml)*	Fruits, vegetables, pulses, nuts, and rapeseed, walnut and olive oils *(%/100 g or 100 ml)*	Sodium *(mg/100 g or 100 ml)*	Points for energy	Points for sugar	Points for saturated fatty acids	Point for sodium	Points for protein	Points for fiber	Points for fruits etc.	Total score	Nutri-Score	nutriRECIPE-Index aggregated approach	nutriRECIPE-Index disaggregated approach
19	141.0	0.5	0.1	3.0	1.6	0.1	2.0	81.0	20.0	0	0	0	0	0	2	5	-7	A	124.2 %	124.2 %
20	231.0	0.9	0.3	3.1	4.7	0.5	2.1	81.0	188.0	0	0	0	2	2	2	5	-7	A	107.8 %	104.9 %
21	406.0	7.1	0.6	4.1	3.1	0.6	2.3	61.0	228.0	1	0	0	2	1	2	2	-2	A	102.0 %	96.4 %
22	298.0	4.1	0.4	3.9	1.6	0.6	1.5	61.0	224.0	0	0	0	2	0	1	2	-1	A	78.3 %	74.7 %
23	359.0	5.4	1.2	3.9	3.5	0.8	1.9	61.0	308.0	1	0	1	3	2	2	2	-1	A	90.2 %	74.9 %
24	583.0	12.0	1.3	1.9	3.8	0.1	2.4	81.0	28.0	1	0	1	0	2	2	5	-7	A	94.2 %	90.8 %
25	515.0	9.3	5.1	1.3	7.7	0.8	0.8	41.0	304.0	1	0	5	3	4	0	1	4	C	91.7 %	86.9 %
26	353.0	1.0	0.1	0.6	6.3	0.1	2.7	81.0	24.0	1	0	0	0	3	2	5	-9	A	118.4 %	124.1 %
27	1028.0	19.0	3.6	2.0	4.6	0.7	1.6	39.0	292.0	3	0	3	3	2	1	0	6	C	39.3 %	40.3 %
28	622.0	11.0	1.0	3.8	2.3	0.6	1.9	61.0	252.0	1	0	0	2	1	1	2	-1	A	70.0 %	70.1 %
29	512.0	6.2	1.0	1.4	3.1	0.5	3.0	61.0	192.0	1	0	0	2	1	3	2	-3	A	79.7 %	77.1 %
30	331.0	4.9	0.6	4.1	4.0	0.7	1.2	61.0	260.0	0	0	0	2	2	1	2	-3	A	83.3 %	68.9 %
31	463.0	8.7	0.8	4.9	1.6	0.8	1.4	61.0	332.0	1	1	0	3	0	1	2	2	B	58.0 %	68.3 %
32	458.0	8.7	0.8	4.6	1.8	0.8	2.2	61.0	328.0	1	1	0	3	1	2	2	0	B	75.7 %	75.2 %
33	419.0	8.4	0.7	4.3	1.4	0.5	1.0	61.0	200.0	1	0	0	2	0	1	2	0	B	63.1 %	79.3 %
34	532.0	9.7	1.0	3.9	3.1	0.7	1.7	61.0	284.0	1	0	0	3	1	1	2	0	B	66.6 %	62.8 %
35	296.0	4.1	0.4	4.2	1.5	0.5	1.1	61.0	200.0	0	0	0	2	0	1	2	-1	A	71.1 %	84.1 %
36	514.0	9.5	2.3	3.3	5.1	0.9	1.6	41.0	344.0	1	0	2	3	3	1	1	1	B	77.7 %	65.4 %
37	450.0	7.5	2.4	3.2	5.3	0.7	0.8	39.0	280.0	1	0	2	3	3	0	0	3	C	75.0 %	75.3 %

Product	Energy (KJ/100 g or 100 ml)	Total fat (g/100 g or 100 ml)	Saturated fatty acids (g/100 g or 100 ml)	Sugars (g/100 g or 100 ml)	Proteins (g/100 g or 100 ml)	Salt (g/100 g or 100 ml)	Fiber (g/100 g or 100 ml)	Fruits, vegetables, pulses, nuts, and rapeseed, walnut and olive oils (%/100 g or 100 ml)	Sodium (mg/100 g or 100 ml)	Points for energy	Points for sugar	Points for saturated fatty acids	Point for sodium	Points for protein	Points for fiber	Points for fruits etc.	Total score	Nutri-Score	nutriRECIPE-Index aggregated approach	nutriRECIPE-Index disaggregated approach
38	248.0	3.4	1.4	1.7	4.7	0.3	1.6	61.0	112.0	0	0	1	1	2	1	2	-3	A	140.6 %	143.0 %
39	739.0	11.0	1.9	3.7	4.1	0.5	1.8	39.0	180.0	2	0	1	1	2	1	0	1	B	70.3 %	70.3 %
40	736.0	9.4	2.1	7.0	5.8	1.0	1.7	39.0	388.0	2	1	2	4	3	1	0	5	C	55.1 %	57.6 %
41	377.0	2.1	0.2	3.3	4.6	0.6	2.3	39.0	244.0	1	0	0	2	2	2	0	-1	A	93.3 %	120.5 %
42	544.0	5.4	1.6	2.6	6.9	0.5	1.0	39.0	192.0	1	0	1	2	4	1	0	-1	A	54.8 %	67.0 %
43	528.0	5.8	0.5	6.7	3.3	0.7	0.9	39.0	276.0	1	1	0	3	2	0	0	3	C	47.3 %	97.6 %
44	723.0	12.0	3.1	4.1	5.5	0.9	1.1	41.0	348.0	2	0	3	3	3	1	1	3	C	50.8 %	51.1 %
45	364.0	2.8	1.3	8.9	3.5	0.5	2.8	61.0	204.0	1	1	1	2	2	3	2	-2	A	84.8 %	90.7 %
46	400.0	6.8	2.0	4.1	3.0	0.9	2.5	61.0	348.0	1	0	1	3	1	2	2	0	B	84.7 %	55.1 %
47	450.0	7.2	2.8	3.2	6.0	0.5	4.0	41.0	184.0	1	0	2	2	3	4	1	-3	A	96.8 %	108.4 %
48	494.0	6.8	2.0	3.5	4.5	0.6	1.4	41.0	236.0	1	0	1	2	2	1	1	0	B	75.1 %	80.3 %
49	521.0	6.5	1.8	5.1	5.1	1.9	3.8	61.0	760.0	1	1	1	8	3	4	2	5	C	68.5 %	68.6 %
50	442.0	3.0	0.3	13.0	1.1	1.4	1.9	61.0	560.0	1	2	0	6	0	1	2	6	C	57.1 %	57.1 %
51	244.0	1.1	0.1	10.0	0.5	1.3	0.7	61.0	520.0	0	2	0	5	0	0	2	5	C	9.0 %	9.0 %
52	360.0	2.2	0.2	14.0	0.6	1.0	2.2	61.0	400.0	1	3	0	4	0	2	2	4	C	26.4 %	26.4 %
53	273.0	2.6	0.2	8.7	0.8	0.9	1.3	61.0	360.0	0	1	0	3	0	1	2	1	B	48.8 %	48.8 %
54	619.0	11.0	0.9	9.0	1.1	1.5	2.6	61.0	600.0	1	1	0	6	0	2	2	4	C	48.9 %	48.9 %
55	964.0	20.0	1.7	7.0	2.0	0.8	2.6	41.0	320.0	2	1	1	3	1	2	1	3	C	45.8 %	45.8 %
56	1028.0	19.0	3.6	2.0	4.6	0.7	1.5	39.0	292.0	3	0	3	3	2	1	0	6	C	41.9 %	40.3 %

Product	Energy (KJ/100 g or 100 ml)	Total fat (g/100 g or 100 ml)	Saturated fatty acids (g/100 g or 100 ml)	Sugars (g/100 g or 100 ml)	Proteins (g/100 g or 100 ml)	Salt (g/100 g or 100 ml)	Fiber (g/100 g or 100 ml)	Fruits, vegetables, pulses, nuts, and rapeseed, walnut and olive oils (%/100 g or 100 ml)	Sodium (mg/100 g or 100 ml)	Points for energy	Points for sugar	Points for saturated fatty acids	Point for sodium	Points for protein	Points for fiber	Points for fruits etc.	Total score	Nutri-Score	nutriRECIPE-Index aggregated approach	nutriRECIPE-Index disaggregated approach
57	584.0	8.8	1.2	4.4	4.3	0.5	1.0	39.0	212.0	1	0	1	2	2	1	0	1	B	62.0 %	55.8 %
58	563.0	8.5	0.8	3.8	4.0	0.8	2.9	39.0	300.0	1	0	0	3	2	3	0	-1	A	92.6 %	90.1 %
59	418.0	2.9	1.1	8.3	2.3	0.6	1.9	39.0	248.0	1	1	1	2	1	1	0	3	C	42.4 %	58.3 %
60	545.0	8.6	2.0	3.5	4.4	1.1	1.2	41.0	440.0	1	0	1	4	2	1	1	2	B	87.2 %	95.2 %
61	374.0	5.9	0.5	4.3	1.3	0.7	1.3	61.0	296.0	1	0	0	3	0	1	2	1	B	60.0 %	80.1 %
62	595.0	9.2	2.6	7.8	5.5	0.9	0.7	41.0	352.0	1	1	2	3	3	0	1	3	C	51.6 %	42.7 %
63	396.0	3.9	1.2	5.0	3.3	1.1	2.5	41.0	440.0	1	1	1	4	2	2	1	2	B	73.4 %	73.6 %
64	668.0	8.5	1.7	8.8	4.4	0.8	1.7	39.0	332.0	1	1	1	3	2	1	0	3	C	62.3 %	57.2 %
65	320.0	4.9	0.8	3.9	3.0	0.9	0.9	41.0	356.0	0	0	0	3	1	0	1	1	B	60.0 %	63.8 %
66	380.0	3.3	0.4	1.8	6.6	0.6	1.8	61.0	224.0	1	0	0	2	4	1	2	-4	A	93.3 %	105.0 %
67	278.0	2.8	0.7	6.6	1.3	0.6	4.0	81.0	224.0	0	1	0	2	0	4	5	-6	A	64.0 %	39.3 %
68	278.0	2.8	0.7	6.6	1.3	0.6	0.9	81.0	224.0	0	1	0	2	0	1	5	-3	A	97.4 %	99.2 %
69	278.0	2.8	0.7	6.6	1.3	0.6	1.5	61.0	224.0	0	1	0	2	0	1	2	0	B	89.5 %	81.2 %
70	429.0	8.2	0.8	4.2	1.7	1.5	1.4	41.0	612.0	1	0	0	6	1	1	1	4	C	57.6 %	54.1 %
71	429.0	8.2	0.8	4.2	1.7	1.5	1.5	61.0	612.0	1	0	0	6	1	1	2	3	C	61.5 %	64.7 %
72	440.0	8.4	1.1	3.7	1.8	0.6	1.0	61.0	232.0	1	0	1	2	1	1	2	0	B	58.6 %	62.8 %
73	398.0	8.2	0.8	3.2	1.5	0.6	1.1	61.0	224.0	1	0	0	2	0	1	2	0	B	65.8 %	69.1 %
74	436.0	8.3	0.8	3.5	1.8	0.6	1.2	61.0	236.0	1	0	0	2	1	1	2	-1	A	65.9 %	70.3 %
75	110.0	0.3	0.1	1.7	1.5	0.1	2.0	81.0	40.0	0	0	0	0	0	2	5	-7	A	134.2 %	134.2 %

Product	Energy (KJ/100 g or 100 ml)	Total fat (g/100 g or 100 ml)	Saturated fatty acids (g/100 g or 100 ml)	Sugars (g/100 g or 100 ml)	Proteins (g/100 g or 100 ml)	Salt (g/100 g or 100 ml)	Fiber (g/100 g or 100 ml)	Fruits, vegetables, pulses, nuts, and rapeseed, walnut and olive oils (%/100 g or 100 ml)	Sodium (mg/100 g or 100 ml)	Points for energy	Points for sugar	Points for saturated fatty acids	Point for sodium	Points for protein	Points for fiber	Points for fruits etc.	Total score	Nutri-Score	nutriRECIPE-Index aggregated approach	nutriRECIPE-Index disaggregated approach
76	202.0	1.8	0.3	6.4	1.0	0.4	1.7	61.0	156.0	0	1	0	1	0	1	2	-1	A	97.6 %	91.5 %
77	654.0	11.3	2.2	4.3	4.3	0.9	1.3	41.0	340.0	1	0	2	3	2	1	1	2	B	57.8 %	71.9 %
78	577.0	10.8	0.7	3.3	5.2	0.5	1.7	41.0	200.0	1	0	0	2	3	1	1	-2	A	88.0 %	79.9 %
79	400.0	5.6	1.0	1.0	2.3	0.8	2.8	81.0	320.0	1	0	0	3	1	3	5	-5	A	78.3 %	98.1 %
80	1444.0	21.8	3.1	6.4	8.2	0.7	2.4	39.0	284.0	4	1	3	3	5	2	0	9	C	44.3 %	44.1 %
81	605.0	13.7	2.0	2.1	2.9	0.8	1.0	41.0	328.0	1	0	1	3	1	1	1	2	B	56.0 %	65.3 %
82	596.0	10.1	3.0	0.9	7.8	0.8	1.1	39.0	308.0	1	0	2	3	4	1	0	1	B	84.7 %	74.7 %
83	278.0	2.8	0.7	6.6	1.3	0.6	2.7	81.0	224.0	0	1	0	2	0	2	5	-4	A	85.7 %	85.7 %
84	272.0	2.4	0.8	7.9	0.7	1.3	1.4	61.0	516.0	0	1	0	5	0	1	2	3	C	27.1 %	27.1 %
85	246.0	3.6	1.2	3.6	2.3	0.4	1.0	81.0	176.0	0	0	1	1	1	1	5	-5	A	91.1 %	86.0 %
86	194.0	1.8	0.7	5.1	1.9	0.3	2.0	81.0	116.0	0	1	0	1	1	2	5	-6	A	96.7 %	94.2 %
87	378.0	5.0	0.4	9.0	0.8	0.8	2.2	81.0	328.0	1	1	0	3	0	2	5	-2	A	79.2 %	78.8 %
88	380.0	2.5	0.3	3.3	3.9	0.6	1.5	39.0	248.0	1	0	0	2	2	1	0	0	B	80.0 %	80.9 %
89	721.0	9.1	2.0	8.1	5.4	0.9	1.6	39.0	352.0	2	1	1	3	3	1	0	3	C	44.3 %	49.8 %
90	482.0	8.3	3.3	2.0	6.1	1.0	0.9	41.0	396.0	1	0	3	4	3	1	1	3	C	71.1 %	76.1 %
91	429.0	7.8	0.7	4.8	1.3	0.4	2.1	61.0	152.0	1	1	0	1	0	2	2	-1	A	75.1 %	75.6 %
92	415.0	3.8	1.5	6.6	4.2	0.6	1.9	41.0	228.0	1	1	1	2	2	1	1	1	B	76.1 %	61.8 %
93	447.0	7.2	0.6	4.0	3.8	0.5	1.2	61.0	216.0	1	0	0	2	2	1	2	-2	A	66.0 %	82.0 %
94	545.0	7.7	2.5	4.6	4.9	1.0	2.3	41.0	416.0	1	1	2	4	3	2	1	2	B	82.9 %	72.2 %

Product	Energy (KJ/100 g or 100 ml)	Total fat (g/100 g or 100 ml)	Saturated fatty acids (g/100 g or 100 ml)	Sugars (g/100 g or 100 ml)	Proteins (g/100 g or 100 ml)	Salt (g/100 g or 100 ml)	Fiber (g/100 g or 100 ml)	Fruits, vegetables, pulses, nuts, and rapeseed, walnut and olive oils (%/100 g or 100 ml)	Sodium (mg/100 g or 100 ml)	Points for energy	Points for sugar	Points for saturated fatty acids	Point for sodium	Points for protein	Points for fiber	Points for fruits etc.	Total score	Nutri-Score	nutriRECIPE-Index aggregated approach	nutriRECIPE-Index disaggregated approach
95	378.0	3.3	0.3	11.2	1.0	1.1	2.3	81.0	452.0	1	2	0	5	0	2	5	1	B	50.2 %	50.2 %
96	193.0	1.0	0.1	8.3	0.5	1.6	0.7	61.0	632.0	0	1	0	7	0	0	2	6	C	24.1 %	24.1 %
97	269.0	4.2	0.4	3.1	1.1	1.2	1.2	61.0	480.0	0	0	0	5	0	1	2	2	B	66.4 %	62.5 %
98	550.0	10.1	2.3	5.1	3.7	0.7	1.6	41.0	296.0	1	1	2	3	2	1	1	3	C	70.6 %	70.1 %
99	654.0	11.3	2.2	4.3	4.3	0.9	1.3	41.0	340.0	1	0	2	3	2	1	1	2	B	57.1 %	71.6 %
100	470.0	6.4	0.6	7.6	3.9	0.7	1.2	41.0	288.0	1	1	0	3	2	1	1	1	B	53.6 %	50.0 %
101	319.0	4.2	1.9	1.4	7.4	0.4	1.5	41.0	160.0	0	0	1	1	4	1	1	-4	A	124.8 %	115.1 %
102	372.0	2.5	1.5	2.1	4.6	0.2	2.7	41.0	80.0	1	0	1	0	2	2	1	-3	A	106.0 %	96.8 %
103	321.0	3.2	1.7	1.6	7.6	0.5	1.7	61.0	200.0	0	0	1	2	4	1	2	-4	A	116.4 %	90.7 %
104	135.0	0.4	0.1	3.0	1.8	0.2	2.1	81.0	80.0	0	0	0	0	1	2	5	-8	A	122.2 %	112.0 %
105	201.0	0.7	0.2	2.7	4.7	0.4	2.3	81.0	160.0	0	0	0	1	2	2	5	-8	A	128.1 %	120.7 %
106	240.0	1.8	0.5	2.2	5.3	0.3	1.8	81.0	120.0	0	0	0	1	3	1	5	-8	A	143.2 %	154.7 %
107	116.0	0.5	0.1	4.3	1.6	0.1	3.0	81.0	32.0	0	0	0	0	0	3	5	-8	A	113.8 %	113.8 %
108	76.0	0.2	0.1	0.8	1.6	0.1	1.9	81.0	40.0	0	0	0	0	0	2	5	-7	A	139.8 %	139.8 %
109	76.0	0.2	0.1	0.8	1.6	0.1	1.9	81.0	40.0	0	0	0	0	0	2	5	-7	A	139.3 %	139.8 %
110	441.0	5.4	0.5	7.8	4.5	0.7	1.5	41.0	280.0	1	1	0	3	2	1	1	1	B	75.9 %	74.8 %
111	740.0	9.2	1.9	7.3	5.5	1.0	1.6	39.0	384.0	2	1	1	4	3	1	0	4	C	51.7 %	57.7 %
112	524.0	4.9	1.4	5.9	4.2	0.5	1.5	39.0	204.0	1	1	1	2	2	1	0	2	B	68.6 %	60.7 %
113	482.0	7.4	2.0	2.9	5.3	1.1	2.2	61.0	440.0	1	0	1	4	3	2	2	-1	A	93.7 %	84.1 %

Product	Energy (KJ/100 g or 100 ml)	Total fat (g/100 g or 100 ml)	Saturated fatty acids (g/100 g or 100 ml)	Sugars (g/100 g or 100 ml)	Proteins (g/100 g or 100 ml)	Salt (g/100 g or 100 ml)	Fiber (g/100 g or 100 ml)	Fruits, vegetables, pulses, nuts, and rapeseed, walnut and olive oils (%/100 g or 100 ml)	Sodium (mg/100 g or 100 ml)	Points for energy	Points for sugar	Points for saturated fatty acids	Point for sodium	Points for protein	Points for fiber	Points for fruits etc.	Total score	Nutri-Score	nutriRECIPE-Index aggregated approach	nutriRECIPE-Index disaggregated approach
114	589.0	7.1	1.8	2.0	7.2	0.7	1.0	39.0	288.0	1	0	1	3	4	1	0	0	B	56.4 %	59.7 %
115	383.0	3.3	0.2	12.7	1.0	1.4	2.3	61.0	560.0	1	2	0	6	0	2	2	5	C	71.5 %	71.5 %
116	313.0	4.0	1.8	3.4	5.4	0.7	0.9	61.0	260.0	0	0	1	2	3	0	2	-2	A	79.8 %	78.9 %
117	284.0	3.2	0.5	6.3	1.3	2.0	8.7	81.0	800.0	0	1	0	8	0	5	5	-1	A	69.6 %	83.7 %
118	403.0	3.6	0.5	12.3	0.8	0.8	2.5	61.0	312.0	1	2	0	3	0	2	2	2	B	53.5 %	53.5 %
119	377.0	3.4	0.3	12.0	0.9	1.6	2.3	61.0	640.0	1	2	0	7	0	2	2	6	C	41.3 %	41.3 %
120	392.0	4.0	0.6	13.0	1.0	1.7	2.2	61.0	680.0	1	2	0	7	0	2	2	6	C	37.7 %	37.7 %
121	448.0	5.0	0.4	12.3	1.0	1.3	2.2	61.0	500.0	1	2	0	5	0	2	2	4	C	50.8 %	50.8 %
122	874.0	17.0	2.0	11.0	1.0	1.3	1.8	41.0	520.0	2	2	1	5	0	1	1	8	C	29.1 %	29.1 %
123	448.0	5.0	0.4	12.3	1.0	1.3	2.2	61.0	500.0	1	2	0	5	0	2	2	4	C	50.8 %	50.8 %
124	545.0	10.4	0.9	7.0	0.8	0.8	0.9	61.0	320.0	1	1	0	3	0	0	2	3	C	26.3 %	26.3 %
125	606.0	11.7	3.2	8.1	1.3	0.9	1.8	41.0	344.0	1	1	3	3	0	1	1	6	C	20.2 %	20.2 %
126	434.0	5.0	0.4	11.0	1.0	1.2	2.3	61.0	480.0	1	2	0	5	0	2	2	4	C	69.9 %	69.9 %
127	389.0	3.0	0.2	13.0	0.7	0.8	2.2	61.0	320.0	1	2	0	3	0	2	2	2	B	30.1 %	30.1 %
128	448.0	5.0	0.4	12.3	1.0	1.3	2.2	61.0	500.0	1	2	0	5	0	2	2	4	C	63.6 %	63.6 %
129	409.0	6.5	0.9	4.7	3.5	0.8	2.3	61.0	324.0	1	1	0	3	2	2	2	-1	A	68.7 %	69.9 %
130	537.0	9.5	2.7	3.1	5.6	0.9	1.9	61.0	372.0	1	0	2	4	3	2	2	0	B	79.9 %	85.1 %
131	175.0	0.4	0.1	5.6	1.2	0.6	2.0	81.0	224.0	0	1	0	2	0	2	5	-4	A	92.3 %	92.3 %
132	492.0	4.7	0.8	1.0	5.3	1.1	1.4	39.0	432.0	1	0	0	4	3	1	0	1	B	66.4 %	49.2 %

Product	Energy (KJ/100 g or 100 ml)	Total fat (g/100 g or 100 ml)	Saturated fatty acids (g/100 g or 100 ml)	Sugars (g/100 g or 100 ml)	Proteins (g/100 g or 100 ml)	Salt (g/100 g or 100 ml)	Fiber (g/100 g or 100 ml)	Fruits, vegetables, pulses, nuts, and rapeseed, walnut and olive oils (%/100 g or 100 ml)	Sodium (mg/100 g or 100 ml)	Points for energy	Points for sugar	Points for saturated fatty acids	Point for sodium	Points for protein	Points for fiber	Points for fruits etc.	Total score	Nutri-Score	nutriRECIPE-Index aggregated approach	nutriRECIPE-Index disaggregated approach
133	489.0	5.0	1.2	3.1	4.4	1.1	0.8	39.0	440.0	1	0	1	4	2	0	0	4	C	50.3 %	30.7 %
134	495.0	5.9	1.4	1.1	7.4	0.9	1.1	39.0	372.0	1	0	1	4	4	1	0	1	B	71.7 %	52.1 %
135	603.0	14.0	1.8	2.0	1.0	0.2	0.6	61.0	96.0	1	0	1	1	0	0	2	1	B	33.5 %	54.6 %
136	431.0	5.2	0.4	12.0	0.7	1.1	2.5	61.0	440.0	1	2	0	4	0	2	2	3	C	29.5 %	24.2 %
137	353.0	3.6	0.5	11.3	0.8	1.3	2.1	61.0	520.0	1	2	0	5	0	2	2	4	C	62.8 %	62.8 %
138	394.0	6.4	0.7	3.6	1.3	0.8	1.8	41.0	320.0	1	0	0	3	0	1	1	2	B	73.4 %	63.3 %
139	671.0	13.0	5.0	3.0	8.0	2.3	0.9	39.0	900.0	2	0	4	9	4	1	0	14	D	55.4 %	54.9 %
140	742.0	14.0	6.5	3.0	10.0	2.3	0.2	39.0	900.0	2	0	6	9	5	0	0	17	D	59.0 %	57.0 %
141	717.0	5.9	0.5	3.4	3.1	2.1	3.6	39.0	840.0	2	0	0	9	1	3	0	8	C	64.0 %	86.9 %
142	877.0	7.4	0.7	7.1	3.4	1.2	2.8	39.0	480.0	2	1	0	5	2	3	0	3	C	41.2 %	36.5 %
143	677.0	5.6	0.5	4.7	3.1	1.1	3.7	39.0	428.0	2	1	0	4	1	4	0	2	B	55.1 %	71.9 %
144	753.0	14.7	1.4	8.3	1.0	1.3	3.1	61.0	532.0	2	1	1	5	0	3	2	4	C	46.5 %	46.5 %
145	984.0	14.9	2.1	7.0	3.7	1.4	2.8	39.0	552.0	2	1	2	6	2	3	0	8	C	30.0 %	29.9 %
146	478.0	8.4	0.6	7.2	0.6	0.7	3.1	61.0	260.0	1	1	0	2	0	3	2	-1	A	65.1 %	65.1 %
147	863.0	16.0	1.8	4.1	3.3	1.5	2.9	39.0	588.0	2	0	1	6	2	3	0	4	C	52.6 %	28.9 %
148	555.0	7.1	0.7	5.3	1.6	1.0	1.0	39.0	396.0	1	1	0	4	0	1	0	5	C	41.8 %	41.8 %
149	648.0	13.0	1.0	7.0	1.1	0.7	2.3	61.0	260.0	1	1	0	2	0	2	2	0	B	48.1 %	48.1 %
150	593.0	8.6	0.9	4.8	2.3	1.5	1.2	39.0	600.0	1	1	0	6	1	1	0	6	C	43.3 %	43.3 %
151	528.0	6.4	1.0	4.3	1.4	1.3	1.5	39.0	520.0	1	0	0	5	0	1	0	5	C	41.3 %	39.9 %

Product	Energy (KJ/100 g or 100 ml)	Total fat (g/100 g or 100 ml)	Saturated fatty acids (g/100 g or 100 ml)	Sugars (g/100 g or 100 ml)	Proteins (g/100 g or 100 ml)	Salt (g/100 g or 100 ml)	Fiber (g/100 g or 100 ml)	Fruits, vegetables, pulses, nuts, and rapeseed, walnut and olive oils (%/100 g or 100 ml)	Sodium (mg/100 g or 100 ml)	Points for energy	Points for sugar	Points for saturated fatty acids	Point for sodium	Points for protein	Points for fiber	Points for fruits etc.	Total score	Nutri-Score	nutriRECIPE-Index aggregated approach	nutriRECIPE-Index disaggregated approach
152	591.0	8.8	1.5	3.5	1.2	1.3	1.5	39.0	520.0	1	0	1	5	0	1	0	6	C	39.1 %	47.1 %
153	1432.0	32.8	4.9	6.4	4.4	1.4	0.2	39.0	560.0	4	1	4	6	2	0	0	15	D	26.8 %	28.3 %
154	936.0	18.9	3.2	5.8	3.7	2.3	0.9	41.0	912.0	2	1	3	10	2	0	1	15	D	27.0 %	34.0 %
155	792.0	12.7	1.2	8.0	9.6	1.1	1.9	39.0	452.0	2	1	1	5	5	2	0	2	B	46.5 %	46.5 %
156	883.0	12.1	1.6	5.4	4.5	1.5	1.2	39.0	588.0	2	1	1	6	2	1	0	7	C	19.6 %	20.6 %
157	1463.0	34.5	4.4	4.5	4.9	1.5	0.2	39.0	608.0	4	0	4	6	3	0	0	14	D	33.7 %	33.8 %
158	1189.0	26.8	3.8	3.7	5.3	1.9	0.7	39.0	752.0	3	0	3	8	3	0	0	14	D	36.5 %	34.0 %
159	1200.0	26.6	3.8	4.6	6.8	1.9	0.2	39.0	760.0	3	1	3	8	4	0	0	15	D	37.7 %	37.7 %
160	961.0	19.8	2.0	3.6	7.2	1.8	1.6	39.0	700.0	2	0	1	7	4	1	0	5	C	54.2 %	54.2 %
161	744.0	14.9	1.3	7.0	2.6	1.5	3.2	39.0	588.0	2	1	1	6	1	3	0	6	C	45.2 %	45.5 %
162	658.0	10.0	0.9	3.3	2.3	0.9	1.5	39.0	372.0	1	0	0	4	1	1	0	3	C	50.8 %	50.8 %
163	875.0	11.0	0.9	2.3	8.3	0.9	3.8	41.0	360.0	2	0	0	3	5	4	1	-5	A	81.8 %	88.6 %
164	636.0	8.0	0.7	4.6	1.6	1.3	1.7	39.0	520.0	1	1	0	5	0	1	0	6	C	44.9 %	44.9 %
165	616.0	8.1	0.6	6.1	1.4	1.2	1.3	39.0	480.0	1	1	0	5	0	1	0	6	C	37.2 %	44.5 %
166	1010.0	19.4	2.8	3.2	4.3	1.5	0.6	39.0	612.0	3	0	2	6	2	0	0	11	D	24.0 %	26.2 %
167	640.0	9.7	1.0	3.7	2.4	1.5	0.8	39.0	588.0	1	0	0	6	1	0	0	6	C	41.3 %	41.3 %
168	930.0	16.2	1.9	3.5	3.0	1.7	1.2	39.0	660.0	2	0	1	7	1	1	0	8	C	27.6 %	51.4 %
169	543.0	8.6	2.0	3.5	4.4	1.1	3.6	41.0	440.0	1	0	1	4	2	3	1	0	B	95.8 %	95.2 %
170	592.0	9.2	2.6	7.8	5.5	0.9	0.6	39.0	352.0	1	1	2	3	3	0	0	4	C	49.7 %	44.3 %

Product	Energy (KJ/100 g or 100 ml)	Total fat (g/100 g or 100 ml)	Saturated fatty acids (g/100 g or 100 ml)	Sugars (g/100 g or 100 ml)	Proteins (g/100 g or 100 ml)	Salt (g/100 g or 100 ml)	Fiber (g/100 g or 100 ml)	Fruits, vegetables, pulses, nuts, and rapeseed, walnut and olive oils (%/100 g or 100 ml)	Sodium (mg/100 g or 100 ml)	Points for energy	Points for sugar	Points for saturated fatty acids	Point for sodium	Points for protein	Points for fiber	Points for fruits etc.	Total score	Nutri-Score	nutriRECIPE-Index aggregated approach	nutriRECIPE-Index disaggregated approach
171	919.0	18.0	2.4	3.9	2.4	0.9	0.8	39.0	340.0	2	0	2	3	1	0	0	6	C	44.6 %	48.1 %
172	810.0	14.0	1.5	5.0	2.0	0.9	1.6	39.0	360.0	2	1	1	3	1	1	0	5	C	39.8 %	39.8 %
173	913.0	16.0	2.3	5.3	4.1	1.6	1.4	39.0	640.0	2	1	2	7	2	1	0	11	D	30.9 %	27.4 %
174	713.0	11.0	1.0	4.0	2.0	1.1	1.4	39.0	440.0	2	0	0	4	1	1	0	4	C	42.5 %	43.0 %
175	1129.0	25.0	4.7	4.0	3.9	1.5	1.3	39.0	580.0	3	0	4	6	2	1	0	12	D	30.6 %	30.5 %
176	647.0	9.9	0.8	3.6	1.5	1.1	1.0	39.0	444.0	1	0	0	4	0	1	0	4	C	42.5 %	42.5 %
177	898.0	8.2	0.7	4.3	4.0	1.4	2.7	39.0	540.0	2	0	0	5	2	2	0	3	C	51.8 %	48.3 %
178	727.0	5.7	0.5	5.0	3.3	1.3	4.6	39.0	512.0	2	1	0	5	2	4	0	2	B	74.8 %	105.7 %
179	750.0	6.5	0.5	3.1	3.9	1.4	4.4	39.0	552.0	2	0	0	6	2	4	0	2	B	69.9 %	84.7 %
180	852.0	11.2	1.0	4.9	5.9	1.4	3.4	39.0	568.0	2	1	0	6	3	3	0	3	C	64.6 %	62.2 %
181	750.0	8.4	0.8	6.0	3.5	1.3	3.1	41.0	520.0	2	1	0	5	2	3	1	2	B	65.5 %	63.9 %
182	646.0	6.2	0.7	4.6	3.9	1.4	2.1	39.0	568.0	1	1	0	6	2	2	0	4	C	86.4 %	85.1 %
183	805.0	14.0	2.7	3.3	4.3	1.4	1.3	39.0	560.0	2	0	2	6	2	1	0	7	C	64.0 %	69.7 %
184	676.0	13.0	5.0	3.0	8.0	2.3	1.0	39.0	920.0	2	0	4	10	4	1	0	15	D	56.2 %	52.1 %
185	828.0	11.0	1.1	5.3	5.1	1.1	3.7	39.0	440.0	2	1	1	4	3	4	0	1	B	61.7 %	72.9 %
186	660.0	5.6	1.3	7.8	4.1	1.3	3.1	41.0	520.0	1	1	1	5	2	3	1	2	B	49.5 %	64.1 %
187	708.0	13.0	1.2	9.4	2.6	1.3	2.0	61.0	520.0	2	2	1	5	1	2	2	5	C	41.6 %	25.4 %
188	873.0	12.0	1.1	2.9	5.6	0.9	2.0	39.0	372.0	2	0	1	4	3	2	0	2	B	55.2 %	68.9 %
189	443.0	5.3	0.7	10.5	0.8	1.6	1.9	61.0	640.0	1	2	0	7	0	1	2	7	C	39.7 %	39.7 %

Product	Energy (KJ/100 g or 100 ml)	Total fat (g/100 g or 100 ml)	Saturated fatty acids (g/100 g or 100 ml)	Sugars (g/100 g or 100 ml)	Proteins (g/100 g or 100 ml)	Salt (g/100 g or 100 ml)	Fiber (g/100 g or 100 ml)	Fruits, vegetables, pulses, nuts, and rapeseed, walnut and olive oils (%/100 g or 100 ml)	Sodium (mg/100 g or 100 ml)	Points for energy	Points for sugar	Points for saturated fatty acids	Point for sodium	Points for protein	Points for fiber	Points for fruits etc.	Total score	Nutri-Score	nutriRECIPE-Index aggregated approach	nutriRECIPE-Index disaggregated approach
190	1023.0	21.0	1.9	6.8	6.1	1.1	2.6	39.0	440.0	3	1	1	4	3	2	0	4	C	36.9 %	36.9 %
191	751.0	7.7	0.6	3.1	3.2	1.2	5.4	39.0	480.0	2	0	0	5	1	5	0	1	B	81.5 %	74.0 %
192	728.0	5.9	0.5	3.5	3.1	0.8	3.7	39.0	320.0	2	0	0	3	1	3	0	1	B	70.3 %	73.9 %
193	1220.0	26.7	3.0	5.0	6.8	1.4	0.5	39.0	560.0	3	1	2	6	4	0	0	12	D	23.9 %	30.3 %
194	491.0	5.7	0.4	4.7	1.5	1.7	1.5	39.0	680.0	1	1	0	7	0	1	0	8	C	40.4 %	39.3 %
195	714.0	13.8	1.2	8.3	0.7	1.0	3.1	41.0	400.0	2	1	1	4	0	3	1	4	C	31.7 %	41.9 %
196	769.0	11.0	1.8	2.0	3.4	3.1	2.8	39.0	1240.0	2	0	1	10	2	2	0	11	D	82.3 %	82.2 %
197	1244.0	27.4	2.7	3.6	9.3	0.9	0.4	39.0	340.0	3	0	2	3	5	0	0	3	C	83.7 %	83.7 %
198	1196.0	26.0	2.2	9.9	1.1	1.0	2.6	41.0	380.0	3	2	2	4	0	2	1	8	C	35.6 %	35.6 %
199	521.0	5.0	0.4	4.4	2.1	1.4	2.3	39.0	552.0	1	0	0	6	1	2	0	4	C	77.3 %	77.3 %
200	865.0	14.2	1.4	6.0	3.0	1.6	1.1	39.0	632.0	2	1	1	7	1	1	0	10	C	57.5 %	57.5 %
201	949.0	15.8	1.6	5.2	3.1	0.9	3.0	39.0	352.0	2	1	1	3	1	3	0	3	C	55.1 %	67.9 %
202	930.0	17.6	1.8	5.4	10.2	1.1	0.4	39.0	440.0	2	1	1	4	5	0	0	3	C	40.6 %	41.0 %
203	468.0	5.3	0.3	3.7	1.6	1.5	0.9	39.0	600.0	1	0	0	6	0	0	0	7	C	40.5 %	58.6 %
204	629.0	9.0	1.0	3.7	2.8	1.4	1.0	39.0	540.0	1	0	0	5	1	1	0	4	C	50.7 %	50.7 %
205	480.0	5.3	0.9	4.6	2.6	1.8	1.5	39.0	728.0	1	1	0	8	1	1	0	8	C	45.7 %	34.9 %
206	551.0	8.0	1.6	0.8	2.2	1.0	1.7	39.0	400.0	1	0	1	4	1	1	0	4	C	50.7 %	51.5 %
207	921.0	16.4	1.7	6.4	3.0	1.7	1.0	39.0	680.0	2	1	1	7	1	1	0	10	C	49.2 %	49.2 %
208	231.0	0.9	0.3	3.1	4.7	0.5	2.1	81.0	188.0	0	0	0	2	2	2	5	-7	A	107.8 %	104.9 %

Product	Energy (KJ/100 g or 100 ml)	Total fat (g/100 g or 100 ml)	Saturated fatty acids (g/100 g or 100 ml)	Sugars (g/100 g or 100 ml)	Proteins (g/100 g or 100 ml)	Salt (g/100 g or 100 ml)	Fiber (g/100 g or 100 ml)	Fruits, vegetables, pulses, nuts, and rapeseed, walnut and olive oils (%/100 g or 100 ml)	Sodium (mg/100 g or 100 ml)	Points for energy	Points for sugar	Points for saturated fatty acids	Point for sodium	Points for protein	Points for fiber	Points for fruits etc.	Total score	Nutri-Score	nutriRECIPE-Index aggregated approach	nutriRECIPE-Index disaggregated approach
209	1130.0	17.0	2.0	3.9	5.1	2.0	2.7	41.0	800.0	3	0	1	8	3	2	1	9	C	67.7 %	87.6 %
210	572.0	8.0	1.3	10.0	2.9	2.8	2.2	61.0	1120.0	1	2	1	10	1	2	2	10	C	87.8 %	90.3 %
211	655.0	3.7	0.5	4.7	8.8	1.9	4.5	41.0	768.0	1	1	0	8	5	4	1	0	B	91.8 %	102.2 %
212	553.0	3.0	0.3	2.9	3.9	0.8	4.1	39.0	328.0	1	0	0	3	2	4	0	-2	A	107.6 %	76.0 %
213	937.0	8.1	0.7	5.3	4.6	1.2	2.4	39.0	492.0	2	1	0	5	2	2	0	4	C	54.2 %	50.2 %
214	991.0	19.0	2.1	3.6	3.0	1.5	1.1	39.0	600.0	2	0	2	6	1	1	0	8	C	30.0 %	31.8 %
215	974.0	22.8	1.9	5.1	1.0	1.1	2.5	61.0	440.0	2	1	1	4	0	2	2	4	C	38.1 %	38.4 %
216	908.0	20.6	1.6	7.5	0.6	1.3	1.7	41.0	520.0	2	1	1	5	0	1	1	7	C	30.5 %	30.5 %
217	1148.0	24.6	3.3	4.6	7.9	1.0	1.3	39.0	400.0	3	1	3	4	4	1	0	10	C	68.9 %	68.9 %
218	682.0	6.1	1.0	2.7	6.2	1.1	2.4	39.0	440.0	2	0	0	4	3	2	0	1	B	66.7 %	48.7 %
219	405.0	3.2	0.3	3.4	1.2	1.1	4.1	41.0	440.0	1	0	0	4	0	4	1	0	B	101.2 %	118.1 %
220	822.0	7.6	2.5	7.3	6.2	1.1	1.3	39.0	440.0	2	1	2	4	3	1	0	5	C	66.8 %	68.8 %
221	882.0	9.0	1.1	5.2	5.3	1.2	3.3	39.0	480.0	2	1	1	5	3	3	0	3	C	78.5 %	57.9 %
222	879.0	13.0	1.2	3.3	5.8	1.2	3.1	39.0	480.0	2	0	1	5	3	3	0	2	B	86.4 %	73.9 %
223	554.0	4.7	0.6	7.3	3.2	1.0	2.6	41.0	400.0	1	1	0	4	1	2	1	2	B	72.3 %	70.5 %
224	1028.0	19.0	3.6	2.0	4.6	0.7	1.5	39.0	292.0	3	0	3	3	2	1	0	6	C	42.7 %	40.3 %
225	437.0	3.0	1.6	4.4	4.7	0.8	1.2	39.0	316.0	1	0	1	3	2	1	0	2	B	57.9 %	64.4 %
226	374.0	5.9	0.5	4.3	1.3	0.7	2.6	61.0	296.0	1	0	0	3	0	2	2	0	B	82.2 %	78.8 %
227	1219.0	26.3	3.0	4.6	7.1	1.9	0.3	39.0	740.0	3	1	2	8	4	0	0	14	D	43.9 %	55.1 %

Product	Energy (KJ/100 g or 100 ml)	Total fat (g/100 g or 100 ml)	Saturated fatty acids (g/100 g or 100 ml)	Sugars (g/100 g or 100 ml)	Proteins (g/100 g or 100 ml)	Salt (g/100 g or 100 ml)	Fiber (g/100 g or 100 ml)	Fruits, vegetables, pulses, nuts, and rapeseed, walnut and olive oils (%/100 g or 100 ml)	Sodium (mg/100 g or 100 ml)	Points for energy	Points for sugar	Points for saturated fatty acids	Point for sodium	Points for protein	Points for fiber	Points for fruits etc.	Total score	Nutri-Score	nutriRECIPE-Index aggregated approach	nutriRECIPE-Index disaggregated approach
228	1060.0	23.0	3.0	3.6	7.1	1.3	2.0	39.0	520.0	3	0	2	5	4	2	0	4	C	52.2 %	53.1 %
229	782.0	6.5	0.6	6.9	5.8	1.4	4.3	39.0	568.0	2	1	0	6	3	4	0	2	B	54.3 %	63.1 %
230	804.0	13.8	2.7	3.3	4.3	1.4	1.6	39.0	540.0	2	0	2	5	2	1	0	6	C	68.1 %	67.2 %
231	614.0	5.4	1.3	7.6	3.6	1.3	2.0	41.0	500.0	1	1	1	5	2	2	1	3	C	40.9 %	67.6 %
232	1189.0	28.0	4.6	3.7	3.6	1.6	2.0	39.0	640.0	3	0	4	7	2	2	0	12	D	34.1 %	34.1 %
233	1526.0	37.0	6.3	3.4	4.9	1.7	0.4	39.0	680.0	4	0	6	7	3	0	0	17	D	33.6 %	33.6 %
234	696.0	11.0	1.0	4.9	1.8	1.1	2.1	39.0	440.0	2	1	0	4	1	2	0	4	C	46.7 %	46.7 %
235	593.0	8.6	0.9	4.8	2.3	1.5	1.2	39.0	600.0	1	1	0	6	1	1	0	6	C	43.3 %	43.3 %
236	522.0	6.9	0.9	2.8	1.4	1.2	1.0	39.0	480.0	1	0	0	5	0	1	0	5	C	46.0 %	46.0 %
237	1178.0	26.8	5.2	2.7	5.4	1.6	1.4	39.0	640.0	3	0	5	7	3	1	0	14	D	42.0 %	35.6 %
238	610.0	10.7	1.2	8.4	0.9	0.9	3.2	61.0	360.0	1	1	1	3	0	3	2	1	B	47.8 %	47.8 %
239	422.0	5.0	0.4	11.9	0.4	1.8	2.1	61.0	720.0	1	2	0	7	0	2	2	6	C	37.4 %	37.4 %
240	874.0	17.0	2.0	11.0	1.0	1.3	1.8	41.0	520.0	2	2	1	5	0	1	1	8	C	29.1 %	29.1 %
241	1762.0	42.1	6.6	6.2	4.6	1.3	0.1	39.0	520.0	5	1	6	5	2	0	0	17	D	25.5 %	25.5 %
242	1693.0	40.4	6.5	4.9	5.1	1.3	0.3	39.0	520.0	5	1	6	5	3	0	0	17	D	30.7 %	29.8 %
243	894.0	18.6	1.8	9.1	0.8	1.7	3.6	61.0	680.0	2	2	1	7	0	3	2	7	C	33.6 %	37.9 %
244	988.0	18.2	2.1	3.9	3.7	1.3	0.7	39.0	512.0	2	0	2	5	2	0	0	7	C	24.2 %	26.3 %
245	689.0	5.3	0.4	3.3	3.9	1.3	4.7	39.0	532.0	2	0	0	5	2	4	0	1	B	84.6 %	65.8 %
246	724.0	4.3	0.4	8.9	4.9	1.5	6.2	41.0	600.0	2	1	0	6	3	5	1	0	B	81.9 %	69.8 %

Product	Energy (KJ/100 g or 100 ml)	Total fat (g/100 g or 100 ml)	Saturated fatty acids (g/100 g or 100 ml)	Sugars (g/100 g or 100 ml)	Proteins (g/100 g or 100 ml)	Salt (g/100 g or 100 ml)	Fiber (g/100 g or 100 ml)	Fruits, vegetables, pulses, nuts, and rapeseed, walnut and olive oils (%/100 g or 100 ml)	Sodium (mg/100 g or 100 ml)	Points for energy	Points for sugar	Points for saturated fatty acids	Point for sodium	Points for protein	Points for fiber	Points for fruits etc.	Total score	Nutri-Score	nutriRECIPE-Index aggregated approach	nutriRECIPE-Index disaggregated approach
247	743.0	14.9	1.3	7.0	2.6	1.5	3.2	41.0	588.0	2	1	1	6	1	3	1	5	C	45.2 %	45.5 %
248	696.0	5.0	0.7	5.1	4.3	1.6	2.8	39.0	640.0	2	1	0	7	2	2	0	6	C	42.2 %	46.4 %
249	859.0	16.9	6.4	2.5	8.6	2.0	0.5	39.0	800.0	2	0	6	8	5	0	0	16	D	58.2 %	64.8 %
250	884.0	18.5	6.7	3.2	7.8	2.0	0.6	39.0	800.0	2	0	6	8	4	0	0	16	D	51.9 %	55.6 %
251	801.0	9.1	0.9	5.0	4.7	1.4	3.3	39.0	560.0	2	1	0	6	2	3	0	4	C	62.7 %	61.9 %
252	646.0	6.3	0.8	6.1	4.5	1.5	2.5	39.0	600.0	1	1	0	6	2	2	0	4	C	60.1 %	81.9 %
253	958.0	20.0	2.8	4.1	8.4	1.6	0.6	39.0	640.0	2	0	2	7	5	0	0	11	D	72.6 %	72.6 %
254	883.0	16.0	1.3	6.1	9.7	1.2	2.0	39.0	480.0	2	1	1	5	5	2	0	2	B	46.3 %	46.3 %
255	1259.0	28.4	4.9	3.0	8.7	1.5	0.0	39.0	608.0	3	0	4	6	5	0	0	13	D	60.3 %	60.9 %
256	1544.0	36.8	4.9	1.8	8.8	1.5	0.1	39.0	592.0	4	0	4	6	5	0	0	14	D	60.8 %	60.8 %
257	1270.0	27.6	4.2	5.1	8.9	1.3	0.4	39.0	520.0	3	1	4	5	5	0	0	13	D	65.8 %	76.3 %
258	1279.0	25.0	2.2	8.4	10.9	1.2	0.4	39.0	480.0	3	1	2	5	5	0	0	11	D	33.7 %	47.1 %
259	491.0	5.7	0.4	4.7	1.5	1.7	0.9	39.0	680.0	1	1	0	7	0	1	0	8	C	36.0 %	39.3 %
260	988.0	19.6	1.8	5.5	9.0	1.3	2.0	39.0	500.0	2	1	1	5	5	2	0	2	B	51.9 %	38.5 %
261	968.0	13.0	2.2	2.8	5.0	1.4	2.8	41.0	560.0	2	0	2	6	3	3	1	3	C	66.4 %	72.4 %
262	769.0	11.0	1.8	2.0	3.4	3.1	2.8	39.0	1240.0	2	0	1	10	2	2	0	11	D	82.3 %	82.1 %
263	380.0	3.3	0.4	1.8	6.6	0.6	1.8	61.0	224.0	1	0	0	2	4	1	2	-4	A	93.3 %	105.3 %
264	580.0	7.8	0.7	9.2	6.0	1.5	1.8	41.0	596.0	1	2	0	6	3	1	1	4	C	75.6 %	73.7 %
265	709.0	12.4	3.8	7.2	6.3	1.6	1.7	39.0	656.0	2	1	3	7	3	1	0	12	D	63.3 %	48.0 %

Product	Energy (KJ/100 g or 100 ml)	Total fat (g/100 g or 100 ml)	Saturated fatty acids (g/100 g or 100 ml)	Sugars (g/100 g or 100 ml)	Proteins (g/100 g or 100 ml)	Salt (g/100 g or 100 ml)	Fiber (g/100 g or 100 ml)	Fruits, vegetables, pulses, nuts, and rapeseed, walnut and olive oils (%/100 g or 100 ml)	Sodium (mg/100 g or 100 ml)	Points for energy	Points for sugar	Points for saturated fatty acids	Point for sodium	Points for protein	Points for fiber	Points for fruits etc.	Total score	Nutri-Score	nutriRECIPE-Index aggregated approach	nutriRECIPE-Index disaggregated approach
266	461.0	5.0	1.0	6.4	9.3	1.7	1.7	41.0	660.0	1	1	0	7	5	1	1	2	B	93.7 %	88.0 %
267	425.0	3.2	0.6	4.8	4.1	1.2	3.1	41.0	480.0	1	1	0	5	2	3	1	1	B	99.8 %	99.5 %
268	487.0	2.7	1.1	4.2	5.7	1.2	5.4	41.0	480.0	1	0	1	5	3	5	1	-2	A	102.4 %	114.9 %
269	475.0	6.0	0.5	3.7	1.4	1.4	2.2	39.0	568.0	1	0	0	6	0	2	0	5	C	54.4 %	51.5 %
270	523.0	7.1	0.5	2.7	1.5	1.3	1.1	39.0	520.0	1	0	0	5	0	1	0	5	C	48.8 %	49.9 %
271	562.0	6.0	1.0	4.3	2.7	1.8	1.5	39.0	700.0	1	0	0	7	1	1	0	6	C	45.0 %	45.1 %
272	493.0	6.5	0.8	3.8	1.5	2.0	1.4	39.0	812.0	1	0	0	9	0	1	0	9	C	43.1 %	43.1 %
273	1126.0	17.0	2.0	3.9	5.1	2.0	3.7	41.0	800.0	3	0	1	8	3	4	1	7	C	88.5 %	74.8 %
274	1126.0	9.3	1.2	6.3	2.6	2.6	2.9	39.0	1040.0	3	1	1	10	1	3	0	12	D	83.2 %	123.1 %
275	1437.0	32.7	3.1	2.6	10.4	1.6	0.5	39.0	620.0	4	0	3	6	5	0	0	13	D	47.8 %	47.8 %
276	514.0	5.4	0.6	2.7	2.9	1.4	3.4	61.0	560.0	1	0	0	6	1	3	2	1	B	95.6 %	100.7 %
277	528.0	2.9	0.4	3.6	3.4	1.2	3.7	41.0	480.0	1	0	0	5	2	3	1	0	B	98.8 %	79.4 %
278	681.0	7.5	1.1	3.6	5.7	1.4	6.5	41.0	540.0	2	0	1	5	3	5	1	-1	A	75.7 %	78.0 %
279	1051.0	14.9	1.4	5.1	5.5	1.4	3.6	41.0	560.0	3	1	1	6	3	3	1	7	C	65.7 %	64.5 %
280	671.0	6.7	0.6	4.8	5.2	1.3	3.7	41.0	500.0	2	1	0	5	3	3	1	1	B	70.6 %	79.1 %
281	586.0	5.7	2.0	2.4	6.7	1.1	3.3	41.0	420.0	1	0	1	4	4	3	1	-2	A	83.1 %	92.6 %
282	503.0	5.5	0.8	5.6	2.1	1.5	1.1	39.0	580.0	1	1	0	6	1	1	0	6	C	39.3 %	39.3 %
283	606.0	7.4	1.3	4.2	3.0	1.5	1.0	39.0	600.0	1	0	1	6	1	1	0	6	C	45.2 %	59.4 %
284	623.0	5.5	0.7	3.3	3.5	1.0	3.8	41.0	400.0	1	0	0	4	2	4	1	-2	A	81.1 %	63.5 %

Product	Energy *(KJ/100 g or 100 ml)*	Total fat *(g/100 g or 100 ml)*	Saturated fatty acids *(g/100 g or 100 ml)*	Sugars *(g/100 g or 100 ml)*	Proteins *(g/100 g or 100 ml)*	Salt *(g/100 g or 100 ml)*	Fiber *(g/100 g or 100 ml)*	Fruits, vegetables, pulses, nuts, and rapeseed, walnut and olive oils *(%/100 g or 100 ml)*	Sodium *(mg/100 g or 100 ml)*	Points for energy	Points for sugar	Points for saturated fatty acids	Point for sodium	Points for protein	Points for fiber	Points for fruits etc.	Total score	Nutri-Score	nutriRECIPE-Index aggregated approach	nutriRECIPE-Index disaggregated approach
285	673.0	7.9	3.0	0.1	7.4	0.6	4.3	39.0	240.0	2	0	2	2	4	4	0	-2	A	82.3 %	80.1 %
286	499.0	2.7	0.3	2.7	4.0	1.1	4.3	39.0	440.0	1	0	0	4	2	4	0	-1	A	90.2 %	74.9 %
287	613.0	5.8	1.6	1.5	7.5	0.7	2.3	41.0	280.0	1	0	1	3	4	2	1	-2	A	89.2 %	93.9 %
288	654.0	8.8	0.9	1.8	6.3	0.5	5.4	41.0	212.0	1	0	0	2	3	5	1	-6	A	99.4 %	98.1 %
289	512.0	5.1	0.5	3.1	3.9	0.6	3.4	61.0	220.0	1	0	0	2	2	3	2	-4	A	85.9 %	94.2 %
290	636.1	5.9	0.7	1.6	4.1	1.1	3.8	41.0	440.0	1	0	0	4	2	4	1	-2	A	78.3 %	82.2 %
291	780.0	8.5	1.0	6.3	5.0	1.3	5.5	39.0	500.0	2	1	0	5	3	5	0	0	B	67.4 %	68.3 %
292	859.0	8.0	0.6	1.3	4.7	0.9	4.7	39.0	360.0	2	0	0	3	2	4	0	-1	A	82.4 %	53.8 %
293	863.0	11.0	0.9	2.3	8.3	1.6	4.5	41.0	640.0	2	0	0	7	5	4	1	-1	A	83.3 %	81.4 %
294	489.0	5.2	1.3	5.6	3.8	0.9	5.2	39.0	360.0	1	1	1	3	2	5	0	-1	A	70.2 %	74.9 %

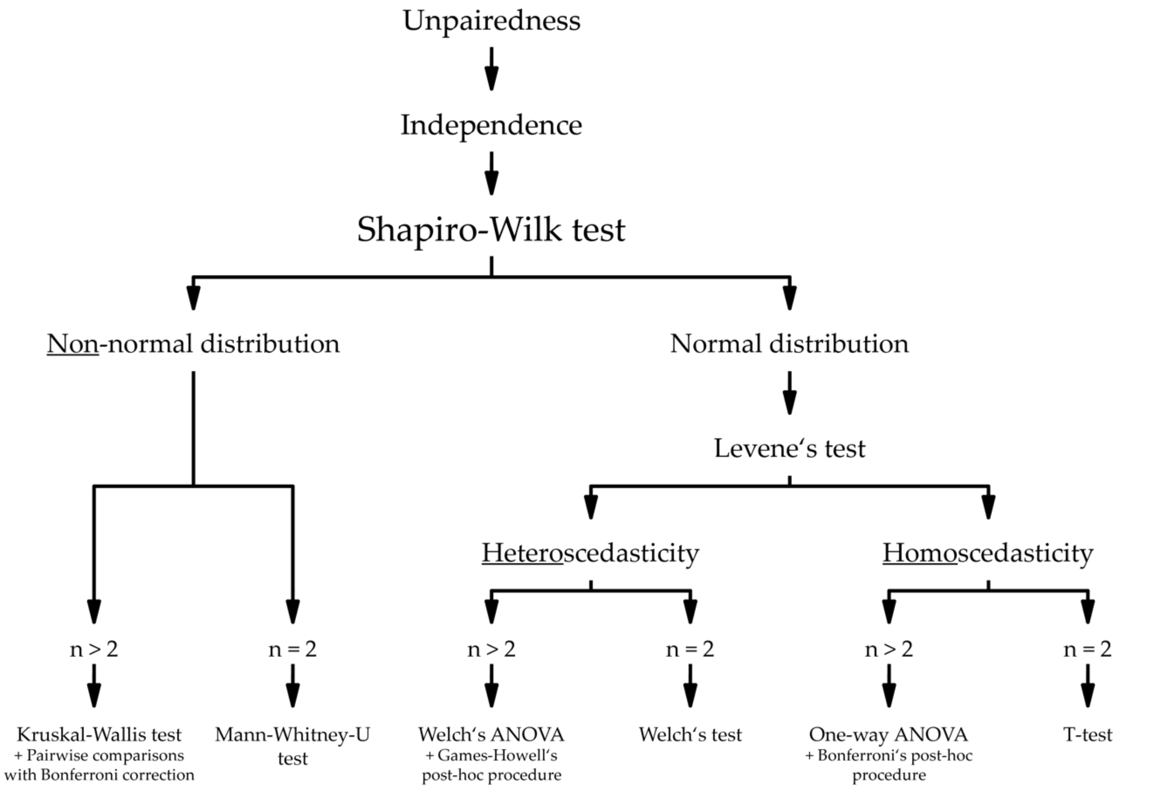

Figure S1. Decision tree for determining the appropriate test.
The tree depicts the order and selection of statistical tests for significant differences between groups. The number of groups between which a comparison is made is described by n. Modified after Field [30] and Rosenthal [31].
n: Number of groups compared

Table S4. Comparison of nutriRECIPE-Index and nutrition content per 100 g among main groups (n = 294).

				Main group															
				Leaf salads (a)				Raw food salads (b)				Starch-based salads (c)				Protein-based salads (d)			
Nutrient	ND	HO	Test	Median	p-Value to			Median	p-Value to			Median	p-Value to			Median	p-Value to		
					b	c	d		a	c	d		a	b	d		a	b	c
Energy (kJ)	N	-	Kruskal-Wallis test	447.0	1.000	0.000	0.000	443.0	1.000	0.000	0.000	713.0	0.000	0.000	0.314	961.0	0.000	0.000	0.314
Fat (g)	N	-	Kruskal-Wallis test	6.7	1.000	0.003	0.000	5.0	1.000	0.029	0.000	8.1	0.003	0.029	0.001	19.8	0.000	0.000	0.001
Saturated fatty acids (g)	N	-	Kruskal-Wallis test	1.2	0.012	1.000	0.000	0.7	0.012	0.258	0.000	0.9	1.000	0.258	0.000	3.0	0.000	0.000	0.000
Carbohydrates (g)	N	-	Kruskal-Wallis test	6.5	0.077	0.000	1.000	10.0	0.077	0.000	0.012	17.0	0.000	0.000	0.000	6.2	1.000	0.012	0.000
Sugar (g)	N	-	Kruskal-Wallis test	3.6	0.000	0.543	0.677	8.4	0.000	0.000	0.000	4.1	0.543	0.000	1.000	4.2	0.677	0.000	1.000
Protein (g)	N	-	Kruskal-Wallis test	4.2	0.000	1.000	0.000	1.0	0.000	0.000	0.000	3.4	1.000	0.000	0.000	7.1	0.000	0.000	0.000
Salt (g)	N	-	Kruskal-Wallis test	0.7	0.000	0.000	0.000	1.1	0.000	0.006	0.002	1.4	0.000	0.006	1.000	1.5	0.000	0.002	1.000
Price (€)	N	-	Kruskal-Wallis test	0.74	0.000	0.025	1.000	0.57	0.000	0.116	0.025	0.70	0.025	0.116	1.000	0.70	1.000	0.025	1.000

HO: Homoscedasticity; N: No; ND: Normal distribution

Table S5. Comparison of nutriRECIPE-Index and nutrition content per 100 g according to diets within leaf salads (n = 107).

				Criteria															
				Meat (a)				Fish (b)				Vegetarian (c)				Vegan (d)			
Nutrient	ND	HO	Test	Median	p-Value to			Median	p-Value to			Median	p-Value to			Median	p-Value to		
					b	c	d		a	c	d		a	b	d		a	b	c
Energy (kJ)	N	-	Kruskal-Wallis test	509.0	0.166	0.093	0.000	430.0	0.166	0.792	0.042	436.5	0.093	0.792	0.004	195.5	0.000	0.042	0.004
Fat (g)	N	-	Kruskal-Wallis test	7.3	0.116	0.602	0.000	5.8	0.116	0.233	0.147	7.4	0.602	0.233	0.002	1.7	0.000	0.147	0.002
Saturated fatty acids (g)	N	-	Kruskal-Wallis test	1.8	0.005	0.601	0.000	0.6	0.005	0.015	0.200	1.4	0.601	0.015	0.000	0.2	0.000	0.200	0.000
Carbohydrates (g)	N	-	Kruskal-Wallis test	8.6	*	*	*	10.0	*	*	*	6.2	*	*	*	5.2	*	*	*
Sugar (g)	N	-	Kruskal-Wallis test	3.5	0.813	0.017	0.135	3.3	0.813	0.188	0.184	4.2	0.017	0.188	0.002	3.0	0.135	0.184	0.002
Protein (g)	N	-	Kruskal-Wallis test	4.7	0.191	0.000	0.000	4.6	0.191	0.158	0.001	3.3	0.000	0.158	0.005	1.6	0.000	0.001	0.005
Salt (g)	Y	Y	One-way ANOVA	0.8	1.000	0.691	0.002	0.7	1.000	1.000	0.154	0.7	0.691	1.000	0.099	0.5	0.002	0.154	0.099
Fiber (g)	N	-	Kruskal-Wallis test	1.5	0.429	0.282	0.002	1.7	0.429	0.939	0.096	1.6	0.282	0.939	0.027	2.0	0.002	0.096	0.027
Vitamin D (µg)	N	-	Kruskal-Wallis test	0.1	0.001	0.198	0.000	0.5	0.001	0.000	0.000	0.0	0.198	0.000	0.000	0.0	0.000	0.000	0.000
Vitamin E (µg)	N	-	Kruskal-Wallis test	1399.4	0.295	0.406	0.008	1237.4	0.295	0.119	0.261	1620.5	0.406	0.119	0.002	908.2	0.008	0.261	0.002
Thiamine (µg)	N	-	Kruskal-Wallis test	72.2	0.068	0.000	0.666	58.3	0.068	0.343	0.232	48.5	0.000	0.343	0.010	64.7	0.666	0.232	0.010
Riboflavin (µg)	N	-	Kruskal-Wallis test	66.2	0.030	0.446	0.000	51.0	0.030	0.104	0.102	63.4	0.446	0.104	0.000	44.7	0.000	0.102	0.000
Vitamin B6 (µg)	N	-	Kruskal-Wallis test	97.1	0.605	0.000	0.001	109.7	0.605	0.000	0.004	69.9	0.000	0.000	0.118	110.2	0.001	0.004	0.118
Folate (µg)	N	-	Kruskal-Wallis test	28.1	0.843	0.122	0.001	32.8	0.843	0.421	0.015	34.6	0.122	0.421	0.025	44.6	0.001	0.015	0.025
Vitamin B12 (µg)	N	-	Kruskal-Wallis test	0.2	0.018	0.067	0.000	0.4	0.018	0.000	0.000	0.1	0.067	0.000	0.000	0.0	0.000	0.000	0.000
Vitamin C (µg)	N	-	Kruskal-Wallis test	8801.1	0.433	0.250	0.001	11608.6	0.433	0.983	0.055	11296.7	0.250	0.983	0.013	15985.0	0.001	0.055	0.013
Calcium (mg)	N	-	Kruskal-Wallis test	70.6	0.036	0.249	0.002	37.3	0.036	0.005	0.526	66.2	0.249	0.005	0.000	35.0	0.002	0.526	0.000
Magnesium (mg)	N	-	Kruskal-Wallis test	16.3	0.548	0.030	0.035	17.1	0.548	0.047	0.036	13.8	0.030	0.047	0.607	13.4	0.035	0.036	0.607
Iron (µg)	N	-	Kruskal-Wallis test	944.2	*	*	*	1061.7	*	*	*	833.8	*	*	*	819.6	*	*	*
Zinc (µg)	N	-	Kruskal-Wallis test	714.8	0.001	0.015	0.000	341.1	0.001	0.085	0.394	510.4	0.015	0.085	0.003	340.3	0.000	0.394	0.003
Iodide (µg)	N	-	Kruskal-Wallis test	4.1	0.074	0.242	0.006	7.1	0.074	0.013	0.000	3.3	0.242	0.013	0.066	2.6	0.006	0.000	0.066
nutriRECIPE-Index (%)	N	-	Kruskal-Wallis test	71.4	0.085	0.759	0.001	82.9	0.085	0.061	0.288	70.4	0.759	0.061	0.001	90.9	0.001	0.288	0.001
MUFA (g)	N	-	Kruskal-Wallis test	3.0	*	*	*	2.5	*	*	*	2.8	*	*	*	0.3	*	*	*
PUFA (g)	N	-	Kruskal-Wallis test	1.4	*	*	*	1.3	*	*	*	1.4	*	*	*	0.3	*	*	*

HO: Homoscedasticity; MUFA: Monounsaturated fatty acids; N: No; ND: Normal distribution; PUFA: Polyunsaturated fatty acids; Y: Yes

*: Multiple comparisons were not applied because the test showed no significant differences between samples

Table S6. Comparison of nutriRECIPE-Index and nutrition content per 100 g according to presence of a dressing within leaf salads (n = 107).

				Criteria		
				Dressing (+)	Dressing (-)	p-Value
Nutrient	ND	HO	Test	Median	Median	
Energy (kJ)	N	-	Mann-Whitney-U test	471.5	231.0	0.000
Fat (g)	N	-	Mann-Whitney-U test	7.2	1.0	0.000
Saturated fatty acids (g)	N	-	Mann-Whitney-U test	1.3	0.3	0.000
Carbohydrates (g)	N	-	Mann-Whitney-U test	7.8	4.4	0.000
Sugar (g)	N	-	Mann-Whitney-U test	3.9	1.9	0.000
Protein (g)	N	-	Mann-Whitney-U test	4.2	4.7	0.498
Salt (g)	Y	Y	T-test	0.8	0.2	0.000
Fiber (g)	N	-	Mann-Whitney-U test	1.6	2.0	0.002
Vitamin D (µg)	N	-	Mann-Whitney-U test	0.1	0.0	0.005
Vitamin E (µg)	N	-	Mann-Whitney-U test	1434.4	921.3	0.000
Thiamine (µg)	N	-	Mann-Whitney-U test	57.1	70.9	0.001
Riboflavin (µg)	N	-	Mann-Whitney-U test	61.1	69.4	0.809
Vitamin B6 (µg)	N	-	Mann-Whitney-U test	88.8	114.1	0.187
Folate (µg)	N	-	Mann-Whitney-U test	29.9	68.4	0.000
Vitamin B12 (µg)	N	-	Mann-Whitney-U test	0.2	0.1	0.091
Vitamin C (µg)	N	-	Mann-Whitney-U test	9332.3	12946.2	0.011
Calcium (mg)	N	-	Mann-Whitney-U test	57.2	37.2	0.102
Magnesium (mg)	N	-	Mann-Whitney-U test	16.4	16.1	0.251
Iron (µg)	N	-	Mann-Whitney-U test	888.0	1179.8	0.033
Zinc (µg)	N	-	Mann-Whitney-U test	529.9	471.1	0.524
Iodide (µg)	N	-	Mann-Whitney-U test	3.8	4.4	0.311
nutriRECIPE-Index (%)	N	-	Mann-Whitney-U test	71.1	124.2	0.000
MUFA (g)	N	-	Mann-Whitney-U test	3.0	0.2	0.615
PUFA (g)	N	-	Mann-Whitney-U test	1.4	0.3	0.501

HO: Homoscedasticity; MUFA: Monounsaturated fatty acids; N: No; ND: Normal distribution; PUFA: Polyunsaturated fatty acids; Y: Yes

Table S7. Comparison of nutriRECIPE-Index and nutrition content per 100 g according to dressing base within leaf salads (n = 107).

				Criteria															
				Yoghurt dressing (a)				Mayonnaise (b)				Mustard sauce (c)				Vinaigrette (d)			
Nutrient	ND	HO	Test	Median	p-Value to			Median	p-Value to			Median	p-Value to			Median	p-Value to		
					b	c	d		a	c	d		a	b	d		a	b	c
Energy (kJ)	N	-	Kruskal-Wallis test	460.5	0.006	0.004	0.001	598.0	0.006	0.924	0.000	595.0	0.004	0.924	0.000	377.0	0.001	0.000	0.000
Fat (g)	N	-	Kruskal-Wallis test	8.2	0.099	0.899	0.000	10.1	0.099	0.152	0.000	8.5	0.899	0.152	0.000	3.0	0.000	0.000	0.000
Saturated fatty acids (g)	N	-	Kruskal-Wallis test	1.3	0.045	0.874	0.007	2.5	0.045	0.137	0.000	1.7	0.874	0.137	0.028	1.1	0.007	0.000	0.028
Carbohydrates (g)	N	-	Kruskal-Wallis test	5.9	0.022	0.000	0.001	10.0	0.022	0.032	0.650	14.9	0.000	0.032	0.060	10.8	0.001	0.650	0.060
Sugar (g)	N	-	Kruskal-Wallis test	3.5	0.517	0.000	0.004	3.4	0.517	0.000	0.005	7.3	0.000	0.000	0.032	5.0	0.004	0.005	0.032
Protein (g)	N	-	Kruskal-Wallis test	4.0	0.009	0.032	0.090	4.8	0.009	0.704	0.000	4.5	0.032	0.704	0.002	3.3	0.090	0.000	0.002
Salt (g)	Y	Y	One-way ANOVA	0.8	1.000	1.000	1.000	0.7	1.000	1.000	1.000	0.9	1.000	1.000	1.000	0.8	1.000	1.000	1.000
Fiber (g)	N	-	Kruskal-Wallis test	1.5	*	*	*	1.5	*	*	*	1.6	*	*	*	1.7	*	*	*
Vitamin D (µg)	N	-	Kruskal-Wallis test	0.1	0.217	0.751	0.005	0.1	0.217	0.461	0.001	0.1	0.751	0.461	0.015	0.0	0.005	0.001	0.015
Vitamin E (µg)	N	-	Kruskal-Wallis test	1687.3	0.198	0.320	0.000	1957.3	0.198	0.066	0.000	1400.5	0.320	0.066	0.010	783.8	0.000	0.000	0.010
Thiamine (µg)	N	-	Kruskal-Wallis test	59.8	0.557	0.279	0.009	60.9	0.557	0.179	0.013	43.0	0.279	0.179	0.303	43.8	0.009	0.013	0.303
Riboflavin (µg)	N	-	Kruskal-Wallis test	65.3	0.929	0.703	0.000	66.2	0.929	0.814	0.000	64.8	0.703	0.814	0.001	42.8	0.000	0.000	0.001
Vitamin B6 (µg)	N	-	Kruskal-Wallis test	93.4	0.020	0.015	0.727	104.9	0.020	0.927	0.021	83.8	0.015	0.927	0.016	79.6	0.727	0.021	0.016
Folate (µg)	N	-	Kruskal-Wallis test	31.0	*	*	*	29.4	*	*	*	26.5	*	*	*	29.7	*	*	*
Vitamin B12 (µg)	N	-	Kruskal-Wallis test	0.2	*	*	*	0.2	*	*	*	0.2	*	*	*	0.1	*	*	*
Vitamin C (µg)	N	-	Kruskal-Wallis test	9593.2	0.062	0.024	0.328	6482.5	0.062	0.750	0.017	7915.4	0.024	0.750	0.006	11721.4	0.328	0.017	0.006
Calcium (mg)	N	-	Kruskal-Wallis test	61.5	*	*	*	82.7	*	*	*	59.9	*	*	*	39.2	*	*	*
Magnesium (mg)	N	-	Kruskal-Wallis test	16.0	0.030	0.001	0.225	19.0	0.030	0.374	0.005	20.4	0.001	0.374	0.000	13.9	0.225	0.005	0.000
Iron (µg)	N	-	Kruskal-Wallis test	825.3	0.010	0.261	0.724	1155.3	0.010	0.245	0.012	994.4	0.261	0.245	0.209	807.8	0.724	0.012	0.209
Zinc (µg)	N	-	Kruskal-Wallis test	487.5	0.015	0.643	0.020	860.7	0.015	0.113	0.000	636.1	0.643	0.113	0.029	341.1	0.020	0.000	0.029
Iodide (µg)	N	-	Kruskal-Wallis test	2.8	0.037	0.001	0.013	4.7	0.037	0.274	0.951	12.0	0.001	0.274	0.259	6.1	0.013	0.951	0.259
nutriRECIPE-Index (%)	N	-	Kruskal-Wallis test	73.5	0.375	0.001	0.509	69.6	0.375	0.040	0.200	53.6	0.001	0.040	0.000	76.1	0.509	0.200	0.000
MUFA (g)	N	-	Kruskal-Wallis test	3.2	*	*	*	3.4	*	*	*	3.7	*	*	*	1.1	*	*	*
PUFA (g)	N	-	Kruskal-Wallis test	1.5	*	*	*	1.8	*	*	*	1.6	*	*	*	0.7	*	*	*

HO: Homoscedasticity; MUFA: Monounsaturated fatty acids; N: No; ND: Normal distribution; PUFA: Polyunsaturated fatty acids; Y: Yes

*: Multiple comparisons were not applied because the test showed no significant differences between samples

Table S8. Comparison of nutriRECIPE-Index and nutrition content per 100 g according to presence of a side dish within leaf salads (n = 107).

				Criteria		
				Side dish (+)	Side dish (-)	p-Value
Nutrient	ND	HO	Test	Median	Median	
Energy (kJ)	N	-	Mann-Whitney-U test	584.0	417.0	0.000
Fat (g)	N	-	Mann-Whitney-U test	7.1	6.5	0.102
Saturated fatty acids (g)	N	-	Mann-Whitney-U test	1.6	1.0	0.021
Carbohydrates (g)	N	-	Mann-Whitney-U test	14.0	5.6	0.000
Sugar (g)	N	-	Mann-Whitney-U test	3.8	3.5	0.404
Protein (g)	N	-	Mann-Whitney-U test	4.6	3.7	0.001
Salt (g)	Y	N	Welch's test	0.7	0.7	0.772
Fiber (g)	N	-	Mann-Whitney-U test	1.6	1.7	0.319
Vitamin D (µg)	N	-	Mann-Whitney-U test	0.1	0.0	0.438
Vitamin E (µg)	N	-	Mann-Whitney-U test	1495.9	1314.7	0.354
Thiamine (µg)	N	-	Mann-Whitney-U test	60.0	60.9	0.446
Riboflavin (µg)	N	-	Mann-Whitney-U test	66.2	59.0	0.951
Vitamin B6 (µg)	N	-	Mann-Whitney-U test	85.8	92.9	0.089
Folate (µg)	N	-	Mann-Whitney-U test	22.4	34.8	0.000
Vitamin B12 (µg)	N	-	Mann-Whitney-U test	0.2	0.1	0.146
Vitamin C (µg)	N	-	Mann-Whitney-U test	6482.5	10525.7	0.001
Calcium (mg)	N	-	Mann-Whitney-U test	59.3	46.2	0.546
Magnesium (mg)	N	-	Mann-Whitney-U test	19.5	14.9	0.000
Iron (µg)	N	-	Mann-Whitney-U test	939.4	904.4	0.789
Zinc (µg)	N	-	Mann-Whitney-U test	642.6	459.8	0.018
Iodide (µg)	N	-	Mann-Whitney-U test	6.0	3.5	0.006
nutriRECIPE-Index (%)	N	-	Mann-Whitney-U test	62.3	79.7	0.000
MUFA (g)	N	-	Mann-Whitney-U test	2.8	2.6	0.471
PUFA (g)	N	-	Mann-Whitney-U test	1.8	1.2	0.318

HO: Homoscedasticity; MUFA: Monounsaturated fatty acids; N: No; ND: Normal distribution; PUFA: Polyunsaturated fatty acids; Y: Yes

Table S9. Comparison of nutriRECIPE-Index and nutrition content per 100 g according to the type of side dish within leaf salads (n = 107).

				Criteria		
				Side dish (pasta)	Side dish (others)	p-Value
Nutrient	ND	HO	Test	Median	Median	
Energy (kJ)	N	-	Mann-Whitney-U test	544.0	590.0	0.746
Fat (g)	N	-	Mann-Whitney-U test	5.9	8.7	0.745
Saturated fatty acids (g)	N	-	Mann-Whitney-U test	1.6	1.4	0.685
Carbohydrates (g)	N	-	Mann-Whitney-U test	14.0	12.8	0.669
Sugar (g)	N	-	Mann-Whitney-U test	3.3	5.5	0.040
Protein (g)	N	-	Mann-Whitney-U test	4.6	4.5	0.230
Salt (g)	Y	Y	T-test	0.7	0.8	0.867
Fiber (g)	N	-	Mann-Whitney-U test	1.5	1.8	0.026
Vitamin D (µg)	N	-	Mann-Whitney-U test	0.1	0.1	0.807
Vitamin E (µg)	N	-	Mann-Whitney-U test	1233.4	1581.5	0.209
Thiamine (µg)	N	-	Mann-Whitney-U test	38.1	81.6	0.003
Riboflavin (µg)	N	-	Mann-Whitney-U test	61.4	68.7	0.168
Vitamin B6 (µg)	N	-	Mann-Whitney-U test	83.7	95.3	0.351
Folate (µg)	N	-	Mann-Whitney-U test	19.2	28.7	0.003
Vitamin B12 (µg)	N	-	Mann-Whitney-U test	0.2	0.2	0.655
Vitamin C (µg)	N	-	Mann-Whitney-U test	6097.1	10274.9	0.011
Calcium (mg)	N	-	Mann-Whitney-U test	65.5	56.6	0.839
Magnesium (mg)	N	-	Mann-Whitney-U test	19.0	27.2	0.011
Iron (µg)	N	-	Mann-Whitney-U test	807.8	1072.2	0.026
Zinc (µg)	N	-	Mann-Whitney-U test	667.7	562.6	0.516
Iodide (µg)	N	-	Mann-Whitney-U test	6.0	5.8	0.240
nutriRECIPE-Index (%)	N	-	Mann-Whitney-U test	57.9	63.4	0.292
MUFA (g)	N	-	Mann-Whitney-U test	2.6	3.4	0.598
PUFA (g)	N	-	Mann-Whitney-U test	1.4	2.0	0.808

HO: Homoscedasticity; MUFA: Monounsaturated fatty acids; N: No; ND: Normal distribution; PUFA: Polyunsaturated fatty acids; Y: Yes

Table S10. Comparison of nutriRECIPE-Index and nutrition content per 100 g according to the brand within leaf salads (n = 107).

				Criteria		
				Private label	Branded label	p-Value
Nutrient	ND	HO	Test	Median	Median	
Energy (kJ)	N	-	Mann-Whitney-U test	470.0	427.5	0.045
Fat (g)	N	-	Mann-Whitney-U test	7.2	5.9	0.126
Saturated fatty acids (g)	N	-	Mann-Whitney-U test	1.4	1.0	0.140
Carbohydrates (g)	N	-	Mann-Whitney-U test	7.4	5.4	0.030
Sugar (g)	N	-	Mann-Whitney-U test	4.1	3.2	0.002
Protein (g)	N	-	Mann-Whitney-U test	4.2	4.3	0.784
Salt (g)	Y	Y	T-test	0.8	0.6	0.000
Fiber (g)	N	-	Mann-Whitney-U test	1.6	1.7	0.801
Vitamin D (µg)	N	-	Mann-Whitney-U test	0.1	0.0	0.780
Vitamin E (µg)	N	-	Mann-Whitney-U test	1391.1	1227.2	0.564
Thiamine (µg)	N	-	Mann-Whitney-U test	61.3	57.0	0.653
Riboflavin (µg)	N	-	Mann-Whitney-U test	60.7	64.3	0.551
Vitamin B6 (µg)	N	-	Mann-Whitney-U test	89.9	98.3	0.700
Folate (µg)	N	-	Mann-Whitney-U test	29.6	39.2	0.000
Vitamin B12 (µg)	N	-	Mann-Whitney-U test	0.2	0.1	0.229
Vitamin C (µg)	N	-	Mann-Whitney-U test	9304.1	10525.7	0.229
Calcium (mg)	N	-	Mann-Whitney-U test	58.3	46.2	0.416
Magnesium (mg)	N	-	Mann-Whitney-U test	16.5	15.7	0.183
Iron (µg)	N	-	Mann-Whitney-U test	914.8	885.4	0.657
Zinc (µg)	N	-	Mann-Whitney-U test	548.5	483.2	0.212
Iodide (µg)	N	-	Mann-Whitney-U test	4.2	3.6	0.334
nutriRECIPE-Index (%)	N	-	Mann-Whitney-U test	73.4	76.7	0.056
MUFA (g)	N	-	Mann-Whitney-U test	3.0	2.4	0.033
PUFA (g)	N	-	Mann-Whitney-U test	1.4	1.1	0.090

HO: Homoscedasticity; MUFA: Monounsaturated fatty acids; N: No; ND: Normal distribution; PUFA: Polyunsaturated fatty acids; Y: Yes

Table S11. Comparison of nutriRECIPE-Index and nutrition content per 100 g according to the price range within leaf salads (n = 107).

				Criteria								
				Low-priced (a) [x ≤ 0.65 €/100 g]			Medium-priced (b) [0.65 < x < 1.00 €/100 g]			High-priced (c) [x ≥ 1.00 €/100 g]		
Nutrient	ND	HO	Test	Median	p-Value to		Median	p-Value to		Median	p-Value to	
					b	c		a	c		a	b
Energy (kJ)	N	-	Kruskal-Wallis test	429.0	*	*	481.0	*	*	383.0	*	*
Fat (g)	N	-	Kruskal-Wallis test	7.1	*	*	6.7	*	*	5.6	*	*
Saturated fatty acids (g)	N	-	Kruskal-Wallis test	1.3	*	*	1.2	*	*	1.0	*	*
Carbohydrates (g)	N	-	Kruskal-Wallis test	6.5	0.032	0.045	11.2	0.032	0.000	4.9	0.045	0.000
Sugar (g)	N	-	Kruskal-Wallis test	4.2	0.149	0.000	3.8	0.149	0.002	3.0	0.000	0.002
Protein (g)	N	-	Kruskal-Wallis test	3.7	*	*	4.4	*	*	4.7	*	*
Salt (g)	Y	Y	One-way ANOVA	0.8	0.111	0.000	0.7	0.111	0.013	0.5	0.000	0.013
Fiber (g)	N	-	Kruskal-Wallis test	1.7	0.053	0.579	1.5	0.053	0.018	1.9	0.579	0.018
Vitamin D (µg)	N	-	Kruskal-Wallis test	0.1	*	*	0.1	*	*	0.0	*	*
Vitamin E (µg)	N	-	Kruskal-Wallis test	1398.3	*	*	1369.6	*	*	1227.2	*	*
Thiamine (µg)	N	-	Kruskal-Wallis test	58.7	0.327	0.080	54.6	0.327	0.006	67.3	0.080	0.006
Riboflavin (µg)	N	-	Kruskal-Wallis test	59.0	*	*	61.4	*	*	66.3	*	*
Vitamin B6 (µg)	N	-	Kruskal-Wallis test	89.9	*	*	81.0	*	*	110.6	*	*
Folate (µg)	N	-	Kruskal-Wallis test	29.7	0.956	0.001	30.4	0.956	0.001	44.2	0.001	0.001
Vitamin B12 (µg)	N	-	Kruskal-Wallis test	0.2	*	*	0.2	*	*	0.1	*	*
Vitamin C (µg)	N	-	Kruskal-Wallis test	10096.5	0.014	0.786	8071.7	0.014	0.012	10672.5	0.786	0.012
Calcium (mg)	N	-	Kruskal-Wallis test	94.4	*	*	52.9	*	*	44.8	*	*
Magnesium (mg)	N	-	Kruskal-Wallis test	16.2	*	*	16.9	*	*	16.2	*	*
Iron (µg)	N	-	Kruskal-Wallis test	899.5	*	*	887.0	*	*	910.6	*	*
Zinc (µg)	N	-	Kruskal-Wallis test	661.6	*	*	529.9	*	*	476.5	*	*
Iodide (µg)	N	-	Kruskal-Wallis test	3.7	*	*	4.4	*	*	3.9	*	*
nutriRECIPE-Index (%)	N	-	Kruskal-Wallis test	75.9	0.083	0.008	65.9	0.083	0.000	91.1	0.008	0.000
MUFA (g)	N	-	Kruskal-Wallis test	2.8	0.264	0.271	2.7	0.264	0.002	2.2	0.271	0.002

HO: Homoscedasticity; MUFA: Monounsaturated fatty acids; N: No; ND: Normal distribution; PUFA: Polyunsaturated fatty acids; Y: Yes

*: Multiple comparisons were not applied because the test showed no significant differences between samples

Table S12. Comparison of nutriRECIPE-Index and nutrition content per 100 g according to the salad base within raw food salads (n = 53).

				Criteria		
				Cabbage	Other raw vegetables	p-Value
Nutrient	ND	HO	Test	Median	Median	
Energy (kJ)	N	-	Mann-Whitney-U test	448.0	425.0	0.098
Fat (g)	N	-	Mann-Whitney-U test	5.3	3.6	0.057
Saturated fatty acids (g)	N	-	Mann-Whitney-U test	0.7	0.7	0.971
Carbohydrates (g)	Y	Y	T-test	12.0	9.8	0.463
Sugar (g)	Y	Y	T-test	10.8	8.1	0.040
Protein (g)	N	-	Mann-Whitney-U test	1.0	1.0	0.808
Salt (g)	Y	Y	T-test	1.3	0.9	0.079
Fiber (g)	N	-	Mann-Whitney-U test	2.3	2.2	0.268
Vitamin D (µg)	N	-	Mann-Whitney-U test	0.0	0.0	0.447
Vitamin E (µg)	N	-	Mann-Whitney-U test	2347.9	1028.8	0.000
Thiamine (µg)	N	-	Mann-Whitney-U test	41.0	44.4	0.475
Riboflavin (µg)	N	-	Mann-Whitney-U test	38.8	37.2	0.748
Vitamin B6 (µg)	N	-	Mann-Whitney-U test	153.9	109.4	0.000
Folate (µg)	N	-	Mann-Whitney-U test	20.6	22.3	0.748
Vitamin B12 (µg)	N	-	Mann-Whitney-U test	0.0	0.0	0.929
Vitamin C (µg)	N	-	Mann-Whitney-U test	35669.8	7396.2	0.000
Calcium (mg)	N	-	Mann-Whitney-U test	36.5	27.2	0.011
Magnesium (mg)	N	-	Mann-Whitney-U test	13.8	12.9	0.816
Iron (µg)	N	-	Mann-Whitney-U test	486.0	463.8	0.929
Zinc (µg)	N	-	Mann-Whitney-U test	201.5	252.1	0.083
Iodide (µg)	N	-	Mann-Whitney-U test	2.6	4.3	0.276
nutriRECIPE-Index (%)	N	-	Mann-Whitney-U test	47.3	48.8	0.929
MUFA (g)	N	-	Mann-Whitney-U test	2.6	2.0	0.011
PUFA (g)	N	-	Mann-Whitney-U test	1.5	1.1	0.003

HO: Homoscedasticity; MUFA: Monounsaturated fatty acids; N: No; ND: Normal distribution; PUFA: Polyunsaturated fatty acids; Y: Yes

Table S13. Comparison of nutriRECIPE-Index and nutrition content per 100 g according to the dressing base within raw food salads (n = 53).

				Criteria								
				Milk dressing (a)			Mayonnaise (b)			Vinaigrette (c)		
Nutrient	ND	HO	Test	Median	p-Value to		Median	p-Value to		Median	p-Value to	
					b	c		a	c		a	b
Energy (kJ)	N	-	Kruskal-Wallis test	874.0	0.702	0.000	711.0	0.702	0.000	377.5	0.000	0.000
Fat (g)	N	-	Kruskal-Wallis test	17.0	0.753	0.000	13.9	0.753	0.000	3.3	0.000	0.000
Saturated fatty acids (g)	N	-	Kruskal-Wallis test	1.8	0.390	0.000	1.3	0.390	0.000	0.4	0.000	0.000
Carbohydrates (g)	Y	N	Welch's ANOVA	10.3	0.135	0.527	8.2	0.135	0.021	12.1	0.527	0.021
Sugar (g)	Y	N	Welch's ANOVA	9.0	0.042	0.401	7.0	0.042	0.004	10.8	0.401	0.004
Protein (g)	N	-	Kruskal-Wallis test	1.0	*	*	1.1	*	*	1.0	*	*
Salt (g)	Y	Y	One-way ANOVA	1.3	0.713	1.000	1.0	0.713	0.614	1.2	1.000	0.614
Fiber (g)	N	-	Kruskal-Wallis test	1.8	*	*	2.8	*	*	2.2	*	*
Vitamin D (µg)	N	-	Kruskal-Wallis test	0.1	0.881	0.000	0.1	0.881	0.000	0.0	0.000	0.000
Vitamin E (µg)	N	-	Kruskal-Wallis test	3658.5	0.537	0.000	2673.6	0.537	0.001	1387.5	0.000	0.001
Thiamine (µg)	N	-	Kruskal-Wallis test	41.0	*	*	46.8	*	*	34.3	*	*
Riboflavin (µg)	N	-	Kruskal-Wallis test	46.7	0.728	0.026	45.6	0.728	0.060	34.9	0.026	0.060
Vitamin B6 (µg)	N	-	Kruskal-Wallis test	120.6	*	*	145.8	*	*	127.2	*	*
Folate (µg)	N	-	Kruskal-Wallis test	19.2	*	*	22.4	*	*	20.2	*	*
Vitamin B12 (µg)	N	-	Kruskal-Wallis test	0.1	0.523	0.000	0.1	0.523	0.000	0.0	0.000	0.000
Vitamin C (µg)	N	-	Kruskal-Wallis test	19254.5	*	*	24471.2	*	*	21970.9	*	*
Calcium (mg)	N	-	Kruskal-Wallis test	42.3	0.653	0.004	40.8	0.653	0.014	30.8	0.004	0.014
Magnesium (mg)	N	-	Kruskal-Wallis test	15.2	*	*	15.7	*	*	12.4	*	*
Iron (µg)	N	-	Kruskal-Wallis test	768.6	0.873	0.004	762.6	0.873	0.005	407.1	0.004	0.005
Zinc (µg)	N	-	Kruskal-Wallis test	330.0	0.865	0.017	328.8	0.865	0.007	193.2	0.017	0.007
Iodide (µg)	N	-	Kruskal-Wallis test	2.6	*	*	2.6	*	*	7.8	*	*
nutriRECIPE-Index (%)	N	-	Kruskal-Wallis test	30.5	0.122	0.005	45.2	0.122	0.328	55.3	0.005	0.328
MUFA (g)	N	-	Kruskal-Wallis test	6.0	*	*	5.0	*	*	1.4	*	*
PUFA (g)	N	-	Kruskal-Wallis test	3.4	*	*	2.5	*	*	0.9	*	*

HO: Homoscedasticity; MUFA: Monounsaturated fatty acids; N: No; ND: Normal distribution; PUFA: Polyunsaturated fatty acids; Y: Yes

*: Multiple comparisons were not applied because the test showed no significant differences between samples

Table S14. Comparison of nutriRECIPE-Index and nutrition content per 100 g according to the diet within raw food salads (n = 53).

				Criteria		
				Vegan (+)	Vegan (-)	p-Value
Nutrient	ND	HO	Test	Median	Median	
Energy (kJ)	N	-	Mann-Whitney-U test	383.0	678.0	0.000
Fat (g)	N	-	Mann-Whitney-U test	3.4	13.4	0.000
Saturated fatty acids (g)	N	-	Mann-Whitney-U test	0.4	1.3	0.000
Carbohydrates (g)	Y	Y	T-test	12.8	8.2	0.000
Sugar (g)	Y	Y	T-test	10.5	7.4	0.005
Protein (g)	N	-	Mann-Whitney-U test	1.0	1.0	0.367
Salt (g)	Y	Y	T-test	1.3	1.0	0.117
Fiber (g)	N	-	Mann-Whitney-U test	2.2	2.4	0.886
Vitamin D (µg)	N	-	Mann-Whitney-U test	0.0	0.1	0.000
Vitamin E (µg)	N	-	Mann-Whitney-U test	1219.0	2625.3	0.000
Thiamine (µg)	N	-	Mann-Whitney-U test	36.1	44.5	0.475
Riboflavin (µg)	N	-	Mann-Whitney-U test	35.8	46.2	0.006
Vitamin B6 (µg)	N	-	Mann-Whitney-U test	133.6	120.6	0.555
Folate (µg)	N	-	Mann-Whitney-U test	20.1	22.3	0.381
Vitamin B12 (µg)	N	-	Mann-Whitney-U test	0.0	0.1	0.000
Vitamin C (µg)	N	-	Mann-Whitney-U test	21296.5	20847.7	0.567
Calcium (mg)	N	-	Mann-Whitney-U test	32.4	41.6	0.007
Magnesium (mg)	N	-	Mann-Whitney-U test	12.7	14.5	0.497
Iron (µg)	N	-	Mann-Whitney-U test	417.7	719.5	0.056
Zinc (µg)	N	-	Mann-Whitney-U test	188.9	314.1	0.003
Iodide (µg)	N	-	Mann-Whitney-U test	7.1	2.6	0.033
nutriRECIPE-Index (%)	N	-	Mann-Whitney-U test	57.1	43.4	0.061
MUFA (g)	N	-	Mann-Whitney-U test	1.3	5.0	0.313
PUFA (g)	N	-	Mann-Whitney-U test	0.9	2.4	0.127

HO: Homoscedasticity; MUFA: Monounsaturated fatty acids; N: No; ND: Normal distribution; PUFA: Polyunsaturated fatty acids; Y: Yes

Table S15. Comparison of nutriRECIPE-Index and nutrition content per 100 g according to the brand within raw food salads (n = 53).

				Criteria		
				Private label	Branded label	p-Value
Nutrient	ND	HO	Test	Median	Median	
Energy (kJ)	N	-	Mann-Whitney-U test	463.0	442.0	0.260
Fat (g)	N	-	Mann-Whitney-U test	6.7	5.0	0.431
Saturated fatty acids (g)	N	-	Mann-Whitney-U test	0.6	0.7	0.516
Carbohydrates (g)	Y	Y	T-test	11.2	10.0	0.404
Sugar (g)	Y	Y	T-test	8.9	8.4	0.346
Protein (g)	N	-	Mann-Whitney-U test	1.0	1.0	0.983
Salt (g)	Y	Y	T-test	1.2	1.1	0.662
Fiber (g)	N	-	Mann-Whitney-U test	2.3	2.2	0.040
Vitamin D (µg)	N	-	Mann-Whitney-U test	0.0	0.0	0.742
Vitamin E (µg)	N	-	Mann-Whitney-U test	2106.2	1510.5	0.113
Thiamine (µg)	N	-	Mann-Whitney-U test	46.6	41.0	0.123
Riboflavin (µg)	N	-	Mann-Whitney-U test	42.6	37.0	0.790
Vitamin B6 (µg)	N	-	Mann-Whitney-U test	156.0	124.0	0.038
Folate (µg)	N	-	Mann-Whitney-U test	20.9	20.8	0.840
Vitamin B12 (µg)	N	-	Mann-Whitney-U test	0.0	0.0	0.581
Vitamin C (µg)	N	-	Mann-Whitney-U test	30081.2	12520.7	0.145
Calcium (mg)	N	-	Mann-Whitney-U test	38.1	33.2	0.151
Magnesium (mg)	N	-	Mann-Whitney-U test	15.0	12.9	0.191
Iron (µg)	N	-	Mann-Whitney-U test	590.9	437.6	0.476
Zinc (µg)	N	-	Mann-Whitney-U test	253.6	236.4	0.389
Iodide (µg)	N	-	Mann-Whitney-U test	2.8	3.0	0.941
nutriRECIPE-Index (%)	N	-	Mann-Whitney-U test	47.3	48.8	0.671
MUFA (g)	N	-	Mann-Whitney-U test	3.0	2.1	0.269
PUFA (g)	N	-	Mann-Whitney-U test	1.5	1.2	0.078

HO: Homoscedasticity; MUFA: Monounsaturated fatty acids; N: No; ND: Normal distribution; PUFA: Polyunsaturated fatty acids; Y: Yes

Table S16. Comparison of nutriRECIPE-Index and nutrition content per 100 g according to the price range within raw food salads (n = 53).

				Criteria								
				Low-priced (a) [x < 0.60 €/100 g]			Medium-priced (b) [0.60 ≤ x ≤ 0.89 €/100 g]			High-priced (c) [x >0.89 €/100 g]		
Nutrient	ND	HO	Test	Median	p-Value to		Median	p-Value to		Median	p-Value to	
					b	c		a	c		a	b
Energy (kJ)	N	-	Kruskal-Wallis test	448.0	0.002	0.493	278.0	0.002	0.002	563.0	0.493	0.002
Fat (g)	N	-	Kruskal-Wallis test	5.0	0.013	0.622	2.8	0.013	0.017	8.7	0.622	0.017
Saturated fatty acids (g)	N	-	Kruskal-Wallis test	0.6	*	*	0.7	*	*	1.3	*	*
Carbohydrates (g)	Y	Y	One-way ANOVA	12.0	0.034	0.517	8.8	0.034	1.000	10.6	0.517	1.000
Sugar (g)	Y	Y	One-way ANOVA	10.5	0.009	0.000	6.6	0.009	0.903	6.8	0.000	0.903
Protein (g)	N	-	Kruskal-Wallis test	1.0	0.040	0.000	1.3	0.040	0.135	2.1	0.000	0.135
Salt (g)	Y	N	Welch's ANOVA	1.3	0.008	0.901	0.6	0.008	0.338	0.9	0.901	0.338
Fiber (g)	N	-	Kruskal-Wallis test	2.2	*	*	2.2	*	*	2.6	*	*
Vitamin D (µg)	N	-	Kruskal-Wallis test	0.0	*	*	0.0	*	*	0.0	*	*
Vitamin E (µg)	N	-	Kruskal-Wallis test	2177.6	0.006	0.029	1254.1	0.006	0.747	1107.7	0.029	0.747
Thiamine (µg)	N	-	Kruskal-Wallis test	41.0	*	*	43.3	*	*	50.0	*	*
Riboflavin (µg)	N	-	Kruskal-Wallis test	36.0	0.741	0.010	39.4	0.741	0.055	50.5	0.010	0.055
Vitamin B6 (µg)	N	-	Kruskal-Wallis test	137.7	2.000	2.000	154.9	2.000	2.000	105.1	2.000	2.000
Folate (µg)	N	-	Kruskal-Wallis test	20.0	*	*	21.5	*	*	24.3	*	*
Vitamin B12 (µg)	N	-	Kruskal-Wallis test	0.0	*	*	0.0	*	*	0.0	*	*
Vitamin C (µg)	N	-	Kruskal-Wallis test	26519.4	0.853	0.013	23558.3	0.853	0.024	8341.5	0.013	0.024
Calcium (mg)	N	-	Kruskal-Wallis test	35.7	*	*	27.1	*	*	39.4	*	*
Magnesium (mg)	N	-	Kruskal-Wallis test	13.2	*	*	12.6	*	*	23.6	*	*
Iron (µg)	N	-	Kruskal-Wallis test	464.8	0.227	0.027	413.7	0.227	0.005	959.9	0.027	0.005
Zinc (µg)	N	-	Kruskal-Wallis test	213.1	0.849	0.003	207.3	0.849	0.016	488.3	0.003	0.016
Iodide (µg)	N	-	Kruskal-Wallis test	2.6	*	*	9.0	*	*	14.4	*	*
nutriRECIPE-Index (%)	N	-	Kruskal-Wallis test	41.6	0.003	0.040	82.5	0.003	0.529	71.0	0.040	0.529
MUFA (g)	N	-	Kruskal-Wallis test	2.6	0.002	0.003	1.1	0.002	1.000	3.2	0.003	1.000
PUFA (g)	N	-	Kruskal-Wallis test	1.6	0.000	0.001	0.5	0.000	1.000	1.7	0.001	1.000

HO: Homoscedasticity; MUFA: Monounsaturated fatty acids; N: No; ND: Normal distribution; PUFA: Polyunsaturated fatty acids; Y: Yes

*: Multiple comparisons were not applied because the test showed no significant differences between samples

Table S17. Comparison of nutriRECIPE-Index and nutrition content per 100 g according to the salad base within starch-based salads (n = 89).

				Criteria															
				Semolina (a)				Potato (b)				Pasta (c)				Quinoa (d)			
Nutrient	ND	HO	Test	Median	p-Value to			Median	p-Value to			Median	p-Value to			Median	p-Value to		
					b	c	d		a	c	d		a	b	d		a	b	c
Energy (kJ)	N	-	Kruskal-Wallis test	769.0	0.000	0.272	0.000	593.0	0.000	0.000	0.393	880.5	0.272	0.000	0.035	673.0	0.000	0.393	0.035
Fat (g)	N	-	Kruskal-Wallis test	7.7	*	*	*	8.1	*	*	*	12.6	*	*	*	7.5	*	*	*
Saturated fatty acids (g)	N	-	Kruskal-Wallis test	0.7	0.172	0.000	0.000	0.9	0.172	0.003	0.658	1.6	0.000	0.003	0.126	1.0	0.000	0.658	0.126
Carbohydrates (g)	N	-	Kruskal-Wallis test	21.0	0.000	0.021	0.000	14.0	0.000	0.001	0.186	18.3	0.021	0.001	0.384	16.6	0.000	0.186	0.384
Sugar (g)	Y	N	Welch's ANOVA	3.4	0.838	0.090	0.996	4.2	0.838	0.266	1.000	5.2	0.090	0.266	0.891	4.8	0.996	1.000	0.891
Protein (g)	N	-	Kruskal-Wallis test	4.0	0.000	0.840	0.000	2.0	0.000	0.000	0.000	4.1	0.840	0.000	0.081	5.7	0.000	0.000	0.081
Salt (g)	N	-	Kruskal-Wallis test	1.3	*	*	*	1.4	*	*	*	1.4	*	*	*	1.3	*	*	*
Fiber (g)	N	-	Kruskal-Wallis test	3.7	0.000	0.000	0.000	1.3	0.000	0.051	0.000	2.0	0.000	0.051	0.000	5.2	0.000	0.000	0.000
Vitamin D (µg)	N	-	Kruskal-Wallis test	0.0	0.000	0.000	0.000	0.1	0.000	0.643	0.002	0.1	0.000	0.643	0.001	0.0	0.000	0.002	0.001
Vitamin E (µg)	N	-	Kruskal-Wallis test	2014.1	0.000	0.941	0.000	1278.6	0.000	0.000	0.422	2350.3	0.941	0.000	0.002	1127.1	0.000	0.422	0.002
Thiamine (µg)	N	-	Kruskal-Wallis test	102.7	0.000	0.000	0.000	63.1	0.000	0.605	0.000	71.7	0.000	0.605	0.000	128.4	0.000	0.000	0.000
Riboflavin (µg)	Y	Y	One-way ANOVA	62.7	0.000	1.000	1.000	23.8	0.000	0.001	0.000	58.8	1.000	0.001	1.000	59.6	1.000	0.000	1.000
Vitamin B6 (µg)	N	-	Kruskal-Wallis test	155.1	0.070	0.000	0.000	128.4	0.070	0.023	0.370	103.7	0.000	0.023	0.500	108.2	0.000	0.370	0.500
Folate (µg)	N	-	Kruskal-Wallis test	26.0	0.000	0.004	0.000	14.3	0.000	0.132	0.000	19.0	0.004	0.132	0.016	33.4	0.000	0.000	0.016
Vitamin B12 (µg)	N	-	Kruskal-Wallis test	0.0	0.000	0.000	0.000	0.1	0.000	0.217	0.014	0.1	0.000	0.217	0.002	0.0	0.000	0.014	0.002
Vitamin C (µg)	N	-	Kruskal-Wallis test	19962.7	0.002	0.026	0.000	13061.6	0.002	0.712	0.231	13548.1	0.026	0.712	0.170	9567.2	0.000	0.231	0.170
Calcium (mg)	N	-	Kruskal-Wallis test	34.2	0.000	0.215	0.000	20.2	0.000	0.010	0.000	28.9	0.215	0.010	0.014	47.7	0.000	0.000	0.014
Magnesium (mg)	N	-	Kruskal-Wallis test	42.1	0.000	0.000	0.000	19.5	0.000	0.988	0.000	18.4	0.000	0.988	0.000	49.6	0.000	0.000	0.000
Iron (µg)	N	-	Kruskal-Wallis test	1719.1	0.000	0.000	0.000	960.0	0.000	0.726	0.010	926.8	0.000	0.726	0.029	1434.1	0.000	0.010	0.029
Zinc (µg)	N	-	Kruskal-Wallis test	917.2	0.000	0.000	0.000	431.4	0.000	0.192	0.000	507.1	0.000	0.192	0.005	959.1	0.000	0.000	0.005
Iodide (µg)	N	-	Kruskal-Wallis test	2.7	*	*	*	3.2	*	*	*	2.1	*	*	*	3.1	*	*	*
nutriRECIPE-Index (%)	N	-	Kruskal-Wallis test	78.3	0.000	0.000	0.000	44.6	0.000	0.919	0.001	44.0	0.000	0.919	0.001	70.6	0.000	0.001	0.001
MUFA (g)	N	-	Kruskal-Wallis test	4.1	*	*	*	3.4	*	*	*	5.7	*	*	*	2.8	*	*	*
PUFA (g)	N	-	Kruskal-Wallis test	1.9	*	*	*	1.6	*	*	*	3.0	*	*	*	2.2	*	*	*

HO: Homoscedasticity; MUFA: Monounsaturated fatty acids; N: No; ND: Normal distribution; PUFA: Polyunsaturated fatty acids; Y: Yes

*: Multiple comparisons were not applied because the test showed no significant differences between samples

Table S18. Comparison of nutriRECIPE-Index and nutrition content per 100 g according to the salad base within semolina salads (n = 31).

				Criteria		
				Bulgur	Couscous	p-Value
Nutrient	ND	HO	Test	Median	Median	
Energy (kJ)	N	-	Mann-Whitney-U test	725.5	876.0	0.002
Fat (g)	N	-	Mann-Whitney-U test	5.8	11.0	0.001
Saturated fatty acids (g)	N	-	Mann-Whitney-U test	0.5	1.0	0.001
Carbohydrates (g)	N	-	Mann-Whitney-U test	22.6	21.0	0.532
Sugar (g)	Y	Y	T-test	3.3	4.1	0.589
Protein (g)	N	-	Mann-Whitney-U test	3.4	5.1	0.000
Salt (g)	N	-	Mann-Whitney-U test	1.2	1.4	0.617
Fiber (g)	N	-	Mann-Whitney-U test	4.0	3.3	0.011
Vitamin D (µg)	N	-	Mann-Whitney-U test	0.0	0.0	0.024
Vitamin E (µg)	N	-	Mann-Whitney-U test	1842.1	2514.0	0.000
Thiamine (µg)	N	-	Mann-Whitney-U test	103.4	103.4	0.771
Riboflavin (µg)	Y	N	Welch's test	63.0	62.8	0.611
Vitamin B6 (µg)	N	-	Mann-Whitney-U test	182.0	114.6	0.000
Folate (µg)	N	-	Mann-Whitney-U test	28.6	22.9	0.042
Vitamin B12 (µg)	N	-	Mann-Whitney-U test	0.0	0.0	0.024
Vitamin C (µg)	N	-	Mann-Whitney-U test	24002.8	15976.4	0.096
Calcium (mg)	N	-	Mann-Whitney-U test	30.8	42.4	0.339
Magnesium (mg)	N	-	Mann-Whitney-U test	48.6	33.7	0.008
Iron (µg)	N	-	Mann-Whitney-U test	1741.9	1622.0	0.157
Zinc (µg)	N	-	Mann-Whitney-U test	987.9	897.8	0.533
Iodide (µg)	N	-	Mann-Whitney-U test	1.9	3.3	0.228
nutriRECIPE-Index (%)	N	-	Mann-Whitney-U test	82.1	66.0	0.009
MUFA (g)	N	-	Mann-Whitney-U test	2.6	4.8	0.803
PUFA (g)	N	-	Mann-Whitney-U test	1.4	2.9	0.868

HO: Homoscedasticity; MUFA: Monounsaturated fatty acids; N: No; ND: Normal distribution; PUFA: Polyunsaturated fatty acids; Y: Yes

Table S19. Comparison of nutriRECIPE-Index and nutrition content per 100 g according to the cultivation method within semolina salads (n = 31).

				Criteria		
				Organic (+)	Organic (-)	p-Value
Nutrient	ND	HO	Test	Median	Median	
Energy (kJ)	N	-	Mann-Whitney-U test	769.0	751.0	0.800
Fat (g)	N	-	Mann-Whitney-U test	10.2	7.4	0.309
Saturated fatty acids (g)	N	-	Mann-Whitney-U test	1.5	0.6	0.014
Carbohydrates (g)	N	-	Mann-Whitney-U test	19.5	22.9	0.018
Sugar (g)	Y	Y	T-test	3.1	3.5	0.167
Protein (g)	N	-	Mann-Whitney-U test	3.5	4.0	0.280
Salt (g)	N	-	Mann-Whitney-U test	1.7	1.2	0.036
Fiber (g)	N	-	Mann-Whitney-U test	3.2	3.7	0.076
Vitamin D (µg)	N	-	Mann-Whitney-U test	0.0	0.0	0.147
Vitamin E (µg)	N	-	Mann-Whitney-U test	3085.8	2009.2	0.047
Thiamine (µg)	N	-	Mann-Whitney-U test	98.6	105.2	0.190
Riboflavin (µg)	Y	Y	T-test	62.1	68.0	0.521
Vitamin B6 (µg)	N	-	Mann-Whitney-U test	138.2	168.8	0.554
Folate (µg)	N	-	Mann-Whitney-U test	30.1	23.4	0.052
Vitamin B12 (µg)	N	-	Mann-Whitney-U test	0.0	0.0	0.147
Vitamin C (µg)	N	-	Mann-Whitney-U test	21617.2	19962.7	0.673
Calcium (mg)	N	-	Mann-Whitney-U test	29.3	35.3	0.205
Magnesium (mg)	N	-	Mann-Whitney-U test	35.8	49.0	0.069
Iron (µg)	N	-	Mann-Whitney-U test	1523.3	1762.3	0.220
Zinc (µg)	N	-	Mann-Whitney-U test	824.3	990.7	0.005
Iodide (µg)	N	-	Mann-Whitney-U test	32.4	1.9	0.000
nutriRECIPE-Index (%)	N	-	Mann-Whitney-U test	82.3	69.9	0.043
MUFA (g)	N	-	Mann-Whitney-U test	5.8	3.6	0.833
PUFA (g)	N	-	Mann-Whitney-U test	2.2	1.9	0.118

HO: Homoscedasticity; MUFA: Monounsaturated fatty acids; N: No; ND: Normal distribution; PUFA: Polyunsaturated fatty acids; Y: Yes

Table S20. Comparison of nutriRECIPE-Index and nutrition content per 100 g according to the brand within semolina salads (n = 31).

				Criteria		
				Private label	Branded label	p-Value
Nutrient	ND	HO	Test	Median	Median	
Energy (kJ)	N	-	Mann-Whitney-U test	776.0	769.0	0.937
Fat (g)	N	-	Mann-Whitney-U test	7.6	8.0	0.984
Saturated fatty acids (g)	N	-	Mann-Whitney-U test	0.7	0.7	0.857
Carbohydrates (g)	N	-	Mann-Whitney-U test	21.0	22.3	0.426
Sugar (g)	Y	Y	T-test	3.8	2.9	0.210
Protein (g)	N	-	Mann-Whitney-U test	3.4	4.1	0.080
Salt (g)	N	-	Mann-Whitney-U test	1.2	1.4	0.449
Fiber (g)	N	-	Mann-Whitney-U test	3.7	3.8	0.968
Vitamin D (µg)	N	-	Mann-Whitney-U test	0.0	0.0	0.733
Vitamin E (µg)	N	-	Mann-Whitney-U test	2115.6	1970.2	0.781
Thiamine (µg)	N	-	Mann-Whitney-U test	100.6	118.6	0.190
Riboflavin (µg)	Y	Y	T-test	62.2	65.3	0.983
Vitamin B6 (µg)	N	-	Mann-Whitney-U test	156.0	155.1	0.751
Folate (µg)	N	-	Mann-Whitney-U test	22.9	28.5	0.204
Vitamin B12 (µg)	N	-	Mann-Whitney-U test	0.0	0.0	0.838
Vitamin C (µg)	N	-	Mann-Whitney-U test	17423.7	22626.5	0.843
Calcium (mg)	N	-	Mann-Whitney-U test	42.4	27.4	0.751
Magnesium (mg)	N	-	Mann-Whitney-U test	42.8	36.2	0.937
Iron (µg)	N	-	Mann-Whitney-U test	1819.1	1678.1	0.578
Zinc (µg)	N	-	Mann-Whitney-U test	921.5	907.7	0.691
Iodide (µg)	N	-	Mann-Whitney-U test	2.8	2.7	0.552
nutriRECIPE-Index (%)	N	-	Mann-Whitney-U test	75.7	78.3	0.578
MUFA (g)	N	-	Mann-Whitney-U test	4.2	3.6	0.341
PUFA (g)	N	-	Mann-Whitney-U test	2.2	1.7	0.606

HO: Homoscedasticity; MUFA: Monounsaturated fatty acids; N: No; ND: Normal distribution; PUFA: Polyunsaturated fatty acids; Y: Yes

Table S21. Comparison of nutriRECIPE-Index and nutrition content per 100 g according to the price range within semolina salads (n = 31).

				Criteria								
				Low-priced (a) [x < 0.70 €/100 g]			Medium-priced (b) [0.70 ≤ x ≤1.25 €/100 g]			High-priced (c) [x > 1.25 €/100 g]		
Nutrient	ND	HO	Test	Median	p-Value to		Median	p-Value to		Median	p-Value to	
					b	c		a	C		a	b
Energy (kJ)	N	-	Kruskal-Wallis test	728.0	0.936	0.033	724.0	0.936	0.026	875.0	0.033	0.026
Fat (g)	N	-	Kruskal-Wallis test	5.9	0.967	0.008	6.1	0.967	0.003	11.0	0.008	0.003
Saturated fatty acids (g)	N	-	Kruskal-Wallis test	0.5	0.906	0.002	0.6	0.906	0.001	1.8	0.002	0.001
Carbohydrates (g)	N	-	Kruskal-Wallis test	24.0	0.518	0.018	21.6	0.518	0.054	18.0	0.018	0.054
Sugar (g)	Y	Y	One-way ANOVA	3.5	1.000	0.337	3.6	1.000	0.461	2.3	0.337	0.461
Protein (g)	N	-	Kruskal-Wallis test	3.3	0.024	0.025	4.6	0.024	0.846	5.0	0.025	0.846
Salt (g)	N	-	Kruskal-Wallis test	1.2	0.462	0.014	1.3	0.462	0.051	2.0	0.014	0.051
Fiber (g)	N	-	Kruskal-Wallis test	3.7	*	*	3.6	*	*	2.9	*	*
Vitamin D (µg)	N	-	Kruskal-Wallis test	0.0	0.038	1.000	0.0	0.038	0.038	0.0	1.000	0.038
Vitamin E (µg)	N	-	Kruskal-Wallis test	2014.1	0.758	0.018	1970.2	0.758	0.024	3288.5	0.018	0.024
Thiamine (µg)	N	-	Kruskal-Wallis test	101.7	*	*	104.2	*	*	102.7	*	*
Riboflavin (µg)	Y	Y	One-way ANOVA	55.2	0.337	0.581	73.7	0.337	1.000	62.1	0.581	1.000
Vitamin B6 (µg)	N	-	Kruskal-Wallis test	169.2	*	*	176.8	*	*	134.1	*	*
Folate (µg)	N	-	Kruskal-Wallis test	23.3	*	*	26.5	*	*	31.4	*	*
Vitamin B12 (µg)	N	-	Kruskal-Wallis test	0.0	0.038	1.000	0.0	0.038	0.038	0.0	1.000	0.038
Vitamin C (µg)	N	-	Kruskal-Wallis test	16358.9	*	*	22626.5	*	*	20198.8	*	*
Calcium (mg)	N	-	Kruskal-Wallis test	24.3	*	*	36.6	*	*	34.2	*	*
Magnesium (mg)	N	-	Kruskal-Wallis test	48.3	*	*	41.5	*	*	36.2	*	*
Iron (µg)	N	-	Kruskal-Wallis test	1678.1	*	*	1762.3	*	*	1754.6	*	*
Zinc (µg)	N	-	Kruskal-Wallis test	985.0	*	*	917.2	*	*	854.3	*	*
Iodide (µg)	N	-	Kruskal-Wallis test	1.2	0.038	0.000	2.3	0.038	0.016	34.0	0.000	0.016
nutriRECIPE-Index (%)	N	-	Kruskal-Wallis test	69.9	*	*	81.9	*	*	82.3	*	*
MUFA (g)	N	-	Kruskal-Wallis test	2.7	*	*	2.5	*	*	6.4	*	*
PUFA (g)	N	-	Kruskal-Wallis test	1.6	*	*	1.9	*	*	2.9	*	*

HO: Homoscedasticity; MUFA: Monounsaturated fatty acids; N: No; ND: Normal distribution; PUFA: Polyunsaturated fatty acids; Y: Yes

*: Multiple comparisons were not applied because the test showed no significant differences between samples

Table S22. Comparison of nutriRECIPE-Index and nutrition content per 100 g according to the diet within potato salads (n = 31).

				Criteria		
				Vegetarian (+)	Vegetarian (-)	p-Value
Nutrient	ND	HO	Test	Median	Median	
Energy (kJ)	N	-	Mann-Whitney-U test	591.0	704.5	0.042
Fat (g)	N	-	Mann-Whitney-U test	8.0	11.0	0.074
Saturated fatty acids (g)	N	-	Mann-Whitney-U test	0.8	1.5	0.001
Carbohydrates (g)	N	-	Mann-Whitney-U test	14.0	13.3	0.390
Sugar (g)	Y	Y	T-test	4.4	4.0	0.077
Protein (g)	N	-	Mann-Whitney-U test	1.6	2.6	0.009
Salt (g)	N	-	Mann-Whitney-U test	1.4	1.2	0.319
Fiber (g)	N	-	Mann-Whitney-U test	1.2	1.4	0.343
Vitamin D (µg)	N	-	Mann-Whitney-U test	0.1	0.2	0.201
Vitamin E (µg)	N	-	Mann-Whitney-U test	1261.6	1553.3	0.652
Thiamine (µg)	N	-	Mann-Whitney-U test	60.3	88.0	0.000
Riboflavin (µg)	Y	N	Welch's test	23.1	24.9	0.352
Vitamin B6 (µg)	N	-	Mann-Whitney-U test	125.8	145.9	0.004
Folate (µg)	N	-	Mann-Whitney-U test	14.3	16.8	0.684
Vitamin B12 (µg)	N	-	Mann-Whitney-U test	0.0	0.1	0.076
Vitamin C (µg)	N	-	Mann-Whitney-U test	13061.6	13109.6	0.684
Calcium (mg)	N	-	Mann-Whitney-U test	20.2	20.4	0.619
Magnesium (mg)	N	-	Mann-Whitney-U test	19.4	20.4	0.240
Iron (µg)	N	-	Mann-Whitney-U test	932.0	1046.0	0.416
Zinc (µg)	N	-	Mann-Whitney-U test	423.2	536.4	0.003
Iodide (µg)	N	-	Mann-Whitney-U test	3.2	3.5	0.223
nutriRECIPE-Index (%)	N	-	Mann-Whitney-U test	43.1	46.0	0.058
MUFA (g)	N	-	Mann-Whitney-U test	3.2	4.3	0.240
PUFA (g)	N	-	Mann-Whitney-U test	1.5	2.0	0.588

HO: Homoscedasticity; MUFA: Monounsaturated fatty acids; N: No; ND: Normal distribution; PUFA: Polyunsaturated fatty acids; Y: Yes

Table S23. Comparison of nutriRECIPE-Index and nutrition content per 100 g according to the dressing base within potato salads (n = 31).

				Criteria		
				Mayonnaise	Oil	p-Value
Nutrient	ND	HO	Test	Median	Median	
Energy (kJ)	N	-	Mann-Whitney-U test	643.5	493.0	0.000
Fat (g)	N	-	Mann-Whitney-U test	9.8	5.7	0.000
Saturated fatty acids (g)	N	-	Mann-Whitney-U test	1.0	0.5	0.001
Carbohydrates (g)	N	-	Mann-Whitney-U test	14.0	13.4	0.170
Sugar (g)	Y	Y	T-test	4.3	3.8	0.208
Protein (g)	N	-	Mann-Whitney-U test	2.3	1.5	0.063
Salt (g)	N	-	Mann-Whitney-U test	1.3	1.5	0.081
Fiber (g)	N	-	Mann-Whitney-U test	1.2	1.4	0.514
Vitamin D (µg)	N	-	Mann-Whitney-U test	0.2	0.0	0.000
Vitamin E (µg)	N	-	Mann-Whitney-U test	1673.9	1108.6	0.003
Thiamine (µg)	N	-	Mann-Whitney-U test	65.1	58.0	0.007
Riboflavin (µg)	Y	N	Welch's test	32.3	11.9	0.000
Vitamin B6 (µg)	N	-	Mann-Whitney-U test	129.3	128.2	0.663
Folate (µg)	N	-	Mann-Whitney-U test	17.2	12.0	0.001
Vitamin B12 (µg)	N	-	Mann-Whitney-U test	0.1	0.0	0.000
Vitamin C (µg)	N	-	Mann-Whitney-U test	12864.7	14297.6	0.017
Calcium (mg)	N	-	Mann-Whitney-U test	20.8	16.0	0.207
Magnesium (mg)	N	-	Mann-Whitney-U test	19.4	19.5	0.828
Iron (µg)	N	-	Mann-Whitney-U test	1017.0	725.6	0.009
Zinc (µg)	N	-	Mann-Whitney-U test	473.1	334.6	0.004
Iodide (µg)	N	-	Mann-Whitney-U test	3.4	2.9	0.019
nutriRECIPE-Index (%)	N	-	Mann-Whitney-U test	45.0	43.1	0.728
MUFA (g)	N	-	Mann-Whitney-U test	4.3	2.8	0.562
PUFA (g)	N	-	Mann-Whitney-U test	2.0	1.3	0.764

HO: Homoscedasticity; MUFA: Monounsaturated fatty acids; N: No; ND: Normal distribution; PUFA: Polyunsaturated fatty acids; Y: Yes

Table S24. Comparison of nutriRECIPE-Index and nutrition content per 100 g according to the brand within potato salads (n = 31).

				Criteria		
				Private label	Branded label	p-Value
Nutrient	ND	HO	Test	Median	Median	
Energy (kJ)	N	-	Mann-Whitney-U test	555.0	611.0	0.240
Fat (g)	N	-	Mann-Whitney-U test	7.1	8.4	0.446
Saturated fatty acids (g)	N	-	Mann-Whitney-U test	0.8	1.0	0.314
Carbohydrates (g)	N	-	Mann-Whitney-U test	14.0	14.0	1.000
Sugar (g)	Y	Y	T-test	3.7	4.3	0.651
Protein (g)	N	-	Mann-Whitney-U test	1.5	2.2	0.034
Salt (g)	N	-	Mann-Whitney-U test	1.3	1.4	0.631
Fiber (g)	N	-	Mann-Whitney-U test	1.5	1.2	0.728
Vitamin D (µg)	N	-	Mann-Whitney-U test	0.0	0.2	0.290
Vitamin E (µg)	N	-	Mann-Whitney-U test	1201.5	1450.8	0.317
Thiamine (µg)	N	-	Mann-Whitney-U test	58.7	63.8	0.055
Riboflavin (µg)	Y	Y	T-test	23.1	24.9	0.674
Vitamin B6 (µg)	N	-	Mann-Whitney-U test	125.7	129.3	0.828
Folate (µg)	N	-	Mann-Whitney-U test	12.7	15.6	0.139
Vitamin B12 (µg)	N	-	Mann-Whitney-U test	0.0	0.1	0.195
Vitamin C (µg)	N	-	Mann-Whitney-U test	12935.9	13313.7	0.384
Calcium (mg)	N	-	Mann-Whitney-U test	20.8	19.5	0.828
Magnesium (mg)	N	-	Mann-Whitney-U test	19.6	19.5	0.965
Iron (µg)	N	-	Mann-Whitney-U test	984.4	929.4	0.828
Zinc (µg)	N	-	Mann-Whitney-U test	399.1	451.4	0.151
Iodide (µg)	N	-	Mann-Whitney-U test	3.1	3.2	0.192
nutriRECIPE-Index (%)	N	-	Mann-Whitney-U test	41.8	45.0	0.338
MUFA (g)	N	-	Mann-Whitney-U test	3.2	3.8	0.728
PUFA (g)	N	-	Mann-Whitney-U test	1.5	1.8	0.486

HO: Homoscedasticity; MUFA: Monounsaturated fatty acids; N: No; ND: Normal distribution; PUFA: Polyunsaturated fatty acids; Y: Yes

Table S25. Comparison of nutriRECIPE-Index and nutrition content per 100 g according to the price range within potato salads (n = 31).

				Criteria								
				Low-priced (a) [x < 0.30 €/100 g]			Medium-priced (b) [0.30 ≤ x ≤0.45 €/100 g]			High-priced (c) [x > 0.45 €/100 g]		
Nutrient	ND	HO	Test	Median	p-Value to		Median	p-Value to		Median	p-Value to	
					b	c		a	c		a	b
Energy (kJ)	N	-	Kruskal-Wallis test	555.0	*	*	634.5	*	*	556.5	*	*
Fat (g)	N	-	Kruskal-Wallis test	7.1	*	*	9.4	*	*	7.6	*	*
Saturated fatty acids (g)	N	-	Kruskal-Wallis test	0.8	*	*	1.0	*	*	1.2	*	*
Carbohydrates (g)	N	-	Kruskal-Wallis test	14.4	*	*	13.7	*	*	13.2	*	*
Sugar (g)	Y	Y	One-way ANOVA	4.3	1.000	0.337	4.4	1.000	0.461	3.6	0.337	0.461
Protein (g)	N	-	Kruskal-Wallis test	1.5	*	*	2.2	*	*	2.5	*	*
Salt (g)	N	-	Kruskal-Wallis test	1.3	*	*	1.3	*	*	1.4	*	*
Fiber (g)	N	-	Kruskal-Wallis test	1.5	*	*	1.2	*	*	1.3	*	*
Vitamin D (µg)	N	-	Kruskal-Wallis test	0.0	*	*	0.1	*	*	0.2	*	*
Vitamin E (µg)	N	-	Kruskal-Wallis test	1201.5	*	*	1673.9	*	*	1294.8	*	*
Thiamine (µg)	N	-	Kruskal-Wallis test	60.3	*	*	63.1	*	*	69.0	*	*
Riboflavin (µg)	Y	N	Welch's ANOVA	23.1	0.130	0.399	30.3	0.130	0.985	23.0	0.399	0.985
Vitamin B6 (µg)	N	-	Kruskal-Wallis test	128.2	0.148	0.072	123.2	0.148	0.002	141.3	0.072	0.002
Folate (µg)	N	-	Kruskal-Wallis test	12.9	*	*	15.5	*	*	17.0	*	*
Vitamin B12 (µg)	N	-	Kruskal-Wallis test	0.0	*	*	0.1	*	*	0.1	*	*
Vitamin C (µg)	N	-	Kruskal-Wallis test	13061.6	*	*	12477.3	*	*	13989.8	*	*
Calcium (mg)	N	-	Kruskal-Wallis test	19.7	*	*	19.5	*	*	21.5	*	*
Magnesium (mg)	N	-	Kruskal-Wallis test	19.6	*	*	18.7	*	*	20.2	*	*
Iron (µg)	N	-	Kruskal-Wallis test	984.4	*	*	870.0	*	*	1071.9	*	*
Zinc (µg)	N	-	Kruskal-Wallis test	399.1	*	*	438.0	*	*	509.4	*	*
Iodide (µg)	N	-	Kruskal-Wallis test	3.2	*	*	3.2	*	*	10.0	*	*
nutriRECIPE-Index (%)	N	-	Kruskal-Wallis test	41.8	0.938	0.003	42.9	0.938	0.005	49.9	0.003	0.005
MUFA (g)	N	-	Kruskal-Wallis test	3.1	*	*	4.3	*	*	3.3	*	*
PUFA (g)	N	-	Kruskal-Wallis test	1.4	*	*	2.0	*	*	1.6	*	*

HO: Homoscedasticity; MUFA: Monounsaturated fatty acids; N: No; ND: Normal distribution; PUFA: Polyunsaturated fatty acids; Y: Yes

*: Multiple comparisons were not applied because the test showed no significant differences between samples

Table S26. Comparison of nutriRECIPE-Index and nutrition content per 100 g according to the diet within pasta salads (n = 18).

				Criteria		
				Vegetarian (+)	Vegetarian (-)	p-Value
Nutrient	ND	HO	Test	Median	Median	
Energy (kJ)	N	-	Mann-Whitney-U test	822.0	883.0	0.190
Fat (g)	N	-	Mann-Whitney-U test	7.6	16.0	0.031
Saturated fatty acids (g)	N	-	Mann-Whitney-U test	1.3	1.8	0.436
Carbohydrates (g)	N	-	Mann-Whitney-U test	21.1	14.6	0.024
Sugar (g)	Y	Y	T-test	5.2	3.9	0.015
Protein (g)	N	-	Mann-Whitney-U test	3.7	4.3	0.387
Salt (g)	N	-	Mann-Whitney-U test	1.3	1.5	0.796
Fiber (g)	N	-	Mann-Whitney-U test	2.8	1.4	0.136
Vitamin D (µg)	N	-	Mann-Whitney-U test	0.0	0.2	0.050
Vitamin E (µg)	N	-	Mann-Whitney-U test	1787.6	2534.5	0.222
Thiamine (µg)	N	-	Mann-Whitney-U test	71.4	73.1	0.489
Riboflavin (µg)	Y	Y	T-test	58.0	59.6	0.650
Vitamin B6 (µg)	N	-	Mann-Whitney-U test	103.5	106.5	0.863
Folate (µg)	N	-	Mann-Whitney-U test	19.3	18.7	0.931
Vitamin B12 (µg)	N	-	Mann-Whitney-U test	0.0	0.2	0.001
Vitamin C (µg)	N	-	Mann-Whitney-U test	19464.4	9180.6	0.040
Calcium (mg)	N	-	Mann-Whitney-U test	32.4	17.9	0.063
Magnesium (mg)	N	-	Mann-Whitney-U test	19.8	16.6	0.489
Iron (µg)	N	-	Mann-Whitney-U test	1008.1	734.2	0.222
Zinc (µg)	N	-	Mann-Whitney-U test	467.8	545.9	0.436
Iodide (µg)	N	-	Mann-Whitney-U test	2.4	2.0	0.730
nutriRECIPE-Index (%)	N	-	Mann-Whitney-U test	45.7	30.9	0.546
MUFA (g)	N	-	Mann-Whitney-U test	4.4	7.6	0.436
PUFA (g)	N	-	Mann-Whitney-U test	1.6	3.5	0.161

HO: Homoscedasticity; MUFA: Monounsaturated fatty acids; N: No; ND: Normal distribution; PUFA: Polyunsaturated fatty acids; Y: Yes

Table S27. Comparison of nutriRECIPE-Index and nutrition content per 100 g according to the dressing base within pasta salads (n = 18).

				Criteria		
				Mayonnaise	Pesto	p-Value
Nutrient	ND	HO	Test	Median	Median	
Energy (kJ)	N	-	Mann-Whitney-U test	930.0	696.0	0.003
Fat (g)	N	-	Mann-Whitney-U test	16.0	6.3	0.000
Saturated fatty acids (g)	N	-	Mann-Whitney-U test	1.9	0.9	0.052
Carbohydrates (g)	N	-	Mann-Whitney-U test	15.9	25.0	0.069
Sugar (g)	Y	Y	T-test	3.9	5.2	0.057
Protein (g)	N	-	Mann-Whitney-U test	3.7	4.5	0.377
Salt (g)	N	-	Mann-Whitney-U test	1.5	1.5	0.583
Fiber (g)	N	-	Mann-Whitney-U test	1.4	2.5	0.377
Vitamin D (μg)	N	-	Mann-Whitney-U test	0.1	0.0	0.009
Vitamin E (μg)	N	-	Mann-Whitney-U test	2788.3	1755.5	0.003
Thiamine (μg)	N	-	Mann-Whitney-U test	70.8	73.1	0.661
Riboflavin (μg)	Y	Y	T-test	58.0	58.0	0.759
Vitamin B6 (μg)	N	-	Mann-Whitney-U test	99.8	103.8	0.743
Folate (μg)	N	-	Mann-Whitney-U test	17.7	19.3	0.583
Vitamin B12 (μg)	N	-	Mann-Whitney-U test	0.2	0.0	0.038
Vitamin C (μg)	N	-	Mann-Whitney-U test	9180.6	20560.9	0.038
Calcium (mg)	N	-	Mann-Whitney-U test	18.3	40.0	0.038
Magnesium (mg)	N	-	Mann-Whitney-U test	16.6	26.2	0.090
Iron (μg)	N	-	Mann-Whitney-U test	774.4	1008.1	0.377
Zinc (μg)	N	-	Mann-Whitney-U test	530.6	636.1	0.510
Iodide (μg)	N	-	Mann-Whitney-U test	1.9	7.4	0.320
nutriRECIPE-Index (%)	N	-	Mann-Whitney-U test	30.0	60.1	0.069
MUFA (g)	N	-	Mann-Whitney-U test	7.5	2.6	0.743
PUFA (g)	N	-	Mann-Whitney-U test	3.5	1.6	0.583

HO: Homoscedasticity; MUFA: Monounsaturated fatty acids; N: No; ND: Normal distribution; PUFA: Polyunsaturated fatty acids; Y: Yes

Table S28. Comparison of nutriRECIPE-Index and nutrition content per 100 g according to the brand within pasta salads (n = 18).

				Criteria		
				Private label	Branded label	p-Value
Nutrient	ND	HO	Test	Median	Median	
Energy (kJ)	N	-	Mann-Whitney-U test	873.0	882.0	0.930
Fat (g)	N	-	Mann-Whitney-U test	12.1	13.0	0.930
Saturated fatty acids (g)	N	-	Mann-Whitney-U test	1.6	1.6	0.724
Carbohydrates (g)	N	-	Mann-Whitney-U test	19.0	17.0	0.860
Sugar (g)	Y	Y	T-test	5.4	5.1	0.666
Protein (g)	N	-	Mann-Whitney-U test	4.1	4.1	0.930
Salt (g)	N	-	Mann-Whitney-U test	1.5	1.3	0.930
Fiber (g)	N	-	Mann-Whitney-U test	2.5	1.5	0.659
Vitamin D (µg)	N	-	Mann-Whitney-U test	0.1	0.1	0.724
Vitamin E (µg)	N	-	Mann-Whitney-U test	2495.7	2204.9	0.930
Thiamine (µg)	N	-	Mann-Whitney-U test	73.1	71.4	1.000
Riboflavin (µg)	Y	Y	T-test	59.6	58.0	0.949
Vitamin B6 (µg)	N	-	Mann-Whitney-U test	114.7	103.5	0.246
Folate (µg)	N	-	Mann-Whitney-U test	20.6	18.7	0.211
Vitamin B12 (µg)	N	-	Mann-Whitney-U test	0.1	0.1	0.930
Vitamin C (µg)	N	-	Mann-Whitney-U test	15270.0	12140.5	0.659
Calcium (mg)	N	-	Mann-Whitney-U test	31.2	26.6	0.536
Magnesium (mg)	N	-	Mann-Whitney-U test	19.5	16.6	0.791
Iron (µg)	N	-	Mann-Whitney-U test	956.6	879.3	1.000
Zinc (µg)	N	-	Mann-Whitney-U test	545.9	468.3	0.791
Iodide (µg)	N	-	Mann-Whitney-U test	2.2	2.0	0.479
nutriRECIPE-Index (%)	N	-	Mann-Whitney-U test	49.5	42.2	0.860
MUFA (g)	N	-	Mann-Whitney-U test	5.7	5.0	0.596
PUFA (g)	N	-	Mann-Whitney-U test	3.0	3.3	0.536

HO: Homoscedasticity; MUFA: Monounsaturated fatty acids; N: No; ND: Normal distribution; PUFA: Polyunsaturated fatty acids; Y: Yes

Table S29. Comparison of nutriRECIPE-Index and nutrition content per 100 g according to the price range within pasta salads (n = 18).

				Criteria		
				Low-priced [x ≤ 0.50 €/100 g]	High-priced [x > 0.50 €/100 g]	p-Value
Nutrient	ND	HO	Test	Median	Median	
Energy (kJ)	N	-	Mann-Whitney-U test	957.0	759.0	0.002
Fat (g)	N	-	Mann-Whitney-U test	16.1	7.0	0.000
Saturated fatty acids (g)	N	-	Mann-Whitney-U test	2.1	1.2	0.002
Carbohydrates (g)	N	-	Mann-Whitney-U test	14.3	19.4	0.034
Sugar (g)	Y	Y	T-test	4.0	5.2	0.185
Protein (g)	N	-	Mann-Whitney-U test	3.7	4.4	0.146
Salt (g)	N	-	Mann-Whitney-U test	1.5	1.2	0.083
Fiber (g)	N	-	Mann-Whitney-U test	1.2	2.6	0.016
Vitamin D (µg)	N	-	Mann-Whitney-U test	0.1	0.0	0.173
Vitamin E (µg)	N	-	Mann-Whitney-U test	2956.7	1758.2	0.001
Thiamine (µg)	N	-	Mann-Whitney-U test	75.5	71.7	0.829
Riboflavin (µg)	Y	Y	T-test	53.2	62.2	0.095
Vitamin B6 (µg)	N	-	Mann-Whitney-U test	83.7	118.2	0.021
Folate (µg)	N	-	Mann-Whitney-U test	15.3	20.0	0.101
Vitamin B12 (µg)	N	-	Mann-Whitney-U test	0.1	0.1	0.203
Vitamin C (µg)	N	-	Mann-Whitney-U test	8677.0	17915.4	0.021
Calcium (mg)	N	-	Mann-Whitney-U test	17.9	39.6	0.002
Magnesium (mg)	N	-	Mann-Whitney-U test	14.1	23.6	0.003
Iron (µg)	N	-	Mann-Whitney-U test	703.9	1029.2	0.043
Zinc (µg)	N	-	Mann-Whitney-U test	461.5	606.7	0.203
Iodide (µg)	N	-	Mann-Whitney-U test	1.8	5.0	0.002
nutriRECIPE-Index (%)	N	-	Mann-Whitney-U test	28.8	55.1	0.001
MUFA (g)	N	-	Mann-Whitney-U test	7.6	3.5	0.897
PUFA (g)	N	-	Mann-Whitney-U test	3.6	1.9	0.829

HO: Homoscedasticity; MUFA: Monounsaturated fatty acids; N: No; ND: Normal distribution; PUFA: Polyunsaturated fatty acids; Y: Yes

Table S30. Comparison of nutriRECIPE-Index and nutrition content per 100 g according to the source of protein within protein-based salads (n = 45).

				Criteria		
				Animal-based	Plant-based	p-Value
Nutrient	ND	HO	Test	Median	Median	
Energy (kJ)	Y	N	Welch's test	1138.5	487.0	0.000
Fat (g)	Y	N	Welch's test	24.8	3.3	0.000
Saturated fatty acids (g)	N	-	Mann-Whitney-U test	3.8	0.7	0.000
Carbohydrates (g)	N	-	Mann-Whitney-U test	5.9	12.0	0.000
Sugar (g)	Y	Y	T-test	4.6	3.4	0.078
Protein (g)	Y	Y	T-test	7.5	6.6	0.034
Salt (g)	Y	Y	T-test	1.5	1.1	0.002
Fiber (g)	N	-	Mann-Whitney-U test	0.6	3.3	0.000
Vitamin D (µg)	N	-	Mann-Whitney-U test	0.1	0.0	0.000
Vitamin E (µg)	N	-	Mann-Whitney-U test	3262.3	752.6	0.000
Thiamine (µg)	N	-	Mann-Whitney-U test	119.8	119.4	0.887
Riboflavin (µg)	N	-	Mann-Whitney-U test	93.7	85.8	0.192
Vitamin B6 (µg)	N	-	Mann-Whitney-U test	119.7	123.5	0.691
Folate (µg)	N	-	Mann-Whitney-U test	10.3	34.0	0.005
Vitamin B12 (µg)	N	-	Mann-Whitney-U test	0.5	0.0	0.000
Vitamin C (µg)	N	-	Mann-Whitney-U test	3150.1	15415.8	0.012
Calcium (mg)	N	-	Mann-Whitney-U test	31.9	52.5	0.001
Magnesium (mg)	N	-	Mann-Whitney-U test	15.2	30.4	0.000
Iron (µg)	Y	Y	T-test	1054.2	1726.6	0.002
Zinc (µg)	Y	Y	T-test	898.5	676.8	0.041
Iodide (µg)	N	-	Mann-Whitney-U test	2.6	3.5	0.570
nutriRECIPE-Index	N	-	Mann-Whitney-U test	47.2	91.8	0.000
MUFA (g)	Y	Y	T-test	8.9	1.4	0.336
PUFA (g)	N	-	Mann-Whitney-U test	4.3	1.3	0.228

HO: Homoscedasticity; MUFA: Monounsaturated fatty acids; N: No; ND: Normal distribution; PUFA: Polyunsaturated fatty acids; Y: Yes

Table S31. Comparison of nutriRECIPE-Index and nutrition content per 100 g according to the salad base within protein-based salads (n = 45).

				Criteria															
				Red meat (a)				Poultry (b)				Eggs (c)				Legumes (d)			
Nutrient	ND	HO	Test	Median	p-Value to			Median	p-Value to			Median	p-Value to			Median	p-Value to		
					b	c	d		a	c	d		a	b	d		a	b	c
Energy (kJ)	Y	N	Welch's ANOVA	1129.0	1.000	0.540	0.000	988.0	1.000	0.368	0.000	1231.5	0.540	0.368	0.000	487.0	0.000	0.000	0.000
Fat (g)	Y	N	Welch's ANOVA	25.0	0.935	0.643	0.000	19.8	0.935	0.206	0.000	26.9	0.643	0.206	0.000	3.3	0.000	0.000	0.000
Saturated fatty acids (g)	N	-	Kruskal-Wallis test	4.9	0.001	0.234	0.000	1.9	0.001	0.099	0.169	3.2	0.234	0.099	0.003	0.7	0.000	0.169	0.003
Carbohydrates (g)	N	-	Kruskal-Wallis test	5.9	0.396	0.185	0.001	6.2	0.396	0.063	0.024	4.9	0.185	0.063	0.000	12.0	0.001	0.024	0.000
Sugar (g)	Y	Y	One-way ANOVA	4.0	0.706	1.000	0.728	5.5	0.706	0.164	0.056	3.9	1.000	0.164	1.000	3.4	0.728	0.056	1.000
Protein (g)	Y	Y	One-way ANOVA	5.4	0.007	0.073	1.000	9.6	0.007	1.000	0.004	8.6	0.073	1.000	0.031	6.6	1.000	0.004	0.031
Salt (g)	Y	Y	One-way ANOVA	1.7	0.019	0.079	0.000	1.2	0.019	1.000	1.000	1.4	0.079	1.000	0.595	1.1	0.000	1.000	0.595
Fiber (g)	N	-	Kruskal-Wallis test	0.7	0.155	0.515	0.000	1.6	0.155	0.080	0.043	0.4	0.515	0.080	0.000	3.3	0.000	0.043	0.000
Vitamin D (µg)	N	-	Kruskal-Wallis test	0.1	0.334	0.000	0.028	0.1	0.334	0.012	0.007	1.9	0.000	0.012	0.000	0.0	0.028	0.007	0.000
Vitamin E (µg)	N	-	Kruskal-Wallis test	3159.6	0.915	0.049	0.002	2757.1	0.915	0.105	0.006	5045.8	0.049	0.105	0.000	752.6	0.002	0.006	0.000
Thiamine (µg)	N	-	Kruskal-Wallis test	198.3	0.000	0.004	0.069	48.3	0.000	0.078	0.005	83.0	0.004	0.078	0.323	119.4	0.069	0.005	0.323
Riboflavin (µg)	N	-	Kruskal-Wallis test	88.1	0.677	0.000	0.662	78.1	0.677	0.000	0.986	265.0	0.000	0.000	0.000	85.8	0.662	0.986	0.000
Vitamin B6 (µg)	N	-	Kruskal-Wallis test	148.2	0.425	0.000	0.437	120.4	0.425	0.004	0.986	73.0	0.000	0.004	0.004	123.5	0.437	0.986	0.004
Folate (µg)	N	-	Kruskal-Wallis test	8.1	0.667	0.000	0.000	11.2	0.000	0.000	0.004	47.2	0.000	0.000	0.455	34.0	0.000	0.004	0.455
Vitamin B12 (µg)	N	-	Kruskal-Wallis test	0.4	0.032	0.024	0.000	0.2	0.032	0.000	0.078	1.2	0.024	0.000	0.000	0.0	0.000	0.078	0.000
Vitamin C (µg)	N	-	Kruskal-Wallis test	12266.5	0.167	0.002	0.211	3331.8	0.167	0.114	0.024	424.2	0.002	0.114	0.000	15415.8	0.211	0.024	0.000
Calcium (mg)	N	-	Kruskal-Wallis test	20.2	0.721	0.014	0.000	31.8	0.000	0.065	0.004	44.8	0.014	0.065	0.328	52.5	0.000	0.004	0.328
Magnesium (mg)	N	-	Kruskal-Wallis test	17.3	0.774	0.032	0.002	16.2	0.774	0.036	0.015	12.7	0.032	0.036	0.000	30.4	0.002	0.015	0.000
Iron (µg)	Y	Y	One-way ANOVA	863.7	1.000	0.021	0.000	1146.8	1.000	0.667	0.062	1370.2	0.021	0.667	1.000	1726.6	0.000	0.062	1.000
Zinc (µg)	Y	Y	One-way ANOVA	902.1	0.378	1.000	0.114	676.2	0.378	0.588	1.000	1067.4	1.000	0.588	0.233	676.8	0.114	1.000	0.233
Iodide (µg)	N	-	Kruskal-Wallis test	2.0	0.079	0.000	0.076	4.4	0.000	0.112	0.986	13.4	0.000	0.112	0.116	3.5	0.076	0.986	0.116
nutriRECIPE-Index	N	-	Kruskal-Wallis test	37.7	0.712	0.029	0.000	46.3	0.000	0.027	0.000	63.3	0.029	0.027	0.087	91.8	0.000	0.000	0.087
MUFA (g)	Y	Y	One-way ANOVA	10.6	0.712	0.029	0.000	8.1	*	*	*	11.4	*	*	*	1.4	*	*	*
PUFA (g)	N	-	Kruskal-Wallis test	4.2	0.712	0.029	0.000	4.1	*	*	*	5.6	*	*	*	1.3	*	*	*

HO: Homoscedasticity; MUFA: Monounsaturated fatty acids; N: No; ND: Normal distribution; PUFA: Polyunsaturated fatty acids; Y: Yes

*: Multiple comparisons were not applied because the test showed no significant differences between samples

Table S32. Comparison of nutriRECIPE-Index and nutrition content per 100 g according to the dressing base within meat-based salads (n = 28).

				Criteria		
				Vinaigrette	Mayonnaise/ Yoghurt	p-Value
Nutrient	ND	HO	Test	Median	Median	
Energy (kJ)	Y	Y	T-test	692.5	1189.0	0.000
Fat (g)	Y	Y	T-test	13.0	26.7	0.000
Saturated fatty acids (g)	N	-	Mann-Whitney-U test	5.0	3.5	0.328
Carbohydrates (g)	N	-	Mann-Whitney-U test	3.8	6.1	0.136
Sugar (g)	Y	N	Welch's test	3.1	5.0	0.705
Protein (g)	Y	N	Welch's test	8.0	5.8	0.073
Salt (g)	Y	Y	T-test	2.0	1.4	0.001
Fiber (g)	N	-	Mann-Whitney-U test	1.0	0.6	0.672
Vitamin D (μg)	N	-	Mann-Whitney-U test	0.0	0.1	0.001
Vitamin E (μg)	N	-	Mann-Whitney-U test	820.0	3481.1	0.000
Thiamine (μg)	N	-	Mann-Whitney-U test	335.7	132.7	0.003
Riboflavin (μg)	N	-	Mann-Whitney-U test	114.2	74.9	0.000
Vitamin B6 (μg)	N	-	Mann-Whitney-U test	286.8	119.7	0.000
Folate (μg)	N	-	Mann-Whitney-U test	8.2	8.2	0.533
Vitamin B12 (μg)	N	-	Mann-Whitney-U test	0.8	0.3	0.000
Vitamin C (μg)	N	-	Mann-Whitney-U test	28685.3	2851.7	0.000
Calcium (mg)	N	-	Mann-Whitney-U test	22.3	26.5	0.823
Magnesium (mg)	N	-	Mann-Whitney-U test	19.9	15.1	0.021
Iron (μg)	Y	Y	T-test	632.7	1054.2	0.079
Zinc (μg)	Y	Y	T-test	1202.7	788.1	0.003
Iodide (μg)	N	-	Mann-Whitney-U test	2.6	1.8	0.099
nutriRECIPE-Index	N	-	Mann-Whitney-U test	58.6	35.3	0.000
MUFA (g)	Y	Y	T-test	6.7	10.9	0.024
PUFA (g)	N	-	Mann-Whitney-U test	2.0	5.0	0.028

HO: Homoscedasticity; MUFA: Monounsaturated fatty acids; N: No; ND: Normal distribution; PUFA: Polyunsaturated fatty acids; Y: Yes

Table S33. Comparison of nutriRECIPE-Index and nutrition content per 100 g according to the brand within meat-based salads (n = 28).

				Criteria		
				Private label	Branded label	p-Value
Nutrient	ND	HO	Test	Median	Median	
Energy (kJ)	Y	Y	T-test	1194.5	907.0	0.075
Fat (g)	Y	Y	T-test	25.8	18.1	0.083
Saturated fatty acids (g)	N	-	Mann-Whitney-U test	4.1	2.9	0.347
Carbohydrates (g)	N	-	Mann-Whitney-U test	6.1	5.8	0.837
Sugar (g)	Y	Y	T-test	4.6	5.5	0.605
Protein (g)	Y	Y	T-test	6.8	7.1	0.660
Salt (g)	Y	Y	T-test	1.5	1.6	0.299
Fiber (g)	N	-	Mann-Whitney-U test	0.5	1.7	0.007
Vitamin D (µg)	N	-	Mann-Whitney-U test	0.1	0.1	0.802
Vitamin E (µg)	N	-	Mann-Whitney-U test	3585.6	2721.4	0.100
Thiamine (µg)	N	-	Mann-Whitney-U test	150.8	132.3	0.873
Riboflavin (µg)	N	-	Mann-Whitney-U test	81.4	89.6	0.121
Vitamin B6 (µg)	N	-	Mann-Whitney-U test	127.0	199.0	0.174
Folate (µg)	N	-	Mann-Whitney-U test	7.6	10.1	0.082
Vitamin B12 (µg)	N	-	Mann-Whitney-U test	0.3	0.5	0.302
Vitamin C (µg)	N	-	Mann-Whitney-U test	3328.6	8936.1	0.110
Calcium (mg)	N	-	Mann-Whitney-U test	19.5	32.3	0.053
Magnesium (mg)	N	-	Mann-Whitney-U test	15.1	21.4	0.003
Iron (µg)	Y	Y	T-test	840.6	1238.0	0.009
Zinc (µg)	Y	N	Welch's test	819.0	996.6	0.167
Iodide (µg)	N	-	Mann-Whitney-U test	2.2	2.5	0.507
nutriRECIPE-Index	N	-	Mann-Whitney-U test	35.1	49.1	0.042
MUFA (g)	Y	Y	T-test	9.8	8.0	0.112
PUFA (g)	N	-	Mann-Whitney-U test	5.0	3.3	0.159

HO: Homoscedasticity; MUFA: Monounsaturated fatty acids; N: No; ND: Normal distribution; PUFA: Polyunsaturated fatty acids; Y: Yes

Table S34. Comparison of nutriRECIPE-Index and nutrition content per 100 g according to the price range within meat-based salads (n = 28).

				Criteria		
				Low-priced [x < 0.70 €/100 g]	High-priced [x ≥ 0.70 €/100 g]	p-Value
Nutrient	ND	HO	Test	Median	Median	
Energy (kJ)	Y	Y	T-test	1189.0	930.0	0.114
Fat (g)	Y	Y	T-test	26.6	18.5	0.073
Saturated fatty acids (g)	N	-	Mann-Whitney-U test	4.6	2.2	0.161
Carbohydrates (g)	N	-	Mann-Whitney-U test	6.0	6.0	0.963
Sugar (g)	Y	N	Welch's test	4.5	5.5	0.335
Protein (g)	Y	Y	T-test	5.3	8.6	0.026
Salt (g)	Y	Y	T-test	1.6	1.6	0.232
Fiber (g)	N	-	Mann-Whitney-U test	0.7	1.4	0.161
Vitamin D (µg)	N	-	Mann-Whitney-U test	0.1	0.1	0.353
Vitamin E (µg)	N	-	Mann-Whitney-U test	3205.0	2723.2	0.404
Thiamine (µg)	N	-	Mann-Whitney-U test	153.7	67.5	0.404
Riboflavin (µg)	N	-	Mann-Whitney-U test	73.5	91.2	0.066
Vitamin B6 (µg)	N	-	Mann-Whitney-U test	129.2	215.8	0.122
Folate (µg)	N	-	Mann-Whitney-U test	7.7	10.0	0.208
Vitamin B12 (µg)	N	-	Mann-Whitney-U test	0.4	0.5	0.353
Vitamin C (µg)	N	-	Mann-Whitney-U test	3331.8	12368.5	0.029
Calcium (mg)	N	-	Mann-Whitney-U test	20.2	26.5	0.817
Magnesium (mg)	N	-	Mann-Whitney-U test	15.4	17.3	0.225
Iron (µg)	Y	Y	T-test	928.6	1092.5	0.415
Zinc (µg)	Y	N	Welch's test	845.3	957.1	0.215
Iodide (µg)	N	-	Mann-Whitney-U test	1.8	4.0	0.013
nutriRECIPE-Index	N	-	Mann-Whitney-U test	34.1	51.9	0.015
MUFA (g)	Y	Y	T-test	10.6	7.9	0.011
PUFA (g)	N	-	Mann-Whitney-U test	4.4	3.3	0.073

HO: Homoscedasticity; MUFA: Monounsaturated fatty acids; N: No; ND: Normal distribution; PUFA: Polyunsaturated fatty acids; Y: Yes
